# The burden of multidrug-resistant tuberculosis and extensively drug-resistant tuberculosis and its attributable risk factors in 204 countries and territories, 1990–2021

**DOI:** 10.3389/fpubh.2026.1960895

**Published:** 2026-10-26

**Authors:** Hongping Wang, Shan Lv, Pu Yao, Jing Cen

**Affiliations:** Department of Pharmacy, The First Affiliated Hospital of Army Medical University (Third Military Medical University), Chongqing, China

**Keywords:** multidrug-resistant tuberculosis, extensively drug-resistant tuberculosis, HIV/AIDS status, epidemiology, disease burden, GBD database

## Abstract

**Objective:**

Anti-tuberculosis drug resistance has become a major challenge to global tuberculosis control. This study aims to comprehensively the global burden, trends, and risks for disease deaths of multidrug-resistant tuberculosis (MDR-TB), extensively drug-resistant tuberculosis (XDR-TB), HIV/AIDS-MDR-TB, and HIV/AIDS-XDR-TB from 1990 to 2021.

**Methods:**

Data on the incidence and deaths of MDR-TB, XDR-TB, HIV/AIDS-MDR-TB, and HIV/AIDS-XDR-TB were obtained from GBD 2021. The estimated annual percentage change (EAPC) and fold change were calculated to assess the trends of disease burden changes from baseline (1990 for MDR-TB and HIV/AIDS-MDR-TB; 1993 for XDR-TB and HIV/AIDS-XDR-TB) to 2021.

**Results:**

In 2021, the highest age-standardized incidence rates (ASIR) and age-standardized death rates (ASDR) were observed in MDR-TB globally, with 5.4161 (3.1676–9.3429) and 1.2791 (0.4992–2.5331) per 100,000 population, respectively. A significant increase in the global incidence and death burden of MDR-TB, XDR-TB, HIV/AIDS-MDR-TB, and HIV/AIDS-XDR-TB has been reported from baseline to 2021. Especially for HIV/AIDS-XDR-TB, death cases increased 84.89-fold, with an EAPC in ASDR of 10 (6.26–13.86). In contrast, MDR-TB demonstrated a comparatively smaller increase, with death cases rising 5.65-fold and an EAPC in ASDR of 1.18 (−0.37–2.75). Over the past 32 years, the incidence and death ratio of these types of DR-TB in the High-middle SDI region have been the highest. In terms of age patterns, the burden of MDR-TB and XDR-TB has increased most significantly in the 15–49 age group. In contrast, the burden of HIV/AIDS-MDR-TB and HIV/AIDS-XDR-TB has notably increased among those aged 5–14.

**Conclusion:**

Over the past 30 years, a significant increase in the global burden of MDR-TB, XDR-TB, HIV/AIDS-MDR-TB, and HIV/AIDS-XDR-TB has been reported, with marked variations in various resistance types across different regions and countries. In particular, the high-middle SDI regions warrant special attention. These findings highlight the need for further research to identify effective intervention strategies tailored to the epidemiological patterns observed in different regions and populations.

## Introduction

Tuberculosis (TB) is a chronic infectious disease caused by *Mycobacterium tuberculosis (Mtb)*, primarily transmitted through airborne particles (1). Yet in 2023, returned to being the world’s leading cause of death from a single infectious agent, surpassing infections caused by coronavirus disease (COVID-19), It is also the leading cause of death for people living with HIV and a major cause of death related to antibiotic resistance (1, 2). In recent years, the threat of drug-resistant TB (DR-TB), primarily multidrug-resistant tuberculosis (MDR-TB) and extensively drug-resistant tuberculosis (XDR-TB), has escalated and become a major challenge to global TB control (3). The World Health Organization (WHO) estimated number in 2022 was 410,000 cases of MDR-TB or rifampicin-resistance TB (MDR/RR-TB), which was 3.3% among new cases and 17% among those previously treated. Of these, 160,000 people died (4). MDR-TB is estimated to cause 13% of all antimicrobial resistance-attributable deaths worldwide. The proportion of individuals exposed to MDR-TB remains unclear. Mathematical modeling suggests that approximately 19 million people have a latent MDR-TB infection and are at risk of activation (5). Regrettably, global data on confirmed or potential XDR-TB cases is scarce.

The persistent intrinsic resistance and acquired transmission of (DR-TB) have led to declining cure rates and reduced survival times, with these rates varying across different countries and public health systems (6, 7). The overall cure rates of MDR-TB and XDR-TB were only 68 and 27%, respectively (1, 8), both far below the WHO’s target rate of 75%. Additionally, due to the lack of safe and effective treatment options, the long treatment duration (over 18 months), and the high cost of care, DR-TB treatment expenses are 20 times higher than those of drug-susceptible TB, has consumed over 50% of national TB budgets and imposes a significant burden on healthcare systems (9). Moreover, relevant studies have shown that DR-TB is a debilitating disease that can give rise to severe and long-lasting physical, mental, and financial sequelae (10, 11). Patients with DR-TB are also more likely to experience stigma and social isolation compared to those with susceptible TB (12). The disruptive impact of the COVID-19 pandemic on access to TB diagnosis and treatment has further exacerbated the disease burden. In addition, some risk factors, such as smoking, alcohol consumption, high fasting plasma glucose (HFPG), etc., have been identified as contributing to adverse outcomes in DR-TB, particularly in countries with a high burden of DR-TB (13, 14). Therefore, a comprehensive understanding of the burden and epidemiological trends of MDR-TB and XDR-TB is crucial for assessing progress toward ending the epidemic and for guiding the formulation and implementation of TB prevention and control strategies. This is also essential for achieving the WHO’s End TB Strategy and the United Nations Sustainable Development Goals (SDGs).

Although previous GBD-based analyses have examined selected aspects of the DR-TB burden (15–19), several important gaps remain. Several existing studies on the burden of MDR-TB and XDR-TB were restricted to HIV-negative individuals (15–17), and another focused solely on the incidence of individuals (18). In addition, The GBD 2021 TB Collaborators reported age-specific progress toward the WHO End TB milestones (19), but did not simultaneously compare MDR-TB, XDR-TB, HIV/AIDS-MDR-TB, and HIV/AIDS-XDR-TB across all 204 countries and territories with risk factor attribution. Therefore, we conducted an updated and comprehensive assessment of the incidence and deaths of MDR-TB, XDR-TB, HIV/AIDS-MDR-TB, and HIV/AIDS-XDR-TB from 1990 to 2021 (XDR-TB and HIV/AIDS-XDR-TB from 1993 to 2021) at global, regional, and national levels. We also analyzed differences by sex and age group and quantified the risk factors attributable to death from these diseases.

## Methods

### Overview

Drawing on globally representative and reliable data sources, GBD 2021 study estimated the disease burden for 371 diseases and injuries, as well as 88 risk factors across 204 countries or territories. All data are freely accessible through the Global Health Data Exchange query tool^1^ (20, 21). This study conducted a secondary analysis of GBD 2021 estimates of incidence and death for MDR-TB, XDR-TB, HIV/AIDS-MDR-TB, and HIV/AIDS-XDR-TB from 1990 to 2021, using methods detailed in the GBD 2021 methodology (20, 21). The data were categorized based on global trends, Socio-Demographic Index (SDI), 21 regions, individual countries, gender, and age groups. Notably, the baseline year is 1990 for MDR-TB and HIV/AIDS-MDR-TB, and 1993 for XDR-TB and HIV/AIDS-XDR-TB (19, 21). The estimates extracted from GBD 2021 included both point estimates and 95% uncertainty intervals (UIs).

### Case definition

Tuberculosis (TB) includes all forms, including bacteriologically confirmed or clinically diagnosed pulmonary and extrapulmonary TB. In the GBD 2021 study, the International Classification of Diseases, 10th Revision (ICD-10) codes for TB are A10-A19.9, B90-B90.9, K67.3, K93.0, M49.0, N74.1, P37.0, U84.3, and ICD-9 codes are 010–019.9, 137–137.9, 138.0, 138.9, 320.4, and 730.4–730.6. The ICD-10 code for HIV/AIDS-TB is B20.0 (21).

GBD 2021 estimated the incidence and deaths of MDR-TB and XDR-TB by HIV status. It is important to note that the GBD 2021 study adopted the pre-2021 WHO classification for drug-resistant TB, which was defined based on resistance to second-line injectable drugs. Under the GBD 2021 framework: MDR-TB is defined as a form of TB (among HIV-negative individuals) that is resistant to the two most effective first-line anti-tuberculosis drugs (isoniazid and rifampicin) but is not resistant to any fluoroquinolone and any second-line injectable drugs (amikacin, kanamycin, or capreomycin). XDR-TB is defined as a form of TB (among HIV-negative individuals) that is resistant to isoniazid and rifampicin, plus any fluoroquinolone and any second-line injectable drugs. HIV/AIDS-MDR-TB is defined as MDR-TB among HIV-positive individuals. HIV/AIDS-XDR-TB is defined as XDR-TB among HIV-positive individuals (19, 21).

### Risk factors

The GBD 2021 risk factor analysis utilized data from 54,561 distinct sources to generate epidemiological estimates for 88 risk factors and their associated health outcomes, covering a total of 631 risk-outcome pairs. The GBD 2021 framework categorizes risk factors into four levels, encompassing a total of 88 overarching risks, with detailed information provided in Supplementary Table S1 (20). This study examines the death risk proportion for MDR-TB, XDR-TB, HIV/AIDS-MDR-TB, and HIV/AIDS-XDR-TB in 2021, analyzing patterns by sex at both global and regional levels. In the analysis of MDR-TB and XDR-TB, six key risk factors were emphasized: smoking, high fasting plasma glucose, high alcohol consumption, high body mass index, low physical activity, and a diet high in processed meat. In contrast, the analysis of HIV/AIDS-MDR-TB and HIV/AIDS-XDR-TB primarily focused on three risk factors: unsafe sex, drug use, and intimate partner violence. Detailed GBD definitions, estimation methodology, and interpretation of the proportion for these risk factors are provided in Supplementary Table S2.

### Statistical analysis

This study employs EAPC to assess the trends in age-standardized incidence rates (ASIR) of incidence and age-standardized death rates (ASDR) for MDR-TB, XDR-TB, HIV/AIDS-MDR-TB, and HIV/AIDS-XDR-TB from baseline year to 2021. The calculation of EAPC is based on fitting the natural logarithm rate of the regression model, with time as a variable, fitting the natural logarithm of each observation into a straight line, and calculating based on the slope of this line (22). The formula is as follows:


y=α+βx+ε

$$\text{EAPC}=100\times (e^{\beta }-1).$$

In this equation, y—is the natural logarithm of the age-standardized rate (ln(ASR)), x-year, *α*-the intercept, *β*-the slope, and *ε*-the random error. The 95% confidence intervals (CIs) of the EAPC were derived from the regression model and used to quantify the uncertainty of the trend estimates. The statistical significance of trend changes is determined based on EAPC: when both the EAPC and the lower bound of its 95% CIs are more significant than 0, it indicates that the ASR is expected to increase. Conversely, when both the EAPC and the upper bound of its 95% confidence interval are less than 0, it indicates that the ASR is expected to decrease (23).

Additionally, this study employs the fold change to reflect variation in incidence and death cases in 2021 compared with the corresponding baseline year.


Fold change=(2021data−baseline data)/baseline data.

Finally, the ratio is used to reflect the proportion and severity of incidence and death cases of MDR-TB, XDR-TB, HIV/AIDS-MDR-TB, and HIV/AIDS-XDR-TB globally and across the five SDI regions.


$${\text{Ratio}}_{\mathrm{MDR}-\mathrm{TB}}=\frac{\mathrm{MDR}-\mathrm{TB}\;}{\mathrm{TB}\;(\mathrm{HIV}-\text{negative})}$$

$${\text{Ratio}}_{\mathrm{XDR}-\mathrm{TB}}=\frac{\mathrm{XDR}-\mathrm{TB}\;}{\mathrm{TB}\;(\mathrm{HIV}-\text{negative})\;}$$

$${\text{Ratio}}_{\mathrm{HIV}/\text{AIDS}-\mathrm{MDR}-\mathrm{TB}}=\frac{\mathrm{HIV}/\text{AIDS}-\mathrm{MDR}-\mathrm{TB}\;}{\mathrm{HIV}/\text{AIDS}-\mathrm{TB}\;}$$

$${\text{Ratio}}_{\mathrm{HIV}/\text{AIDS}-\mathrm{XDR}-\mathrm{TB}}=\frac{\mathrm{HIV}/\text{AIDS}-\mathrm{XDR}-\mathrm{TB}\;\;}{\mathrm{HIV}/\text{AIDS}-\mathrm{TB}\;\;}$$

In this study, data organization and chart creation were performed using R software (version 4.3.1), with the ggplot2 package for visualization. Final data organization was completed in Excel, followed by editing in Adobe Illustrator (CS5) and Adobe Photoshop (version 25.3.1).

## Results

### Global level

A significant increase in the global burden of MDR-TB, XDR-TB, HIV/AIDS-MDR-TB, and HIV/AIDS-XDR-TB incidence and death has been reported from 1990 to 2021. Notably, the most substantial rise was observed in HIV/AIDS-XDR-TB, while MDR-TB experienced the smallest increase. For instance, the death cases of HIV/AIDS-XDR-TB increased from 9.78 (4.21 to 19.43) in 1993 to 840 (385.44 to1491.72) in 2021, representing an 84.89-fold increase. The EAPC in ASIR and ASDR for HIV/AIDS-XDR-TB was the highest, recorded at 9.96 (6.82 to13.19) and 10 (6.26 to 13.86), respectively. In contrast, the death cases of MDR-TB increased from 16,062.84 (5,895.34 to 38,846.4) in 1990 to 106,817.98 (41,611.7 to 211,853.75) in 2021, marking a 5.65-fold increase, with the EAPC of ASIR and ASDR being the lowest, at 2.05 (0.64 to 3.48) and 1.18 (−0.37 to 2.75), respectively. In 2021, the highest ASIR and ASDR for global DR-TB were observed in MDR-TB, with 5.4161 (3.1676 to 9.3429) and 1.2791 (0.4992 to 2.5331) per 100,000 population, respectively (Table 1; Supplementary Figure S1). Over the past three decades, the ratios of incidence and death from global MDR-TB, XDR-TB, HIV/AIDS-MDR-TB, and HIV/AIDS-XDR-TB have exhibited similar trends. Specifically, the ratios of MDR-TB and HIV/AIDS-MDR-TB incidence and death increased rapidly before 2008 and then stabilized. For example, the death ratio of MDR-TB rose from 0.9% in 1990 to 8.5% in 2008. In contrast, the ratios of XDR-TB and HIV/AIDS-XDR-TB incidence and death remained relatively stable, showing only a slight upward trend. This suggests that the global burden of DR-TB is increasing, with significant variations among these different types of DR-TB, posing substantial challenges worldwide (Table 1; Figure 1; Supplementary Figures S1, S2).

**Table 1:** The global incidence and deaths of MDR-TB, XDR-TB, HIV/AIDS-MDR-TB, and HIV/AIDS-XDR-TB cases and ASR at baseline and in 2021, and the trends from baseline to 2021.

	Number_baseline	Number_2021	Fold change	ASR_baseline	ASR_2021	EAPC
MDR-TB						
Incidence	51953.85 (24068.15,117145.74)	443679.91 (259195.8,766545.37)	7.54	1.0642 (0.498,2.3837)	5.4161 (3.1676,9.3429)	2.05 (0.64,3.48)
Deaths	16062.84 (5895.34,38846.4)	106817.98 (41611.7,211853.75)	5.65	0.3707 (0.1356,0.8973)	1.2791 (0.4992,2.5331)	1.18 (−0.37,2.75)
XDR-TB						
Incidence	999.21 (575.41,2170.86)	24036.08 (17144.48,34586.57)	23.06	0.0195 (0.0113,0.0423)	0.2905 (0.2083,0.4182)	7.24 (5.13,9.4)
Deaths	259.12 (117.04,562.06)	7946.44 (3326.48,14859.11)	29.67	0.0057 (0.0026,0.0123)	0.0943 (0.0395,0.1764)	5.97 (3.45,8.56)
HIV/AIDS-MDR-TB						
Incidence	1871.61 (1186.94,3024.32)	45589.4 (31326.35,66723.02)	23.36	0.0359 (0.0229,0.0575)	0.5546 (0.3811,0.8115)	4.71 (2.03,7.47)
Deaths	699.36 (267.43,1449.36)	17457.58 (7574.38,32229.49)	23.96	0.0137 (0.0052,0.0281)	0.2127 (0.0925,0.3901)	4.78 (1.49,8.17)
HIV/AIDS-XDR-TB						
Incidence	30.45 (18.85,59.94)	1606.42 (1163.94,2182.83)	51.76	0.0006 (0.0003,0.0011)	0.0194 (0.0141,0.0264)	9.96 (6.82,13.19)
Deaths	9.78 (4.21,19.43)	840 (385.44,1491.72)	84.89	0.0002 (0.0001,0.0004)	0.0101 (0.0047,0.018)	10 (6.26,13.86)

ASR, age-standardized rate; MDR-TB, multidrug-resistant tuberculosis; XDR-TB, extensively drug-resistant tuberculosis; EAPC, estimated annual percentage change.

**Figure 1:**
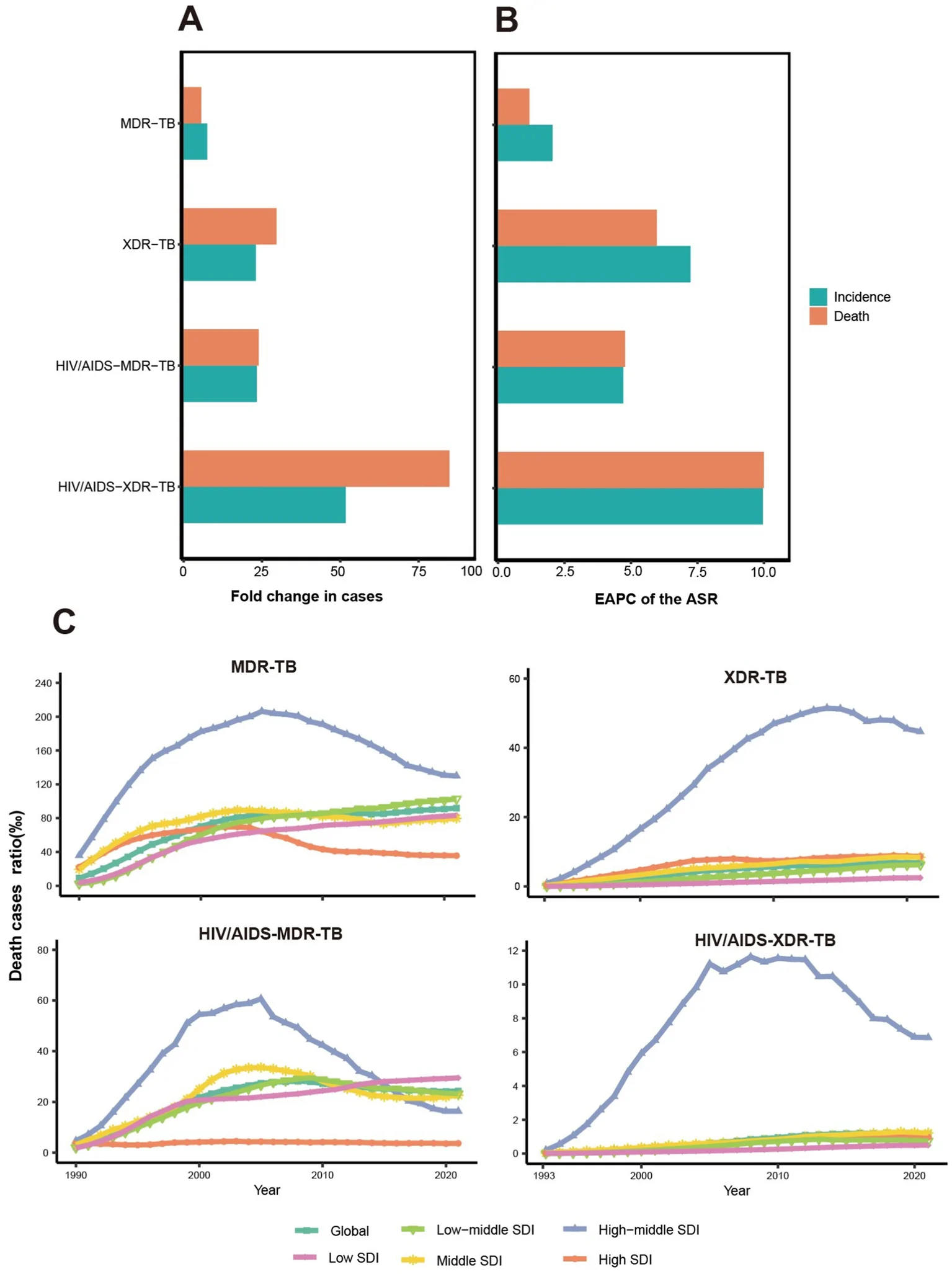
Temporal trend of MDR-TB, XDR-TB, HIV/AIDS-MDR-TB, and HIV/AIDS-XDR-TB globally and in 5 territories. **(A)** Fold change in cases of incidence and death globally from baseline to 2021. **(B)** The EAPC of ASIR and ASDR globally from baseline to 2021. **(C)** The death ratio of MDR-TB, XDR-TB, HIV/AIDS-MDR-TB, and HIV/AIDS-MDR-TB from baseline to 2021 globally and in 5 territories. MDR-TB, multidrug-resistant tuberculosis; XDR-TB, extensively drug-resistant tuberculosis; ASIR, age-standardized incidence rate; ASDR, age-standardized death rate; EAPC, estimated annual percentage change.

### SDI regional level

In 2021, the number of deaths from MDR-TB in the Low-middle SDI region reached 51,909.62 (16,133.61 to 115,997.03), accounting for 48.6% of the global total of MDR-TB deaths. Along with a gradual improvement of SDI, the ASDR for MDR-TB gradually decreases, falling from 5.0894 per 100,000 population (2.0547 to10.4449) in the Low SDI to 0.0234 (0.0094 to 0.0505) per 100,000 population in the High SDI. At the same time, the ASDR for MDR-TB shows an increasing trend in the Low SDI and Low-middle SDI regions over the past 32 years, with EAPC of 3.61 (1.57 to 5.68) and 4.8 (2.29 to 7.37), respectively. This indicates the burden of MDR-TB is more severe in developing regions. In contrast, the ASDR in the middle SDI exhibits a decreasing trend (EAPC = −2.51; 95% CI: −3.65 to −1.36), although this decline is slower compared to the High-middle SDI and the High SDI region. In contrast, while the burden of incidence and death from XDR-TB and HIV/AIDS-XDR-TB is substantial across Low to High-middle SDI regions, it is most significant in the High-middle SDI region, where the situation continues to worsen. For example, in 2021, the number of deaths from HIV/AIDS-XDR-TB in the High-middle SDI reached a peak of 285.87 (142.68 to 480.64), accounting for approximately 1/3 of the global HIV/AIDS-XDR-TB death. The ASIR and ASDR for HIV/AIDS-XDR-TB also reached their highest in the High-middle SDI, at 0.0523 (0.0324 to 0.0785) and 0.0188 (0.0094 to 0.0316) per 100,000 population, respectively. Over the past 32 years, there has been a significant increase in both the ASIR and ASDR for HIV/AIDS-XDR-TB, with EAPC of 11.36 (8.28 to 14.53) and 10.08 (6.46 to 13.82), respectively (Table 2; Figure 2; Supplementary Table S3; Supplementary Figures S2, S3).

**Table 2:** The global and regional death cases and ASDR of MDR-TB, XDR-TB, HIV/AIDS-MDR-TB, and HIV/AIDS-XDR-TB in 2021, and the trends from baseline to 2021.

	Death number_2021	Fold change	ASDR_2021	EAPC
MDR-TB				
Global	106817.98 (41611.7,211853.75)	5.65	1.2791 (0.4992,2.5331)	1.18 (−0.37,2.75)
Sex				
Both	106817.98 (41611.7,211853.75)	5.65	1.2791 (0.4992,2.5331)	1.18 (−0.37,2.75)
Male	66092.21 (26372.1,130949.47)	5.79	1.6485 (0.659,3.2634)	1.03 (−0.56,2.64)
Female	40725.76 (15495.44,81244.45)	5.44	0.9506 (0.3664,1.8795)	1.33 (−0.15,2.83)
Age				
<5 years	4545.95 (1891.74,9117.07)	1.55	0.6907 (0.2874,1.3852)	0.36 (−1.15,1.9)
5–14 years	1341.46 (566.51,2617.71)	2.99	0.0991 (0.0418,0.1934)	1.07 (−0.6,2.76)
15–49 years	33284.81 (13024.72,67070.34)	6.88	0.843 (0.3299,1.6986)	2.44 (0.67,4.25)
50–74 years	47154.52 (18138.25,94148.16)	5.65	2.8711 (1.1044,5.7324)	0.85 (−0.69,2.41)
75 + years	20491.24 (7697.32,40929.56)	6.79	7.102 (2.6678,14.1856)	0.8 (−0.57,2.19)
SDI regions				
Low SDI	29312.63 (11965.02,59516.62)	16.18	5.0894 (2.0547,10.4449)	3.61 (1.57,5.68)
Low-middle SDI	51909.62 (16133.61,115997.03)	29.36	3.4486 (1.068,7.702)	4.8 (2.29,7.37)
Middle SDI	20262.83 (7337.72,41058.75)	1.41	0.7838 (0.2844,1.5823)	−2.51 (−3.65,-1.36)
High-middle SDI	4831.87 (2645.3,8293)	0.38	0.2693 (0.1477,0.4615)	−3.67 (−5.35,-1.95)
High SDI	470.1 (184.03,1026.81)	−0.35	0.0234 (0.0094,0.0505)	−6.13 (−7.13,-5.12)
XDR-TB				
Global	7946.44 (3326.48,14859.11)	29.67	0.0943 (0.0395,0.1764)	5.97 (3.45,8.56)
Sex				
Both	7946.44 (3326.48,14859.11)	29.67	0.0943 (0.0395,0.1764)	5.97 (3.45,8.56)
Male	5268.99 (2306.97,10047.04)	30.26	0.1305 (0.0568,0.2499)	5.82 (3.23,8.48)
Female	2677.45 (1024.38,5133.07)	28.56	0.0616 (0.0237,0.1181)	6.19 (3.85,8.6)
Age				
<5 years	182.68 (78.82,361.65)	8.42	0.0278 (0.012,0.0549)	4.34 (2.22,6.5)
5–14 years	70.21 (27.92,139.44)	14.10	0.0052 (0.0021,0.0103)	5 (2.64,7.41)
15–49 years	2442.17 (1074.75,4598.74)	32.75	0.0618 (0.0272,0.1165)	6.9 (3.98,9.91)
50–74 years	3711.23 (1561.66,7030.69)	29.92	0.226 (0.0951,0.4281)	5.78 (3.31,8.31)
75 + years	1540.15 (575.29,3032.33)	35.09	0.5338 (0.1994,1.051)	5.83 (3.68,8.04)
SDI regions				
Low SDI	874.03 (293.16,1936.16)	136.43	0.1572 (0.0502,0.3598)	10.88 (7.8,14.04)
Low-middle SDI	3153.42 (1011.39,6914.67)	116.40	0.21 (0.0676,0.4592)	9.78 (6.71,12.94)
Middle SDI	2138.13 (834.8,3992.13)	15.64	0.0829 (0.0326,0.1556)	3.5 (1.46,5.58)
High-middle SDI	1662.52 (893.99,2774.42)	18.52	0.093 (0.0501,0.1548)	4.53 (1.5,7.65)
High SDI	114.2 (44.99,240.79)	8.45	0.0055 (0.0022,0.0115)	1.52 (−0.49,3.58)
HIV/AIDS-MDR-TB				
Global	17457.58 (7574.38,32229.49)	23.96	0.2127 (0.0925,0.3901)	4.78 (1.49,8.17)
Sex				
Both	17457.58 (7574.38,32229.49)	23.96	0.2127 (0.0925,0.3901)	4.78 (1.49,8.17)
Male	8313.01 (3674.04,15181.08)	20.61	0.2004 (0.0885,0.3663)	4.63 (1.5,7.87)
Female	9144.57 (3964,17117.32)	28.06	0.2258 (0.0981,0.4218)	4.93 (1.49,8.48)
Age				
<5 years	510.01 (197.2,1006.73)	4.41	0.0775 (0.03,0.153)	0.84 (−2.64,4.44)
5–14 years	535.89 (226.37,1011.98)	103.26	0.0396 (0.0167,0.0748)	11.58 (7.57,15.75)
15–49 years	12499.13 (5542.76,22856.89)	24.55	0.3165 (0.1404,0.5789)	5.25 (1.94,8.66)
50–74 years	3771.28 (1672.16,7271.15)	34.71	0.2296 (0.1018,0.4427)	4.82 (1.59,8.16)
75 + years	141.27 (60.58,270.31)	26.59	0.049 (0.021,0.0937)	2.84 (−0.29,6.07)
SDI regions				
Low SDI	6542.06 (2633.61,12881.95)	20.43	0.7318 (0.2978,1.4358)	2.7 (−0.27,5.75)
Low-middle SDI	5303.9 (2160.05,10458.59)	41.82	0.2859 (0.1172,0.5613)	5.56 (1.8,9.45)
Middle SDI	4883.74 (1662.98,10664.57)	56.11	0.1797 (0.0611,0.3932)	6.83 (2.67,11.16)
High-middle SDI	682.16 (343.19,1129.9)	11.72	0.0446 (0.0225,0.0736)	3.41 (0.57,6.33)
High SDI	37.88 (14.97,80.75)	−0.71	0.0028 (0.0011,0.006)	−6.02 (−6.42,-5.62)
HIV/AIDS-XDR-TB				
Global	840 (385.44,1491.72)	84.89	0.0101 (0.0047,0.018)	10 (6.26,13.86)
Sex				
Both	840 (385.44,1491.72)	84.89	0.0101 (0.0047,0.018)	10 (6.26,13.86)
Male	507.45 (234.55,893.75)	72.12	0.0122 (0.0056,0.0214)	9.36 (5.72,13.13)
Female	332.55 (151.17,580.89)	116.10	0.0081 (0.0037,0.0142)	11.19 (7.26,15.27)
Age				
<5 years	12.67 (5.68,23.09)	24.34	0.0019 (9e-04,0.0035)	6.32 (2.36,10.44)
5–14 years	11.57 (5,21.02)	88.00	0.0009 (0.0004,0.0016)	12.41 (8.57,16.39)
15–49 years	624.92 (291.93,1101.48)	82.77	0.0158 (0.0074,0.0279)	10.4 (6.58,14.35)
50–74 years	183.93 (81.32,340.69)	114.68	0.0112 (0.005,0.0207)	10.07 (6.63,13.63)
75 + years	6.91 (2.53,16.12)	61.82	0.0024 (0.0009,0.0056)	7.07 (3.99,10.24)
SDI regions				
Low SDI	109.2 (42.36,219.23)	97.38	0.0123 (0.0049,0.0248)	8.7 (5.22,12.29)
Low-middle SDI	171.79 (67.99,319.63)	225.04	0.0093 (0.0037,0.0174)	12.08 (7.12,17.26)
Middle SDI	262.6 (92.37,537.72)	116.76	0.0096 (0.0034,0.0197)	10.45 (6.57,14.47)
High-middle SDI	285.87 (142.68,480.64)	70.11	0.0188 (0.0094,0.0316)	10.08 (6.46,13.82)
High SDI	9.95 (3.8,20.24)	5.07	0.0007 (0.0003,0.0015)	3.09 (1.73,4.46)

ASR, age-standardized rate; MDR-TB, multidrug-resistant tuberculosis; XDR-TB, extensively drug-resistant tuberculosis; SDI, Social Demographic Index, EAPC, estimated annual percentage change.

**Figure 2:**
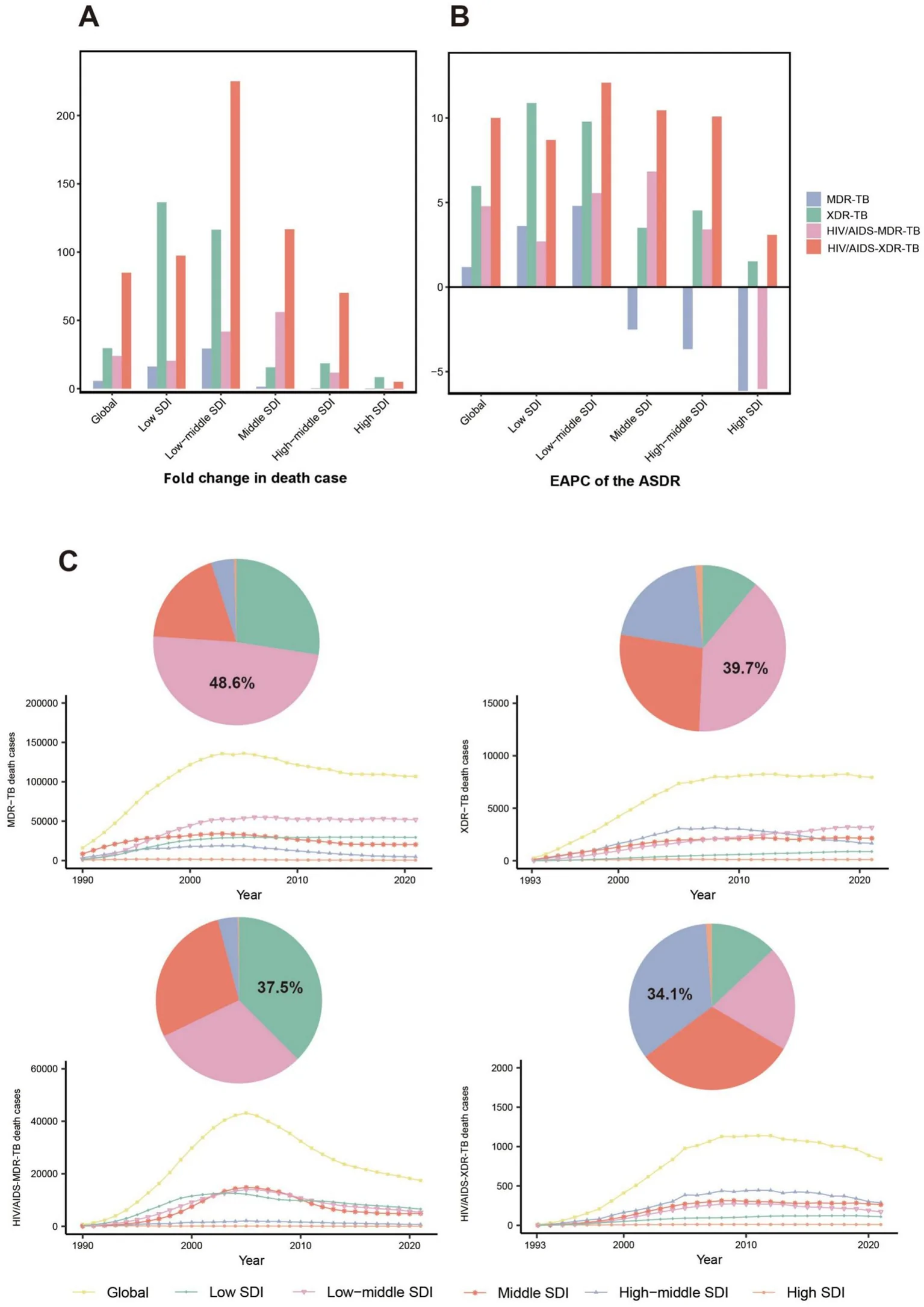
Temporal trend of drug-resistant globally and in 5 territories. **(A)** Fold change in death cases from baseline to 2021. **(B)** The EAPC of ASDR from baseline to 2021. **(C)** The death cases of MDR-TB, XDR-TB, HIV/AIDS-MDR-TB, and HIV/AIDS-XDR-TB from baseline to 2021(line chart) and the percentage distribution of global MDR-TB, XDR-TB, HIV/AIDS-MDR-TB, and HIV/AIDS-XDR-TB death cases across the 5 SDI regions in 2021 (pie chart). MDR-TB, multidrug-resistant tuberculosis; XDR-TB, extensively drug-resistant tuberculosis; ASIR, age-standardized incidence rate; EAPC, estimated annual percentage change; SDI, Social Demographic Index.

Alarmingly, the ratio of incidence and death of MDR-TB, XDR-TB, HIV/AIDS-MDR-TB, and HIV/AIDS-XDR-TB in the High-middle SDI region has been the highest over the past three decades. For instance, the ratio of deaths of MDR-TB in the High-middle SDI reached as high as 20.6%, compared to only 7% in the High SDI, which is just 1/3 of the High-middle SDI. This indicates that the burden of these types of DR-TB in the High-middle SDI is particularly severe and requires urgent attention and intervention (Figure 1C; Supplementary Figure S2).

### Regions level

A significant increase in the burden of MDR-TB, XDR-TB, HIV/AIDS-MDR-TB, and HIV/AIDS-XDR-TB incidence and death was observed in the majority of regions over the past 32 years. However, only a few regions, specifically High-income North America, East Asia, Caribbean and Western Europe, have exhibited a decrease (Figure 3; Supplementary Tables S4, S5; Supplementary Figure S4). For instance, High-income North America demonstrated the most significant declining trends in the EAPC of ASIR and ASDR for HIV/AIDS-MDR-TB, with −9.24 (−10.21 to −8.26) and −10.74 (−12.12 to −9.35), respectively. In 2021, the number of MDR-TB incidences and deaths in High-income North America was 145.86 (60.36 to 350.94) and 26.8 (7.62 to 76.43), respectively, showing a fold change of −0.62 and −0.83 over the past 32 years. This might be attributed to the region’s early screening, effective treatment, vaccination, and improved public health measures. In contrast, the most pronounced increases in the ASIR and ASDR for these types of DR-TB were observed in Oceania and South Asia.

**Figure 3:**
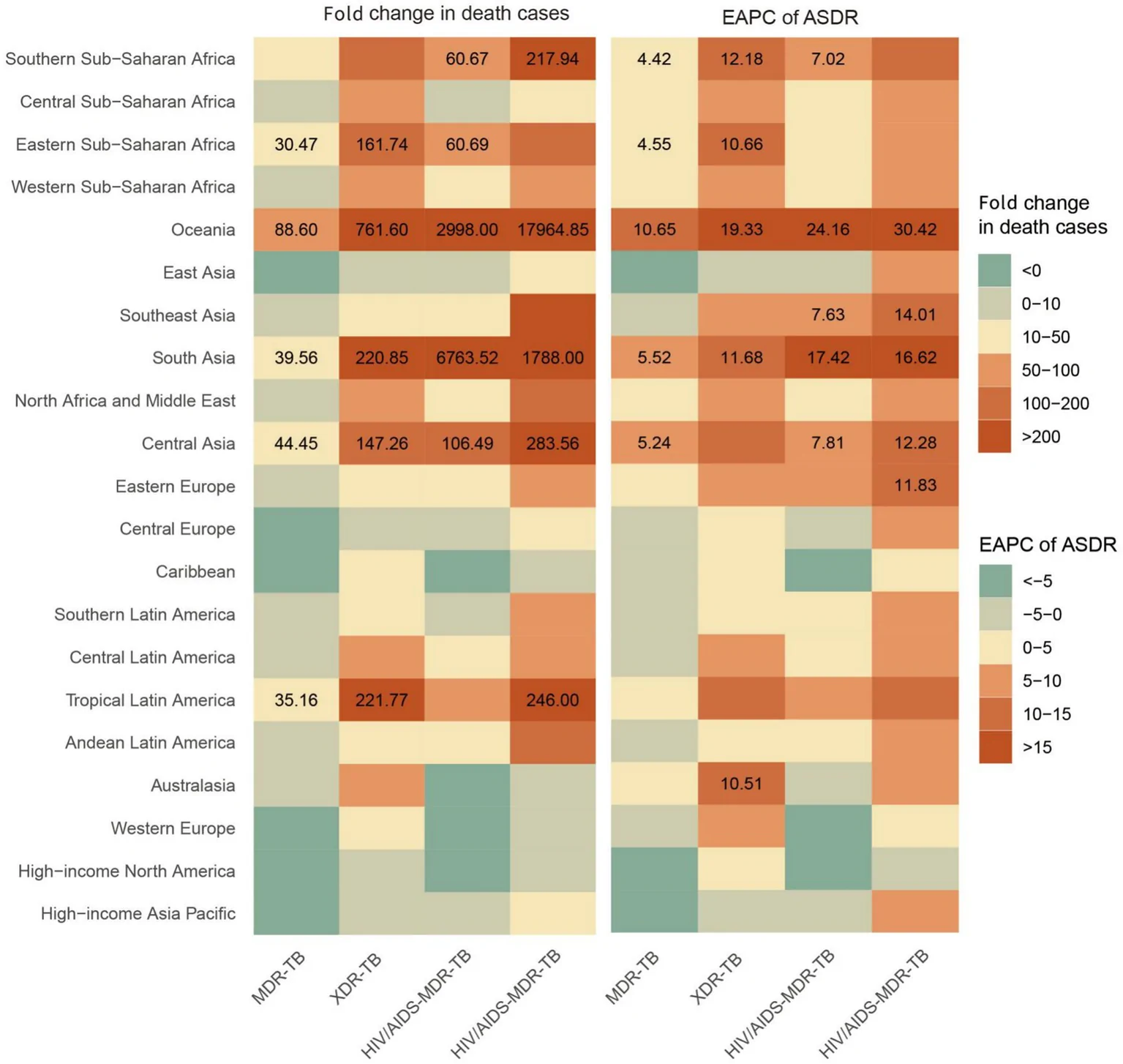
Temporal trend of MDR-TB, XDR-TB, HIV/AIDS-MDR-TB, and HIV/AIDS-XDR-TB in regions. **(A)** Fold change in death cases from baseline to 2021. **(B)** The EAPC of ASDR from baseline to 2021. MDR-TB, multidrug-resistant tuberculosis; XDR-TB, extensively drug-resistant tuberculosis; ASDR, age-standardized death rate; EAPC, estimated annual percentage change.

### National level

Over the past 32 years, the ASIR and ASDR for MDR-TB, XDR-TB, HIV/AIDS-MDR-TB, and HIV/AIDS-XDR-TB have shown an increasing trend in most countries, except for the ASDR of MDR-TB (Figure 4; Supplementary Tables S6–S13; Supplementary Figure S5). The countries with the most significant increases in ASDR for MDR-TB, XDR-TB, HIV/AIDS-MDR-TB, and HIV/AIDS-XDR-TB are Lesotho, Papua New Guinea, Pakistan and Pakistan, respectively, with EAPCs of 12.6 (9.67 to 15.61), 19.84 (17.16 to 22.57), 46.09 (39.33 to 53.17), and 50.9 (43.59 to 58.58). Conversely, the most significant declines were exhibited in Slovenia. In 2021, India reported high cases of MDR-TB, XDR-TB, HIV/AIDS-MDR-TB, and HIV/AIDS-XDR-TB. Significantly, the death cases for MDR-TB and XDR-TB were the highest, with 53,333.12 (10,384.24 to 133,519.76) and 2,815.29 (502.44 to 7,178.1), respectively, accounting for approximately 1/2 and 1/3 of global deaths from these diseases. Furthermore, the ASIR and ASDR for these types of DR-TB in India are also on the rise, indicating that the country is facing a severe challenge with these types of DR-TB. In contrast to India, although China reports higher cases of incidence and death from MDR-TB, XDR-TB, HIV/AIDS-MDR-TB, and HIV/AIDS-XDR-TB, while the ASIR and ASDR for these types of DR-TB in China are relatively low and have demonstrated a declining trend over the past 32 years (excluding HIV/AIDS-XDR-TB). For instance, the ASDR for MDR-TB is only 0.1469 (0.0246 to 0.4272) per 100,000 population, with EAPC of −10.03 (−11.23 to −8.82).

**Figure 4:**
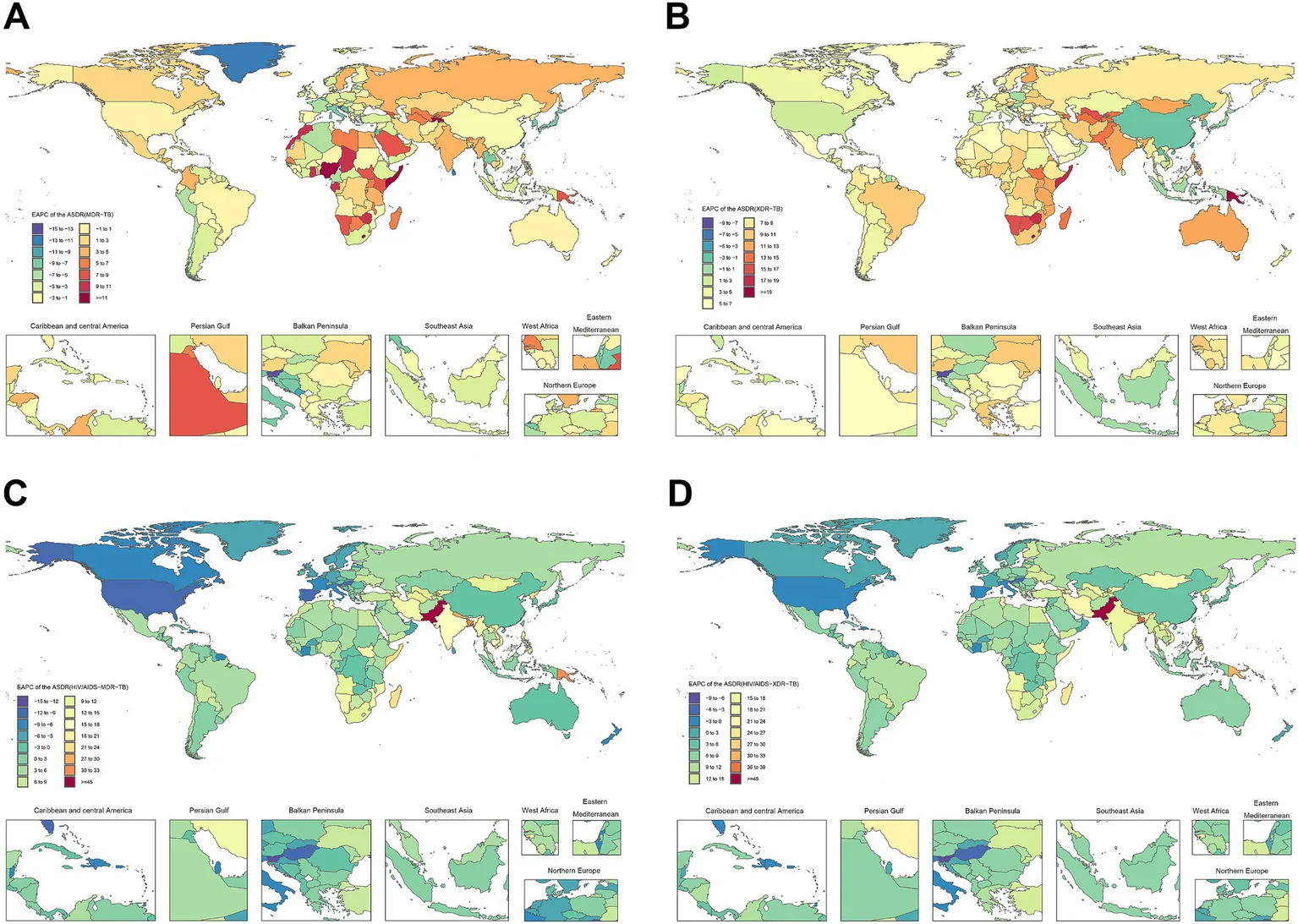
Temporal trend of the EAPC of ASDR for MDR-TB **(A)**, XDR-TB **(B)**, HIV/AIDS-MDR-TB **(C)**, and HIV/AIDS-XDR-TB **(D)** from baseline to 2021 across 204 countries. MDR-TB, multidrug-resistant tuberculosis; XDR-TB, extensively drug-resistant tuberculosis; ASDR, age-standardized death rate; EAPC, estimated annual percentage change.

### Sex level

From 1990 to 2021, a significant increase in the global burden of MDR-TB, XDR-TB, HIV/AIDS-MDR-TB, and HIV/AIDS-XDR-TB incidence and death have been reported for both males and females, with women exhibiting greater increases in ASIR and ASDR than men. However, in 2021, men exhibited a higher burden for these types of DR-TB compared to women, excluding HIV/AIDS-MDR-TB. This indicates that, excluding HIV/AIDS-MDR-TB, the global burden of these types of DR-TB is higher in men than in women, yet the trend of increase among women surpasses that of men. Consequently, the gap in the burden between genders is expected to narrow gradually. However, the burden of incidence and death for HIV/AIDS-MDR-TB is higher for women in 2021, and the increase trends among women also exceed that of men over the past three decades, indicating that the gender disparity is likely to worsen. For example, the EAPC for ASIR among women is 4.97 (2.16 to 7.86), while for men, it is 4.44 (1.89 to 7.06). In 2021, the ASIR for women was 0.5886 (0.4054 to 0.8723) per 100,000 population, compared to 0.5223 (0.3611 to 0.7612) for men (Table 2; Supplementary Table S3; Supplementary Figures S6, S7).

### Age level

From 1990 to 2021, an increasing trend in the burden of MDR-TB, XDR-TB, HIV/AIDS-MDR-TB, and HIV/AIDS-XDR-TB has been observed across all age groups and both sexes. The incidence and death burden of MDR-TB and XDR-TB increased, peaking in the 15–49 age group. For example, the EAPC for the ASIR and ASDR of MDR-TB in the 15–49 age group are 2.85 (1.4 to 4.33,) and 2.44 (0.67 to 4.25), respectively. In 2021, the ASIR and ASDR for both MDR-TB and XDR-TB demonstrated a “√-shaped” trend with age, initially declining and then rising, peaking at age 75 and older, while the lowest ASIR and ASDR were observed in the 5–14 age group. For instance, the ASDR for MDR-TB in the 5–14 age group is 0.0991 (0.0418 to 0.1934) per 100,000 population, whereas in the 75 and older group, it rises to 7.102 (2.6678 to 14.1856) per 100,000 population, approximately 70-fold that of the 5–14 age group (Table 2; Figure 5; Supplementary Tables S3, S14-S15; Supplementary Figures S8–S11).

**Figure 5:**
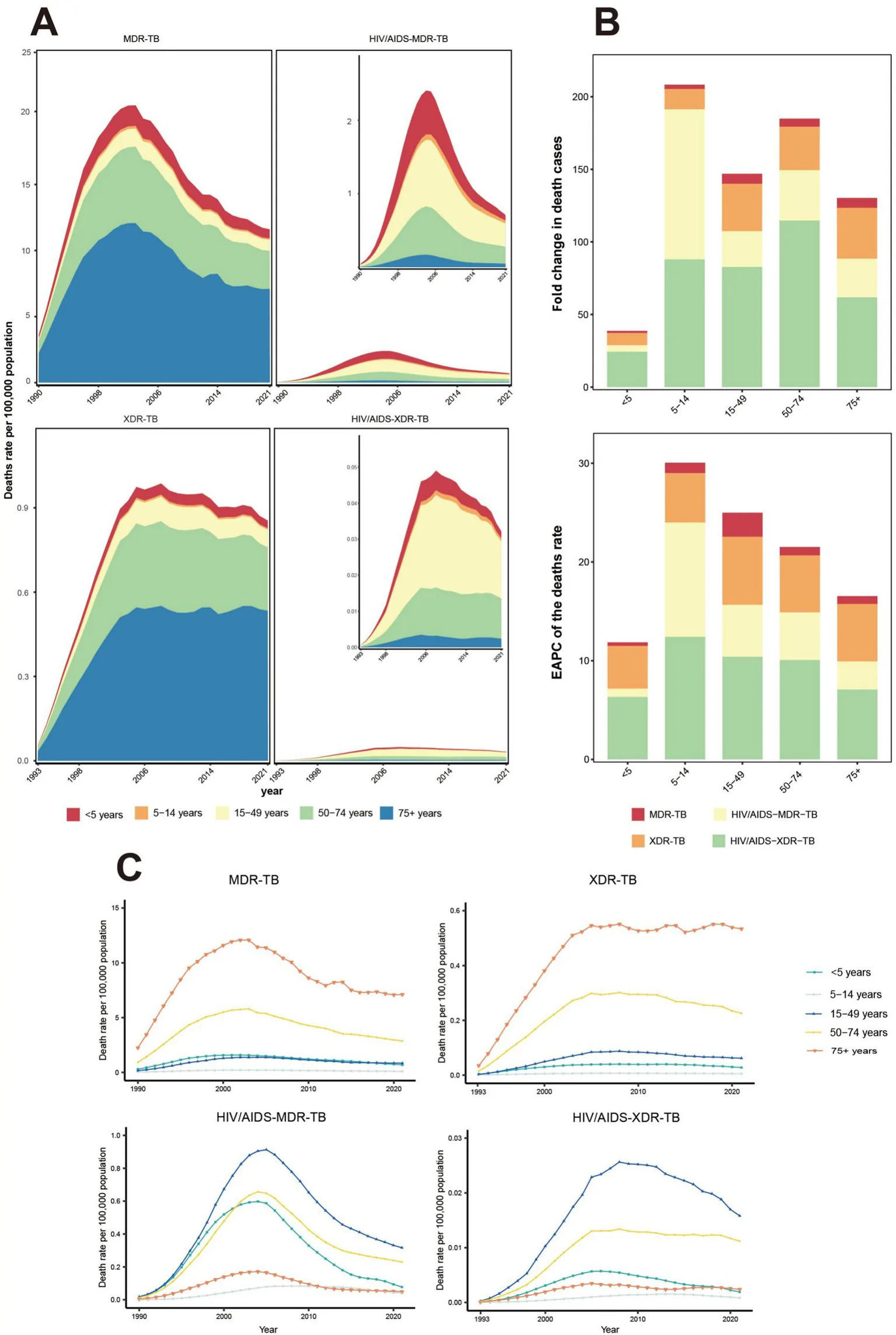
Global temporal trend of MDR-TB, XDR-TB, HIV/AIDS-MDR-TB, and HIV/AIDS-XDR-TB by age pattern. **(A)** The global ASDR in 5 age groups from baseline to 2021. **(B)** Fold change in death cases across 5 age groups globally from baseline to 2021. **(C)** The EAPC of ASDR for 5 age groups globally from baseline to 2021. MDR-TB, multidrug-resistant tuberculosis; XDR-TB, extensively drug-resistant tuberculosis; ASDR, age-standardized death rate; EAPC, estimated annual percentage change.

Distinctly, from 1990 to 2021, the incidence and death burden of HIV/AIDS-MDR-TB and HIV/AIDS-XDR-TB show the highest increase in the 5–14 age group (Table 2; Figure 5B; Supplementary Table S3; Supplementary Figure S8). For instance, the death cases with HIV/AIDS-MDR-TB in this age group has increased a staggering 103.26 times. The ASDR exhibits EAPC of 11.58 (7.57 to 15.75). In 2021, both the ASIR and ASDR for both HIV/AIDS-MDR-TB and HIV/AIDS-XDR-TB peaked in the 15–49 age group. For example, the ASDR for HIV/AIDS-MDR-TB in this group is 0.3165 (0.1404 to 0.5789) per 100,000 population. This indicates that these types of DR-TB prevention and control strategies should focus more on specific age groups to enhance their effectiveness.

### Risk factors

The proportion of deaths caused by MDR-TB, XDR-TB, HIV/AIDS-MDR-TB, and HIV/AIDS-XDR-TB attributable to individual risk factors varied across the different GBD regions. Globally, the major contributors to deaths from MDR-TB and XDR-TB were smoking, HFPG, high alcohol use, and high body mass index (high BMI). The high-middle SDI region exhibited the highest contributions of smoking and high alcohol use to deaths from both MDR-TB and XDR-TB, significantly surpassing those in other SDI regions. The contributions of smoking and high alcohol use to death from MDR-TB were 30.5% and 21.8%, respectively, while for XDR-TB, the contributions were 33.4% and 23.6%. Significant gender differences in the proportions of MDR-TB, XDR-TB, HIV/AIDS-MDR-TB, and HIV/AIDS-XDR-TB deaths attributable to risk factors were observed globally. For women, HFPG and high BMI are the primary contributors to death. In contrast, for men, the main risk factors for death are smoking, high alcohol use, and HFPG (Figure 6; Supplementary Figures S12, S13).

**Figure 6:**
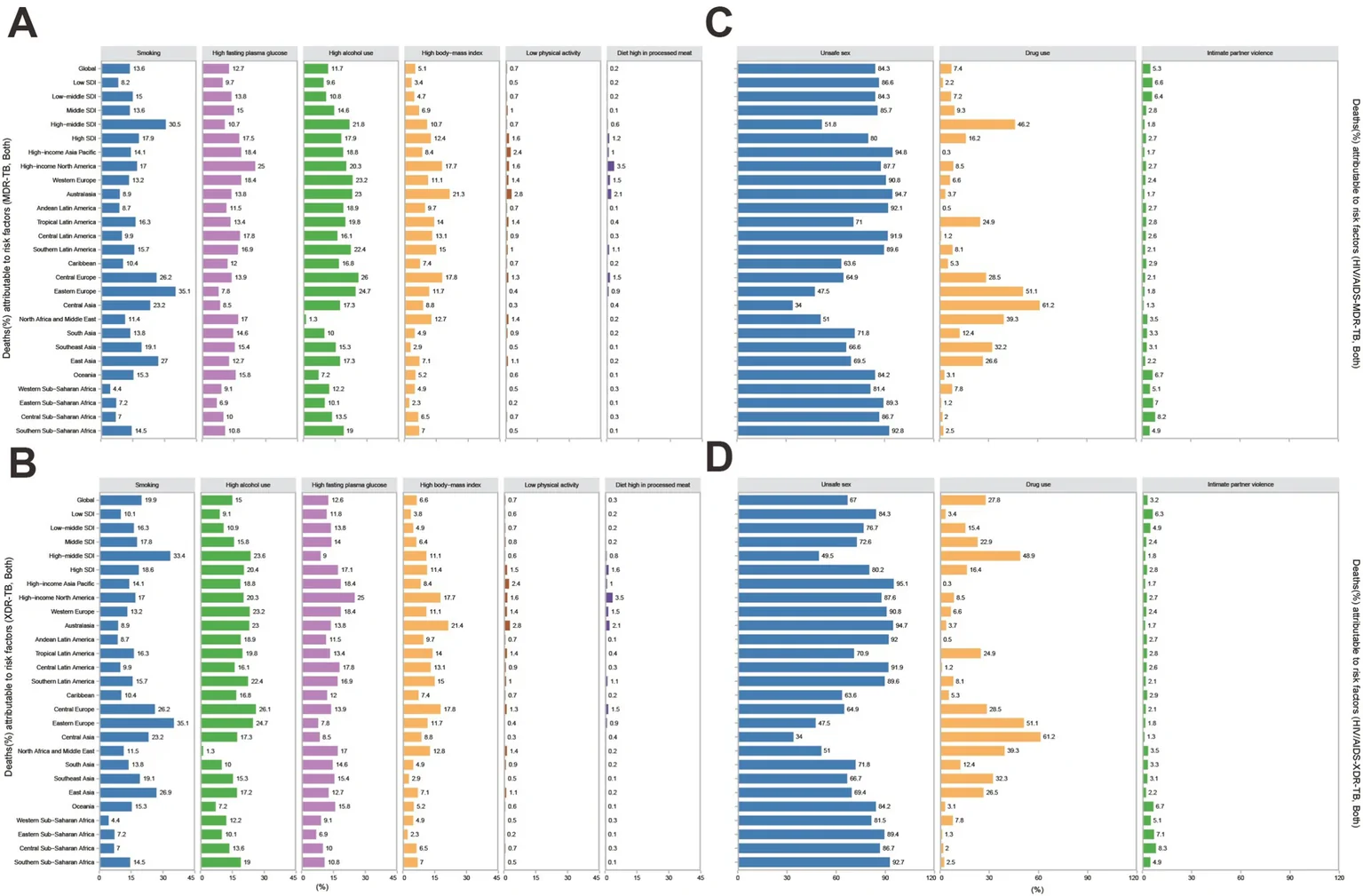
Percentage of death due to MDR-TB **(A)**, XDR-TB **(B)**, HIV/AIDS-MDR-TB**(C)**, and HIV/AIDS-XDR-TB **(D)** attributable to significant risk factors globally, across 5 territories, and in 21 GBD regions in 2021. MDR-TB, multidrug-resistant tuberculosis; XDR-TB, extensively drug-resistant tuberculosis; GBD, Global Burden of Disease.

## Discussion

The study findings indicate that there has been a significant increase in the global burden of MDR-TB, XDR-TB, HIV/AIDS-MDR-TB, and HIV/AIDS-XDR-TB incidence and death over the past three decades, a trend that contrasts with the global TB results (excluding XDR-TB) monitored by the WHO (1). Notably, the burden of HIV/AIDS-XDR-TB has shown the most significant growth. This data can be viewed as a complement to WHO statistics, highlighting the extreme threat and severity of XDR-TB, whether or not associated with HIV/AIDS, particularly in comparison to MDR-TB. The incidence and death ratio of MDR-TB and HIV/AIDS-MDR-TB increased rapidly prior to 2008 and subsequently stabilized. This trend is associated with the global tuberculosis control strategies proposed by the WHO in the early 21st century, which encouraged countries to establish systems for the prevention and control of DR-TB and enhance the monitoring, testing, and treatment of MDR-TB worldwide. These measures have effectively managed and controlled the incidence and death rates of MDR-TB (24, 25). It should be noted that in 2021, the ASIR and ASDR of MDR-TB were the highest among DR-TB. On one hand, it highlights that the burden of MDR-TB is the most substantial among DR-TB. On the other hand, this may be attributed to significant advancements in the monitoring of DR-TB, which have predominantly focused on MDR-TB, such as the increasingly improved MDR-TB detection methods and the WHO’s special focus on MDR-TB in its TB report (1).

The burden of MDR-TB and HIV/AIDS-MDR-TB is more severe in developing regions. It is well established that TB incidence and death rates are closely associated with socioeconomic and development levels (26). On one hand, the increased detection coverage of MDR-TB has been accompanied by a rise in reported MDR-TB cases. Studies show that in low- and middle-income countries, the average patient delay, health system delay, and total diagnostic delay are as high as 31.7 days, 28.4 days, and 67.8 days, respectively, which escalates DR-TB transmission, deteriorates the condition, and increases mortality (27). On the other hand, the capacity for community management has been identified as a factor associated with the extent and magnitude of MDR-TB transmission. Studies have shown that the rise in incident and death numbers of MDR-TB is primarily due to sustained transmission between individuals (28). Approximately one-third of MDR-TB patients remain sputum smear-positive at discharge, further heightening the risk of community transmission (29). However, effective community management capacity is closely linked to SDI levels. These findings suggest that the significance of expanding detection coverage and refining community-based control strategies, especially within low-SDI regions, may contribute to curb the proliferation of MDR-TB. Further analysis reveals that in 2021, the number of deaths from MDR-TB in low-middle SDI regions was the highest, accounting for approximately 1/2 of the global total. This may associated with numerous obstacles to TB control in the region, including population growth, poverty, and an overstretched healthcare system (30, 31). Research also indicates that the burden of XDR-TB and HIV/AIDS-XDR-TB is significant across low- to high-middle SDI regions, with the heaviest burden observed in high-middle SDI regions, which is worsening. Regions exhibiting a high MDR rate tend to have a high XDR rate, which is consistent with the principle of drug resistance in biology. A high MDR rate may lead to a high XDR rate by increasing the use of second-line drugs (32). Substance abuse and HIV infection also can exacerbate this situation (33). Therefore, it is necessary to develop a One Health-based strategy to reduce the burden of DR-TB, which includes improving public health education, enhancing the use of resistance detection technologies, strengthening epidemiological studies, improving community control, accelerating drug research, promoting interdisciplinary cooperation, providing robust policy support, and fostering international collaboration to offer more effective and comprehensive DR-TB prevention and control methods.

There are significant disparities in the burden of MDR-TB, XDR-TB, HIV/AIDS-MDR-TB, and HIV/AIDS-XDR-TB incidents and deaths across different regions and countries globally. Overall, over the past 32 years, the burden of these types of DR-TB has exhibited an increasing trend in most regions. Notably, Oceania, South Asia, and Central Asia exhibit the most pronounced increases in ASIR and ASDR. This is consistent with the 2023 WHO Global TB Report, which indicates that Southeast Asia, Africa, and the Western Pacific regions are high TB burden areas, with countries like India, Pakistan, and Bangladesh being major contributors to the TB burden in these regions (4). In 2021, India recorded the highest number of MDR-TB and XDR-TB deaths, posing formidable challenges. This situation is closely tied to the economic burden of tuberculosis treatment, compounded by the country’s large population. In India, the costs of TB treatment are primarily borne by patients themselves, and the low universal health coverage index makes it difficult for residents in impoverished areas to access primary healthcare services (34). Therefore, India urgently needs to prioritize the allocation of healthcare resources, particularly in impoverished areas, by addressing hunger and implementing social protection schemes targeted at TB patients to confront this crisis effectively. Additionally, research indicates that the ASDR for XDR-TB is highest in Papua New Guinea, likely linked to the active migration flows and tourism that may facilitate the spread of DR-TB (35). Consequently, it is essential to emphasize the importance of pre-migration screening in countries with high levels of migrant mobility to effectively monitor and control the spread of infectious diseases.

Encouragingly, certain regions and countries are experiencing a decline in the burden of MDR-TB, XDR-TB, HIV/AIDS-MDR-TB, and HIV/AIDS-XDR-TB, particularly in high-income North America and East Asia. In high-income North America, a robust healthcare system facilitates effective and autonomous TB treatment, thereby reducing the incidence of drug-resistant strains (21). In East Asia, particularly in China, the burden of MDR-TB has been successfully reduced in recent years through effective social protection measures and an increasingly improved tuberculosis prevention and control service system. Furthermore, Slovenia has exhibited the most significant decrease in both ASIR and ASDR of DR-TB. Research indicates that due to Slovenia’s effective TB prevention and treatment initiative, the co-prevalence of MDR-TB is ≤1% (36). Overall, the experiences of these countries and regions demonstrate that the spread of DR-TB can be effectively curtailed through strong public health measures, comprehensive healthcare systems, and social protection policies.

Consistent with existing research, men globally experienced a significantly higher burden of MDR-TB, XDR-TB, HIV/AIDS-MDR-TB, and HIV/AIDS-XDR-TB incidence and death (excluding HIV/AIDS-XDR-TB) compared to women (1). This disparity can be attributed to the fact that TB is transmitted through social interactions in 80 to 90% of cases, with men predominantly occupying these transmission pathways (37). Additionally, men are more susceptible to TB due to factors such as smoking, alcohol abuse, higher BMI, and chronic work-related stress. In contrast, the burden of HIV/AIDS-MDR-TB is greater among women than men, and this gender gap may be further exacerbated. This may be related to women’s increased vulnerability to HIV/AIDS, which can be attributed to factors such as unsafe sex, intimate partner violence, violence against women, cultures that disempower women, and systematically inadequate sexual and reproductive health services (38, 39). Moreover, HIV serves as a significant risk factor for the progression of latent tuberculosis to active disease, further intensifying this disparity (39).

From an age-pattern perspective, individuals aged 15 to 49 exhibited a significant increase in the global burden of MDR-TB and XDR-TB. However, in 2021, the ASIR and ASDR for MDR-TB and XDR-TB were highest among individuals aged 75 and older. A history of prior tuberculosis treatment, treatment failure, and substance abuse can contribute to the development of drug resistance. Older patients often present with more comorbidities and greater overall disease severity, and a higher proportion of this population was not vaccinated with BCG at birth, which further exacerbates treatment-related challenges and increases the risks of resistance, disease recurrence and mortality (40). Additionally, the diagnosis and treatment of DR-TB in older patients are more likely to be delayed (41). The burden of HIV/AIDS-MDR-TB and HIV/AIDS-XDR-TB has shown the most significant increase among individuals aged 5 to 14. This finding warrants cautious interpretation given small case numbers, improved diagnostic practices, and substantial modeling uncertainty in GBD paediatric estimates. Consequently, in addition to effective prevention and control measures, the development of rapid and accurate diagnostic tools, along with shorter, more effective, and less toxic treatment regimens, is crucial for addressing DR-TB, particularly in pediatric and older adult populations.

In 2021, the primary global risk factors associated with deaths from MDR-TB and XDR-TB globally included smoking, HFPG, high alcohol consumption, and high BMI. Multiple studies have identified smoking and alcohol use as significant risk factors for DR-TB mortality. Therefore, effective tobacco and alcohol control measures will be the most effective long-term strategy for alleviating the burden of DR-TB, particularly among the most affected male populations and in high-middle SDI regions (14, 42, 43). In areas with high metabolic risks, such as those characterized by HFPG, the importance of routine bidirectional screening and comprehensive management should be emphasized, especially for the most affected female populations, as this may alleviate the burden of DR-TB (13). High BMI has surpassed alcohol use and smoking as the second leading contributor to DR-TB-related deaths among females, marking a notable shift over the 1990–2021 period. This trend aligns with the global rise in obesity, driven by urbanization, sedentary lifestyles, and shifts in dietary patterns. The association between high BMI and growing DR-TB burden presents a complex paradox (44). Potential explanations include obesity-related immune dysfunction, such as gut microbiota imbalance and abnormal cytokine responses, alongside the compounding effects of diabetes—both a consequence of obesity and a known factor that amplifies TB susceptibility and disease severity (45).

For HIV/AIDS-associated DR-TB, the main attributable risk factors identified in GBD 2021 were unsafe sex, drug use, and intimate partner violence. These variables are primarily upstream determinants of HIV acquisition rather than direct causal factors for DR-TB mortality; the causal pathway operates largely through HIV infection and its subsequent effects on immunosuppression, diagnostic delays, and treatment challenges. A recent GBD 2021-based analysis identified unsafe sex as the predominant risk factor for HIV/DR-TB co-infection in both genders, followed by drug use in males and intimate partner violence in females. Regionally, male drug use was more strongly associated in high-middle SDI regions, whereas female unsafe sex and intimate partner violence had greater impact in low SDI (46). Regions HIV co-infection has been independently associated with increased post-TB mortality among persons with DR-TB (47). Therefore, a focus on effective communication and preventive measures may contribute to reducing modifiable risk factors. However, these factors are general TB risk factors, and GBD 2021 does not include several established DR-TB drivers, such as previous treatment history, treatment interruption, poor adherence, incarceration, migration, and nosocomial transmission. Therefore, this risk factor analysis should be interpreted as a complementary assessment within the GBD framework, not a comprehensive causal analysis.

This study has several inherent limitations. First, the data primarily originate from disease reports and healthcare institutions, which may lead to incomplete or inaccurate estimates. Second, the data obtained from the GBD study rely heavily on statistical modeling methods, particularly in countries lacking original data, which may affect the accuracy of the results. Third, GBD 2021 used the pre-2021 WHO definition of XDR-TB, which was based on resistance to second-line injectable drugs. In January 2021, WHO revised this definition to include resistance to bedaquiline or linezolid and introduced pre-XDR-TB as a new category. This change limits comparability with WHO surveillance data and may underestimate the XDR-TB burden under the current definition. Fourth, GBD 2021 does not report pre-XDR-TB separately, so this intermediate phenotype could not be assessed. Fifth, we lacked strain-level and genotypic resistance data, and the GBD estimates do not capture resistance to bedaquiline or linezolid, which are now central to DR-TB treatment. Sixth, the GBD database does not contain treatment regimen, adherence, or programmatic/health-system variables. A more nuanced analysis of the programmatic drivers of DR-TB burden was therefore not possible. Finally, it is important to note the time lag inherent of GBD data. Therefore, it is recommended that other data sources and field surveys be integrated for a more accurate and comprehensive assessment.

## Conclusion

Over the past 32 years, the global burden of MDR-TB, XDR-TB, HIV/AIDS-MDR-TB, and HIV/AIDS-XDR-TB has significantly increased, with notable differences observed between regions and countries. The burden of MDR-TB and HIV/AIDS-MDR-TB is particularly severe in developing regions, with MDR-TB being the most prominent form of DR-TB. Additionally, the proportion of these types of DR-TB in High-middle SDI regions is the highest, with a particularly notable burden of XDR-TB and HIV/AIDS-XDR-TB in this region. In terms of age patterns, there has been a significant rise in the incidence and death burden of MDR-TB and XDR-TB among individuals aged 15–49 years. In contrast, the incidence and death burden of HIV/AIDS-MDR-TB and HIV/AIDS-XDR-TB have increased most significantly in the 5–14 age group. This trend indicates an urgent need for targeted strategies to effectively control various types of DR-TB, particularly in high-burden regions and high-risk age groups. Furthermore, it is essential to continuously improve the DR-TB prevention and treatment service system. Efforts to strengthen TB control programs, enhance diagnostic capacity, and address modifiable risk factors may help reduce the burden of DR-TB.

## Data Availability

The original contributions presented in the study are included in the article/Supplementary material, further inquiries can be directed to the corresponding author.

## Footnotes

1
http://ghdx.healthdata.org/GBD-results-tool
