# Supplementary material for: The burden of multidrug-resistant tuberculosis and extensively drug-resistant tuberculosis and its attributable risk factors in 204 countries and territories, 1990–2021
Source: Front Public Health. 2026 Oct 26;14:1960895. doi: 10.3389/fpubh.2026.1960895 (PMC13745727; doi:10.3389/fpubh.2026.1960895)
Supplement: Supplementary file 1 [file data_sheet_1.pdf]

Supplementary material

The burden of multidrug-resistant tuberculosis and extensively drug-resistant tuberculosis and its attributable risk factors in 204 countries and territories, 1990-2021

Hongping Wang†, Shan Lv†, Pu Yao and Jing Cen*

Department of Pharmacy, the First Affiliated Hospital of Army Medical University (Third Military Medical University), Chongqing, China

Contents

Table S1 GBD risk hierarchy with levels. 1
Table S2 GBD 2021 definitions and estimation methodology for risk factors 3
Table S3 The global and regional incidence of cases and ASR of drug-resistant tuberculosis in 2021, and the trends from baseline to 2021. 4
Table S4 The regional incidence of cases and ASR of drug-resistant tuberculosis in 2021, and the trends from baseline to 2021. 6
Table S5 The regional death of cases and ASR of drug-resistant tuberculosis in 2021, and the trends from baseline to 2021. 8
Table S6 The incidence of cases and ASR of MDR-TB in 2021, and the trends from baseline to 2021 by countries. 10
Table S7 The death of cases and ASR of MDR-TB in 2021, and the trends from baseline to 2021 by countries. 15
Table S8 The incidence of cases and ASR of XDR-TB in 2021, and the trends from baseline to 2021 by countries. 20
Table S9 The death of cases and ASR of XDR-TB in 2021, and the trends from baseline to 2021 by countries. 25
Table S10 The incidence of cases and ASR of HIV/AIDS-MDR-TB in 2021, and the trends from baseline to 2021 by countries. 30
Table S11 The death of cases and ASR of HIV/AIDS-MDR-TB in 2021, and the trends from baseline to 2021 by countries. 35
Table S12 The incidence of cases and ASR of HIV/AIDS-XDR-TB in 2021, and the trends from baseline to 2021 by countries. 40
Table S13 The death of cases and ASR of HIV/AIDS-XDR-TB in 2021, and the trends from baseline 2021 by countries. 45
Table S14 The global incidence of cases and ASR of drug-resistant tuberculosis in 2021, and the trends from baseline to 2021 by sex and age group. 50
Table S15 The global death of cases and ASR of drug-resistant tuberculosis in 2021, and the trends from baseline to 2021 by sex and age group. 52
Figure S1 The ASIR (A) and ASDR (B) per 100,000 population of MDR-TB, XDR-TB,

HIV/AIDS-MDR-TB, and HIV/AIDS-XDR-TB globally from baseline to 2021 55
Figure S2 The incidence ratio of MDR-TB, XDR-TB, HIV/AIDS-MDR-TB, and HIV/AIDS-MDR-TB from baseline to 2021 globally and in 5 territories 55
Figure S3 Temporal Trend of MDR-TB, XDR-TB, HIV/AIDS-MDR-TB, and HIV/AIDS-XDR-TB in global and 5 territories. (A) Fold change in incidence cases from baseline to 2021. (B) The EAPC of ASIR from baseline to 2021. (C) The incidence cases of MDR-TB, XDR-TB, HIV/AIDS-MDR-TB, and HIV/AIDS-XDR-TB from baseline to 2021(line chart) and the percentage distribution of global MDR-TB, XDR-TB, HIV/AIDS-MDR-TB, and HIV/AIDS-XDR-TB incidence cases across the 5 SDI regions in 2021(pie chart) 56
Figure S4 Temporal trend of MDR-TB, XDR-TB, HIV/AIDS-MDR-TB, and HIV/AIDS-XDR-TB in regions. (A) Fold change in incidence cases from baseline to 2021. (B) The EAPC of ASIR from baseline to 2021 57
Figure S5 Temporal trend of the EAPC of ASIR for MDR-TB (A), XDR-TB (B), HIV/AIDS-MDR-TB (C), and HIV/AIDS-XDR-TB (D) from baseline to 2021 across 204 countries 58
Figure S6 Temporal Trend of MDR-TB, XDR-TB, HIV/AIDS-MDR-TB, and HIV/AIDS-XDR-TB globally by sex pattern. (A) Fold change in incidence cases from baseline to 2021. (B) The EAPC of ASIR globally from baseline to 2021. (C) The ASIR per 100,000 population of MDR-TB, XDR-TB, HIV/AIDS-MDR-TB, and HIV/AIDS-XDR-TB globally from baseline to 2021 59
Figure S7 Temporal Trend of MDR-TB, XDR-TB, HIV/AIDS-MDR-TB, and HIV/AIDS-XDR-TB globally by sex pattern. (A) Fold change in death cases from baseline to 2021. (B) The EAPC of ASDR globally from baseline to 2021. (C) The ASDR per 100,000 population of MDR-TB, XDR-TB, HIV/AIDS-MDR-TB, and HIV/AIDS-XDR-TB globally from baseline to 2021. 60
Figure S8 Global temporal trend of MDR-TB, XDR-TB, HIV/AIDS-MDR-TB, and HIV/AIDS-XDR-TB by age pattern. (A) Incidence cases in 5 age groups from baseline to 2021. (B) Fold change in incidence cases across 5 age groups globally from baseline to 2021. (C) The EAPC of ASDR for 5 age groups globally from baseline to 2021. 61
Figure S9 Global ASIR per 100,000 population for MDR-TB, XDR-TB, HIV/AIDS-MDR-TB, and HIV/AIDS-XDR-TB across 5 age groups from baseline to 2021, stratified by sex 62
Figure S10 Global ASDR per 100,000 population for MDR-TB, XDR-TB, HIV/AIDS-MDR-TB, and HIV/AIDS-XDR-TB across 5 age groups from baseline to 2021, stratified by sex 63
Figure S11 Fold change in MDR-TB, XDR-TB, HIV/AIDS-MDR-TB, and HIV/AIDS-XDR-TB cases of incidence and death across 5 age groups globally from baseline to 2021, and the EAPC of ASIR and ASDR for 5 age groups globally from baseline to 2021, by sex 65
Figure S12 Percentage of death due to MDR-TB (A), XDR-TB (B), HIV/AIDS-MDR-TB(C), and HIV/AIDS-XDR-TB (D) attributable to significant risk factors in females globally, across 5 territories, and in 21 GBD regions in 2021 in females 65
Figure S13 Percentage of death due to MDR-TB (A), XDR-TB (B), HIV/AIDS-MDR-TB(C), and HIV/AIDS-XDR-TB (D) attributable to significant risk factors in males globally, across 5 territories, and in 21 GBD regions in 2021 in females 66

Risk factors

The GBD 2021 risk factor analysis utilized data from 54,561 distinct sources to generate epidemiological estimates for 88 risk factors and their associated health outcomes, covering a total of 631 risk-outcome pairs. The GBD 2021 framework categorizes risk factors into four levels, encompassing a total of 88 overarching risks, with detailed information provided in Table S1. This study examines the death risk proportion for MDR-TB, XDR-TB, HIV/AIDS-MDR-TB, and HIV/AIDS-XDR-TB in 2021, analyzing patterns by sex at both global and regional levels. In the analysis of MDR-TB and XDR-TB, six key risk factors were emphasized: smoking, high fasting plasma glucose, high alcohol consumption, high body mass index, low physical activity, and a diet high in processed meat. In contrast, the analysis of HIV/AIDS-MDR-TB and HIV/AIDS-XDR-TB primarily focused on three risk factors: unsafe sex, drug use, and intimate partner violence.

Table S1 GBD risk hierarchy with levels.

Risk	level
**All risk factors**	0
**Environmental/occupational risks**	1
Unsafe water, sanitation, and handwashing	2
Unsafe water source	3
Unsafe sanitation	3
No access to handwashing facility	3
Air pollution	2
Particulate matter pollution	3
Ambient particulate matter pollution	4
Household air pollution from solid fuels	4
Ambient ozone pollution	3
Nitrogen dioxide pollution	3
Non-optimal temperature	2
High temperature	3
Low temperature	3
Other environmental risks	2
Residential radon	3
Lead exposure	3
Occupational risks	2
Occupational carcinogens	3
Occupational exposure to asbestos	4
Occupational exposure to arsenic	4
Occupational exposure to benzene	4

Occupational exposure to beryllium	4
Occupational exposure to cadmium	4
Occupational exposure to chromium	4
Occupational exposure to diesel engine exhaust	4
Occupational exposure to formaldehyde	4
Occupational exposure to nickel	4
Occupational exposure to polycyclic aromatic hydrocarbons	4
Occupational exposure to silica	4
Occupational exposure to sulfuric acid	4
Occupational exposure to trichloroethylene	4
Occupational asthmagens	3
Occupational particulate matter, gases, and fumes	3
Occupational noise	3
Occupational injuries	3
Occupational ergonomic factors	3
**Behavioral risks**	1
Child and maternal malnutrition	2
Suboptimal breastfeeding	3
Non-exclusive breastfeeding	4
Discontinued breastfeeding	4
Child growth failure	3
Child underweight	4
Child wasting	4
Child stunting	4
Low birth weight and short gestation	3
Short gestation	4
Low birth weight	4
Iron deficiency	3
Vitamin A deficiency	3
Zinc deficiency	3
Tobacco	2
Smoking	3
Chewing tobacco	3
Secondhand smoke	3
High alcohol use	2
Drug use	2
Dietary risks	2
Diet low in fruits	3
Diet low in vegetables	3
Diet low in legumes	3
Diet low in whole grains	3
Diet low in nuts and seeds	3
Diet low in milk	3
Diet high in red meat	3

Diet high in processed meat	3
Diet high in sugar-sweetened beverages	3
Diet low in fiber	3
Diet low in calcium	3
Diet low in seafood omega-3 fatty acids	3
Diet low in omega-6 polyunsaturated fatty acids	3
Diet high in trans fatty acids	3
Diet high in sodium	3
Intimate partner violence	2
Childhood sexual abuse and bullying	2
Childhood sexual abuse	3
Bullying victimization	3
Unsafe sex	2
Low physical activity	2
**Metabolic risks**	1
High fasting plasma glucose	2
High LDL cholesterol	2
High systolic blood pressure	2
High body-mass index	2
Low bone mineral density	2
Kidney dysfunction	2

Table S2 GBD 2021 definitions and estimation methodology for risk factors

Risk factor	GBD definition	Exposure modelling
Smoking	Current smokers: individuals who currently use any smoked tobacco product on a daily or occasional basis. Former smokers: individuals who quit using all smoked tobacco products for at least six months (or according to the survey definition).	ST-GPR for current and former smoking prevalence; supply-side data (USDA/FAO/Euromonitor) scaled to consumption envelope; ensemble distributions for cigarettes per smoker per day, pack-years, and years since quitting.
High alcohol use	Grams per day of pure alcohol consumed among current drinkers. Current drinkers: proportion of individuals who consumed at least one alcoholic beverage (or some approximation) in a 12-month period.	ST-GPR for current drinker prevalence, LPC, and tourists; gamma distribution for individual-level intake; LPC adjusted for unrecorded and tourist consumption.
High fasting plasma glucose (HFPG)	Mean fasting plasma glucose in a population (continuous exposure in mmol/L); high FPG defined as any level above the TMREL.	ST-GPR for mean FPG by location, year, age, and sex; ensemble distribution from individual-level FPG data; covariate: age-specific prevalence of obesity.
High body-mass index (BMI)	Adults (ages 20+): BMI greater than 20 to 23 kg/m². Children and adolescents (ages 2–19): overweight or obese based on International Obesity Task Force (IOTF) standards.	ST-GPR for overweight and obesity prevalence; nested hierarchical mixed-effects model for mean BMI; ensemble distribution for BMI distribution.
Low physical activity	Objectively measured total physical activity less than 3600 to 4400 MET-minutes per week. Assessed for adults older than 25 years, for duration of at least ten minutes at a time, across all domains of life	Linear mixed-effects regression to capture relationship of reported MET-minutes/week with activity categories; accelerometer adjustment via MR-BRT; ST-GPR for complete estimates.

	(leisure/recreation, work, household, and transport).	
Diet high in processed meat	Average daily consumption (in grams per day) of meat preserved by smoking, curing, salting, or addition of chemical preservatives.	ST-GPR for mean intake by age, sex, location, and year; crosswalk via MR-BRT to adjust non-gold-standard data sources; ensemble distribution for intake distribution.
Unsafe sex	Risk of disease due to sexual transmission. Outcomes: HIV, cervical cancer, and all sexually transmitted diseases (STDs) except those in neonates from vertical transmission.	Three DisMod-MR 2.1 models (HIV transmission due to sex, injecting drug use, and other routes) adjusted to sum to 100%; age–sex patterns derived from the IDU–hepatitis B PAF; modelled in complement space.
Drug use	Injecting drug use (IDU) prevalence; drug use disorders.	DisMod-MR 2.1 for IDU prevalence (single-parameter prevalence model); cohort method for lifetime risk of hepatitis B/C infection.
Intimate partner violence (IPV)	Females only: ever having experienced one or more acts of physical and/or sexual violence by a current or former intimate partner since age 15. Physical violence: being slapped, pushed, shoved, hit, kicked, dragged, beaten, choked, burnt, or threatened with a weapon. Sexual violence: being physically forced to have intercourse, having intercourse out of fear, or being forced to do something humiliating or degrading.	ST-GPR for lifetime IPV prevalence; MR-BRT network meta-analysis for alternate case definitions (spline on age); ever-partnered adjustment via DisMod-MR 2.1; Holt's linear trend method for forecasting and back-casting.

For each risk factor, GBD specifies a standardised case definition, estimates its exposure distribution via spatiotemporal Gaussian process regression (ST-GPR) or DisMod-MR 2.1, and derives relative risks (RRs) using meta-regression—Bayesian, regularised, trimmed (MR-BRT). The theoretical minimum risk exposure level (TMREL) defines the counterfactual exposure at which risk is minimised. Within the standard GBD comparative risk assessment framework, the population attributable fraction (PAF) quantifies the proportion of disease burden that would be averted if exposure were reduced to the TMREL. Accordingly, all percentages reported in this study represent PAFs—that is, the proportion of deaths or DALYs attributable to the risk factor.

Table S3 The global and regional incidence of cases and ASR of drug-resistant tuberculosis in 2021, and the trends from baseline to 2021.

	Number_2021	Fold change	ASR_2021	EAPC
		**MDR-TB**		
Global	443679.91 (259195.8,766545.37)	7.54	5.4161 (3.1676,9.3429)	2.05 (0.64,3.48)
**sex**				
Both	443679.91 (259195.8,766545.37)	7.54	5.4161 (3.1676,9.3429)	2.05 (0.64,3.48)
Male	252065.43 (149700.94,429851.38)	7.46	6.1971 (3.6847,10.5929)	1.81 (0.37,3.27)
Female	191614.48 (108256.9,339501.12)	7.65	4.7047 (2.6857,8.3476)	2.34 (0.97,3.72)
**Age**				
<5 years	13989.75 (8912.07,20803.98)	2.42	2.1255 (1.3541,3.1609)	0.94 (-0.35,2.25)
5-14 years	18525.09 (10032.34,33091.71)	3.64	1.3685 (0.7411,2.4446)	1.4 (0.09,2.72)
15-49 years	241399.26 (137487.23,419391.52)	8.46	6.1136 (3.4819,10.6213)	2.85 (1.4,4.33)
50-74 years	138495.88 (74822.84,252951.94)	8.31	8.4326 (4.5558,15.4015)	1.36 (-0.04,2.78)

75+ years	31269.92 (16819.31,59181.27)	7.98	10.8377 (5.8293,20.5114)	1.14 (-0.27,2.56)
**SDI regions**				
Low SDI	84409.35 (49697.99,144491.72)	24.76	11.1269 (6.5124,19.0517)	4.39 (2.5,6.32)
Low-middle SDI	179354.92 (72252.18,382931.84)	50.06	10.347 (4.2133,21.9903)	6.02 (3.7,8.39)
Middle SDI	119777.1 (57287.11,219649.82)	3.44	4.6288 (2.2234,8.3884)	-0.26 (-1.31,0.81)
High-middle SDI	57213.28 (35365.57,90233.24)	2.64	3.7717 (2.3346,5.8495)	-0.25 (-1.85,1.38)
High SDI	2828.58 (1648.1,5155.32)	0.13	0.2085 (0.123,0.3801)	-3.33 (-4.34,-2.31)
**XDR-TB**				
Global	24036.08 (17144.48,34586.57)	23.06	0.2905 (0.2083,0.4182)	7.24 (5.13,9.4)
**sex**				
Both	24036.08 (17144.48,34586.57)	23.06	0.2905 (0.2083,0.4182)	7.24 (5.13,9.4)
Male	15285.57 (10953.46,21940.34)	23.86	0.3747 (0.269,0.5381)	7.07 (4.87,9.32)
Female	8750.52 (6099.77,12830.08)	21.77	0.211 (0.1469,0.3079)	7.48 (5.54,9.46)
**Age**				
<5 years	441.64 (294.61,635)	7.48	0.0671 (0.0448,0.0965)	5.18 (3.56,6.83)
5-14 years	751.06 (458.17,1207.59)	10.49	0.0555 (0.0338,0.0892)	6.83 (5.33,8.36)
15-49 years	12860.96 (8757.56,18740.14)	23.43	0.3257 (0.2218,0.4746)	8.19 (5.94,10.49)
50-74 years	7806.54 (5153.08,11938.53)	25.27	0.4753 (0.3138,0.7269)	6.56 (4.5,8.65)
75+ years	2175.87 (1321.85,3555.5)	36.44	0.7541 (0.4581,1.2323)	6.27 (4.13,8.46)
**SDI regions**				
Low SDI	1527.69 (780.35,2989.05)	118.26	0.2346 (0.1167,0.4674)	11.05 (8.17,14.01)
Low-middle SDI	5868.99 (2894.64,11523.37)	86.26	0.3581 (0.1762,0.7014)	10.27 (7.53,13.07)
Middle SDI	6345.37 (3751.44,10442.76)	13.49	0.2465 (0.1463,0.3997)	5.09 (3.56,6.65)
High-middle SDI	9949.47 (6271.81,14412.87)	21.46	0.658 (0.4116,0.947)	7.85 (5.41,10.35)
High SDI	336.64 (212.57,582.93)	7.85	0.0233 (0.0149,0.0384)	3.76 (2.08,5.46)
**HIV/AIDS-MDR-TB**				
Global	45589.4 (31326.35,66723.02)	23.36	0.5546 (0.3811,0.8115)	4.71 (2.03,7.47)
**sex**				
Both	45589.4 (31326.35,66723.02)	23.36	0.5546 (0.3811,0.8115)	4.71 (2.03,7.47)
Male	21653.34 (14972.14,31626.13)	20.79	0.5223 (0.3611,0.7612)	4.44 (1.89,7.06)
Female	23936.06 (16491.11,35420.7)	26.27	0.5886 (0.4054,0.8723)	4.97 (2.16,7.86)
**Age**				
<5 years	701.63 (463.51,1032.97)	4.63	0.1066 (0.0704,0.1569)	1.04 (-2.4,4.6)
5-14 years	1378.07 (941.15,2036.63)	162.47	0.1018 (0.0695,0.1505)	11.85 (7.78,16.08)
15-49 years	33838.87 (23279.44,49655.63)	21.89	0.857 (0.5896,1.2576)	4.73 (2.07,7.46)
50-74 years	9211.91 (6326.05,13544.75)	39.26	0.5609 (0.3852,0.8247)	5.37 (2.86,7.95)
75+ years	458.91 (294.59,726.25)	13.49	0.1591 (0.1021,0.2517)	2.54 (0.75,4.36)
**SDI regions**				
Low SDI	13473.21 (7954.32,21578.97)	22.06	1.5197 (0.9007,2.4116)	2.61 (0.11,5.17)
Low-middle SDI	13194.2 (7640.05,20424.31)	34.72	0.7071 (0.4077,1.0974)	5.42 (2.4,8.54)
Middle SDI	14902.52 (7505.04,29959.05)	27.28	0.5527 (0.2793,1.1157)	5.21 (2.27,8.23)
High-middle SDI	3900.72 (2406,5908.01)	20.15	0.2631 (0.1618,0.3965)	5.16 (2.85,7.53)
High SDI	102.25 (64.97,167.07)	-0.50	0.0078 (0.005,0.013)	-4.34 (-4.69,-3.98)
**HIV/AIDS-XDR-TB**				

Global	1606.42 (1163.94,2182.83)	51.76	0.0194 (0.0141,0.0264)	9.96 (6.82,13.19)
**sex**				
Both	1606.42 (1163.94,2182.83)	51.76	0.0194 (0.0141,0.0264)	9.96 (6.82,13.19)
Male	986.44 (695.94,1368.61)	45.03	0.0237 (0.0167,0.0329)	9.38 (6.32,12.53)
Female	619.98 (456.4,824.96)	67.73	0.0151 (0.0111,0.0202)	11.04 (7.72,14.46)
**Age**				
<5 years	17.54 (12.66,23.63)	24.06	0.0027 (0.0019,0.0036)	6.57 (2.68,10.61)
5-14 years	21.23 (15.28,29.09)	95.50	0.0016 (0.0011,0.0021)	13.29 (9.73,16.96)
15-49 years	1254.21 (904.7,1699.66)	48.71	0.0318 (0.0229,0.043)	10.21 (7.05,13.47)
50-74 years	300.26 (206.94,425.22)	77.19	0.0183 (0.0126,0.0259)	10.1 (7.17,13.11)
75+ years	13.18 (6.98,27.97)	27.04	0.0046 (0.0024,0.0097)	6.82 (4.69,9)
**SDI regions**				
Low SDI	144.31 (88.74,224.95)	65.81	0.0163 (0.0102,0.0253)	8.36 (5.14,11.68)
Low-middle SDI	272.56 (167.34,437.47)	72.86	0.0148 (0.009,0.0239)	10.5 (6.55,14.6)
Middle SDI	401.2 (239.18,611.69)	41.46	0.0148 (0.0088,0.0225)	9.17 (6.11,12.32)
High-middle SDI	771.98 (477.44,1160.66)	60.37	0.0523 (0.0324,0.0785)	11.36 (8.28,14.53)
High SDI	15.59 (10.58,23.26)	5.11	0.0012 (0.0008,0.0018)	3.36 (2.07,4.65)

Abbreviations: MDR-TB=multidrug-resistant tuberculosis. XDR-TB=extensively drug-resistant tuberculosis. ASR=age-standardized rate. EAPC=estimated annual percentage change. SDI=Social Demographic Index.

Table S4 The regional incidence of cases and ASR of drug-resistant tuberculosis in 2021, and the trends from baseline to 2021.

Location	Incidence_Number_2021	Fold change	Incidence_ASR_2021	EAPC
		**MDR-TB**		
High-income Asia Pacific	534.82 (145.8,1675.46)	0.08	0.1709 (0.0465,0.5497)	-4.29 (-5.66,-2.89)
High-income North America	145.86 (60.36,350.94)	-0.62	0.0335 (0.0138,0.0803)	-4.2 (-4.88,-3.51)
Western Europe	591.82 (381.96,923.23)	0.34	0.1302 (0.082,0.2103)	0.03 (-0.75,0.83)
Australasia	48.75 (18.62,110.6)	4.40	0.1461 (0.0557,0.3221)	4.63 (4.06,5.2)
Andean Latin America	2777.06 (1369.04,5504.2)	4.16	4.1982 (2.0734,8.2863)	-0.44 (-1.93,1.07)
Tropical Latin America	2271.42 (515.82,6514.15)	64.84	0.9218 (0.2092,2.6447)	6.89 (4.17,9.69)
Central Latin America	1470.54 (623.49,2950.36)	18.28	0.5654 (0.2394,1.1289)	2.15 (0.17,4.17)
Southern Latin America	123.33 (35.23,383.72)	1.78	0.1706 (0.047,0.5351)	-0.56 (-2.06,0.97)
Caribbean	79.75 (27.76,187.31)	0.13	0.1627 (0.0566,0.3845)	-3.77 (-4.8,-2.74)
Central Europe	365.69 (169.5,692.3)	0.41	0.2583 (0.1166,0.4999)	-0.99 (-2.1,0.14)
Eastern Europe	39542.81 (24759.11,58985.47)	13.78	16.7294 (10.3821,24.7198)	4.61 (2.11,7.16)
Central Asia	10554.5 (7086,15012.94)	99.53	11.0672 (7.4529,15.8238)	9.03 (5.77,12.39)
North Africa and Middle East	4708.53 (2590.41,8876.14)	8.28	0.8414 (0.4678,1.5533)	2.06 (0.05,4.11)
South Asia	251660.37 (78448.22,566022.73)	78.45	14.4595 (4.5452,32.7482)	7.14 (4.61,9.73)

Southeast Asia	24441.76 (12728.27,42287.89)	8.18	3.5467 (1.8664,6.1111)	0.54 (-0.96,2.07)
East Asia	29344.52 (7476.67,82539.99)	-0.20	1.6711 (0.4269,4.7344)	-6.12 (-7.3,-4.92)
Oceania	469.26 (140.38,1072.97)	114.01	4.4693 (1.3347,10.2282)	11.18 (9.73,12.65)
Western Sub-Saharan Africa	20368.01 (9570.72,42195.64)	12.23	6.516 (3.0579,13.8767)	2.63 (0.83,4.47)
Eastern Sub-Saharan Africa	32545.34 (19409.14,53304.86)	47.30	11.6102 (6.9441,19.189)	4.93 (2.62,7.3)
Central Sub-Saharan Africa	9162.34 (2772.49,24002.3)	11.93	9.8287 (2.9128,25.8721)	2.58 (1.35,3.82)
Southern Sub-Saharan Africa	12473.43 (5634.79,26527.96)	10.61	15.6498 (7.1376,32.6342)	4.9 (3.2,6.63)
**XDR-TB**				
High-income Asia Pacific	80.14 (21.24,246.03)	8.08	0.0227 (0.0062,0.0708)	2.3 (0.21,4.42)
High-income North America	19.19 (7.97,45.8)	7.80	0.0043 (0.0018,0.0103)	5.24 (4.68,5.81)
Western Europe	89.3 (57.64,138.28)	19.77	0.0172 (0.0109,0.0274)	8.06 (6.1,10.05)
Australasia	6.35 (2.4,14.31)	78.38	0.0184 (0.007,0.0405)	14.59 (13.62,15.58)
Andean Latin America	231.02 (115.07,454.58)	32.58	0.3548 (0.1755,0.6935)	5.9 (3.69,8.15)
Tropical Latin America	177.25 (40.46,506.7)	145.49	0.072 (0.0165,0.2059)	11.97 (9.03,14.99)
Central Latin America	124.66 (53,243.84)	52.73	0.0485 (0.0206,0.0948)	7.36 (5.05,9.72)
Southern Latin America	18.11 (5.23,55.39)	23.47	0.0241 (0.0069,0.0753)	6.29 (3.85,8.79)
Caribbean	7.19 (2.47,17.29)	8.85	0.0145 (0.005,0.0351)	3.73 (2.29,5.18)
Central Europe	78.52 (36.84,148.29)	8.04	0.0552 (0.0251,0.1065)	6.25 (4.43,8.1)
Eastern Europe	8315.15 (5207.21,12402.39)	38.55	3.518 (2.1834,5.1981)	11.02 (8.05,14.07)
Central Asia	2238.45 (1513.34,3176.53)	126.69	2.3475 (1.5871,3.3518)	13.71 (10.16,17.38)
North Africa and Middle East	197.66 (102.62,405.39)	58.54	0.0373 (0.0196,0.0757)	8.06 (5.05,11.16)
South Asia	6713.98 (2182.45,15024.18)	163.16	0.4048 (0.1335,0.9115)	12.21 (9.1,15.4)
Southeast Asia	2432.13 (1281.09,4185.25)	28.48	0.3703 (0.1983,0.6352)	6.5 (4.38,8.67)
East Asia	2623.55 (673.95,7311.66)	3.36	0.1492 (0.0385,0.4193)	0.39 (-1.16,1.97)
Oceania	58.6 (17.28,133.36)	585.00	0.6426 (0.19,1.4895)	19.42 (17.05,21.85)
Western Sub-Saharan Africa	167.34 (79.13,360.17)	58.34	0.0623 (0.0295,0.1308)	9.3 (6.67,12.01)
Eastern Sub-Saharan Africa	283.31 (172.29,450.75)	135.86	0.1209 (0.0729,0.192)	10.71 (7.84,13.66)
Central Sub-Saharan Africa	75.96 (23.56,199.2)	60.76	0.0984 (0.0299,0.2601)	9.6 (7.48,11.77)
Southern Sub-Saharan Africa	98.23 (45.9,205.91)	113.22	0.1317 (0.0623,0.2853)	12.63 (9.39,15.96)
**HIV/AIDS-MDR-TB**				
High-income Asia Pacific	10.23 (2.89,31.28)	0.80	0.0043 (0.0012,0.0132)	-2.07 (-3.71,-0.41)
High-income North America	13.72 (5.43,32.98)	-0.89	0.003 (0.0012,0.0071)	-9.24 (-10.21,-8.26)
Western Europe	38.91 (23.52,63.11)	-0.48	0.0071 (0.0043,0.0114)	-5.27 (-5.97,-4.55)
Australasia	1.56 (0.58,3.61)	0.71	0.004 (0.0015,0.0093)	-0.64 (-1.52,0.25)
Andean Latin America	240.32 (108.27,493.1)	12.03	0.3502 (0.1582,0.7183)	1.89 (-0.64,4.48)
Tropical Latin America	549.23 (118.27,1539.15)	76.90	0.2152 (0.0464,0.6031)	6.87 (4,9.82)
Central Latin America	224.3 (95.53,452.89)	25.08	0.0841 (0.0358,0.1697)	3.05 (0.66,5.49)
Southern Latin America	45.71 (11.6,141.77)	3.79	0.06 (0.0153,0.186)	-0.43 (-2.42,1.6)
Caribbean	16.17 (5.63,39.66)	-0.13	0.0324 (0.0112,0.08)	-5.9 (-7.41,-4.37)
Central Europe	8.97 (3.95,18.12)	0.62	0.0074 (0.0032,0.0153)	-1.39 (-2.84,0.07)
Eastern Europe	3434.58 (2084.29,5233.23)	36.75	1.4756 (0.8952,2.2521)	7.72 (4.92,10.58)
Central Asia	242.64 (151.3,369.58)	196.27	0.2357 (0.1472,0.3594)	9.9 (6.22,13.71)
North Africa and Middle East	136.72 (74.44,259.44)	28.53	0.0214 (0.0117,0.0409)	4.46 (1.94,7.05)
South Asia	6325.06 (1714.16,14983.9)	940.23	0.3418 (0.0916,0.8108)	11.74 (6.57,17.16)

Southeast Asia	1722.56 (879.53,3019.1)	30.43	0.2257 (0.1151,0.3953)	4.09 (1.46,6.79)
East Asia	1141.24 (228.83,3390.48)	2.69	0.0642 (0.013,0.1888)	-1.84 (-3.42,-0.24)
Oceania	54.4 (15.85,132.01)	1812.33	0.4396 (0.128,1.0666)	19.95 (16.32,23.69)
Western Sub-Saharan Africa	4728.33 (2160.5,9841.68)	11.73	1.3378 (0.6114,2.7784)	1.38 (-0.99,3.79)
Eastern Sub-Saharan Africa	12361.93 (6912.27,20663.83)	56.38	3.8194 (2.1591,6.2608)	4.03 (0.9,7.26)
Central Sub-Saharan Africa	1169.45 (433.9,2581.43)	8.67	1.1116 (0.4116,2.4185)	0.57 (-1.44,2.61)
Southern Sub-Saharan Africa	13123.4 (6235.44,28458.34)	30.25	16.4002 (7.7927,35.6501)	6.41 (3.15,9.77)
**HIV/AIDS-XDR-TB**				
High-income Asia Pacific	1.47 (0.43,4.37)	11.25	6e-04 (2e-04,0.0018)	5 (2.68,7.38)
High-income North America	2.23 (0.87,5.5)	1.08	5e-04 (2e-04,0.0012)	-0.64 (-1.25,-0.01)
Western Europe	7.52 (4.39,12.47)	4.74	0.0014 (8e-04,0.0023)	1.95 (0.55,3.36)
Australasia	0.19 (0.07,0.45)	18.00	5e-04 (2e-04,0.0012)	7.99 (7.38,8.6)
Andean Latin America	21.45 (9.8,43.74)	58.58	0.0316 (0.0145,0.0643)	8.17 (4.49,11.99)
Tropical Latin America	48.64 (11.08,135.23)	134.11	0.019 (0.0043,0.0529)	11.3 (8.16,14.54)
Central Latin America	25.16 (10.91,48.98)	73.00	0.0094 (0.0041,0.0184)	8.2 (5.26,11.22)
Southern Latin America	8.56 (2.25,26.08)	31.92	0.0113 (0.003,0.0344)	6.68 (3.75,9.7)
Caribbean	2.13 (0.7,5.44)	5.09	0.0043 (0.0014,0.0109)	0.8 (-1.19,2.82)
Central Europe	2.26 (0.97,4.64)	8.04	0.0019 (8e-04,0.0039)	4.93 (2.85,7.05)
Eastern Europe	724.35 (439.64,1103.31)	71.36	0.3118 (0.1892,0.4758)	13.06 (9.7,16.52)
Central Asia	53.74 (33.43,81.31)	166.94	0.0523 (0.0326,0.0791)	13.16 (9.14,17.32)
North Africa and Middle East	5.75 (3.17,10.49)	114.00	9e-04 (5e-04,0.0017)	9.71 (6.35,13.18)
South Asia	162.02 (45.42,382.6)	206.72	0.0088 (0.0024,0.0208)	14.07 (8.74,19.67)
Southeast Asia	172.57 (86.26,305.22)	41.19	0.0225 (0.0113,0.0396)	10.8 (7.67,14.01)
East Asia	104.15 (20.6,311.71)	13.13	0.0059 (0.0012,0.0175)	4.78 (2.6,7.01)
Oceania	7.3 (2.13,17.71)	2800.14	0.0605 (0.0176,0.1469)	27.16 (22.5,32.01)
Western Sub-Saharan Africa	41.96 (19.78,84.06)	38.58	0.0121 (0.0057,0.0242)	6.69 (3.59,9.89)
Eastern Sub-Saharan Africa	107.24 (60.22,180.27)	89.12	0.033 (0.0189,0.0544)	8.25 (4.53,12.11)
Central Sub-Saharan Africa	10.17 (4.01,22.08)	30.78	0.0097 (0.0039,0.021)	5.94 (3.05,8.91)
Southern Sub-Saharan Africa	97.56 (47.99,205.83)	116.54	0.1225 (0.0601,0.2582)	11.12 (6.38,16.07)

Abbreviations: MDR-TB=multidrug-resistant tuberculosis. XDR-TB=extensively drug-resistant tuberculosis. ASR=age-standardized rate. EAPC=estimated annual percentage change.

Table S5 The regional death of cases and ASR of drug-resistant tuberculosis in 2021, and the trends from baseline to 2021.

Location	Deaths_Number_2021	Fold change	Deaths_ASR_2021	EAPC
**MDR-TB**				
High-income Asia Pacific	128.24 (25.85,406.73)	-0.24	0.0218 (0.0044,0.0709)	-8.12 (-9.5,-6.72)
High-income North America	26.8 (7.62,76.43)	-0.83	0.0043 (0.0012,0.0123)	-7.63 (-8.81,-6.44)
Western Europe	124.91 (52.12,244.05)	-0.10	0.0119 (0.005,0.0232)	-3.56 (-4.24,-2.88)
Australasia	5.07 (1.35,13.42)	2.11	0.0089 (0.0024,0.0233)	0.34 (-0.27,0.96)
Andean Latin America	547.91 (198.34,1210.13)	1.45	0.891 (0.3227,1.9684)	-3.11 (-4.48,-1.71)
Tropical Latin America	341.69 (64.49,1006.68)	35.16	0.1342 (0.0253,0.3935)	4.39 (1.49,7.37)

Central Latin America	347.19 (114.56,755.46)	8.99	0.1376 (0.0452,0.2983)	-0.39 (-2.42,1.69)
Southern Latin America	30.64 (6.47,90.96)	0.60	0.0367 (0.0077,0.1094)	-2.91 (-4.22,-1.58)
Caribbean	23.76 (4.72,75.23)	-0.16	0.0471 (0.0092,0.1476)	-4.8 (-5.73,-3.86)
Central Europe	62.52 (19.96,134.13)	-0.16	0.0342 (0.0109,0.0746)	-3.89 (-5.17,-2.59)
Eastern Europe	2770.04 (1583.11,3970.74)	6.12	0.9667 (0.5525,1.3903)	1.97 (-1,5.03)
Central Asia	1139.54 (629.6,1760.1)	44.45	1.2067 (0.6681,1.8618)	5.24 (1.29,9.35)
North Africa and Middle East	1364.39 (431.98,3416.91)	7.26	0.2796 (0.0908,0.6973)	1.24 (-1.05,3.58)
South Asia	64155.45 (15726.4,147523.04)	39.56	4.2227 (1.0393,9.6668)	5.52 (2.83,8.27)
Southeast Asia	5828.56 (2127.52,12521.8)	3.70	0.9508 (0.3546,2.0279)	-1.46 (-3.25,0.37)
East Asia	3904.18 (1022.97,9735.87)	-0.61	0.1932 (0.0513,0.479)	-9.06 (-10.2,-7.9)
Oceania	196.23 (43.33,515.95)	88.60	2.3615 (0.518,6.2555)	10.65 (9.05,12.27)
Western Sub-Saharan Africa	6978.4 (2365.31,15585.29)	9.25	3.0665 (1.0652,6.6235)	2.28 (0.48,4.1)
Eastern Sub-Saharan Africa	12409 (4807.94,25856.18)	30.47	6.2053 (2.4002,12.5191)	4.55 (2.06,7.1)
Central Sub-Saharan Africa	3466.56 (865.15,11863.34)	7.77	5.0956 (1.245,17.4271)	1.93 (0.59,3.29)
Southern Sub-Saharan Africa	2966.89 (1071.36,6629.57)	10.44	4.5255 (1.6542,10.1548)	4.42 (2.38,6.5)
		**XDR-TB**		
High-income Asia Pacific	34.41 (7.17,110.15)	8.78	0.0058 (0.0012,0.0181)	-0.89 (-3.13,1.4)
High-income North America	7.19 (2.04,19.09)	5.15	0.0012 (3e-04,0.0031)	2.79 (2.29,3.29)
Western Europe	33.64 (13.59,71.27)	17.18	0.0032 (0.0013,0.0068)	5.06 (3.07,7.09)
Australasia	1.36 (0.35,3.47)	67.00	0.0024 (6e-04,0.0061)	10.51 (9.31,11.73)
Andean Latin America	92.83 (31.91,216.33)	27.48	0.151 (0.0518,0.3526)	4.35 (2.02,6.73)
Tropical Latin America	57.92 (9.69,178.81)	221.77	0.0227 (0.0038,0.0702)	10.3 (6.74,13.98)
Central Latin America	58.76 (20.29,135.41)	59.58	0.0233 (0.008,0.0534)	6.16 (3.62,8.76)
Southern Latin America	8.26 (1.71,26.63)	20.74	0.0099 (0.002,0.0321)	4.98 (2.52,7.49)
Caribbean	4.04 (0.8,14.08)	11.24	0.008 (0.0016,0.0276)	3.53 (1.9,5.17)
Central Europe	29.15 (9.31,67.01)	9.09	0.016 (0.0051,0.0372)	3.98 (1.8,6.2)
Eastern Europe	1295.54 (713.14,2011.74)	36.02	0.4521 (0.2486,0.7024)	8.07 (4.45,11.81)
Central Asia	532.26 (296.21,852.31)	147.26	0.5636 (0.3147,0.9017)	10.16 (5.88,14.62)
North Africa and Middle East	104.1 (30.87,299.21)	87.22	0.0213 (0.0066,0.0595)	7.75 (4.43,11.19)
South Asia	3387.7 (809.04,8074.66)	220.85	0.223 (0.053,0.534)	11.68 (8.23,15.25)
Southeast Asia	1138.17 (386.02,2638.52)	31.61	0.1857 (0.0625,0.4278)	5.4 (2.91,7.96)
East Asia	763.72 (189.27,2010.01)	4.06	0.0378 (0.0095,0.0989)	-1.75 (-3.75,0.29)
Oceania	38.13 (8.49,104.31)	761.60	0.4588 (0.1005,1.2561)	19.33 (16.77,21.95)
Western Sub-Saharan Africa	96.91 (29.56,219.59)	74.71	0.0426 (0.0135,0.0943)	9.35 (6.59,12.18)
Eastern Sub-Saharan Africa	172.5 (65.75,357.65)	161.74	0.0863 (0.0323,0.1782)	10.66 (7.62,13.78)
Central Sub-Saharan Africa	48.54 (11.33,167.86)	65.49	0.0714 (0.0171,0.2414)	9.54 (7.31,11.81)
Southern Sub-Saharan Africa	41.32 (14.2,99.9)	128.13	0.063 (0.0215,0.1528)	12.18 (8.6,15.88)
		**HIV/AIDS-MDR-TB**		
High-income Asia Pacific	2.81 (0.64,8.79)	3.26	0.0011 (3e-04,0.0034)	-0.64 (-2.72,1.49)
High-income North America	6.33 (1.69,18.51)	-0.93	0.0013 (4e-04,0.0039)	-10.74 (-12.12,-9.35)
Western Europe	20.93 (7.33,47.42)	-0.57	0.0039 (0.0014,0.0087)	-6.34 (-7.12,-5.55)
Australasia	0.39 (0.09,1.06)	-0.03	0.0011 (3e-04,0.0029)	-3.07 (-4.08,-2.04)
Andean Latin America	88.65 (27.86,196.46)	23.83	0.1317 (0.0414,0.2919)	3.96 (0.53,7.5)
Tropical Latin America	189.74 (32.2,592.81)	57.56	0.0733 (0.0124,0.229)	5.94 (2.66,9.33)

Central Latin America	114.31 (33.66,246.67)	21.07	0.0429 (0.0126,0.0925)	2.48 (-0.21,5.25)
Southern Latin America	24.81 (4.8,83.31)	7.64	0.0328 (0.0064,0.1102)	0.75 (-1.76,3.32)
Caribbean	9.74 (1.93,32.05)	-0.24	0.0195 (0.0039,0.0637)	-6.22 (-7.73,-4.68)
Central Europe	3.32 (0.93,7.54)	0.24	0.0028 (8e-04,0.0065)	-2.3 (-3.88,-0.69)
Eastern Europe	555.04 (280.33,924.28)	18.40	0.2314 (0.1171,0.3857)	5.78 (2.49,9.16)
Central Asia	54.82 (25.2,89.59)	106.49	0.0535 (0.0246,0.0874)	7.81 (3.36,12.45)
North Africa and Middle East	47.79 (15.99,99.9)	38.50	0.0077 (0.0026,0.0166)	4.6 (1.38,7.92)
South Asia	1691.13 (398.04,3936.55)	6763.52	0.0916 (0.0212,0.214)	17.42 (9.09,26.39)
Southeast Asia	615.83 (227.11,1254.86)	33.44	0.0799 (0.0294,0.1626)	7.63 (4.41,10.94)
East Asia	310.76 (50.57,942.33)	5.68	0.017 (0.0028,0.0512)	-0.5 (-2.81,1.86)
Oceania	29.99 (6.69,76.08)	2998.00	0.2486 (0.0551,0.6447)	24.16 (19.3,29.23)
Western Sub-Saharan Africa	2327.98 (715.07,5439.05)	10.85	0.6725 (0.2065,1.5835)	1.13 (-1.46,3.78)
Eastern Sub-Saharan Africa	5925.99 (2418.74,12025.23)	60.69	1.8118 (0.7447,3.7185)	4.71 (0.79,8.79)
Central Sub-Saharan Africa	560.41 (167.08,1348.11)	8.04	0.535 (0.1603,1.2661)	0.23 (-2.22,2.73)
Southern Sub-Saharan Africa	4876.84 (1803.52,10740.13)	60.67	6.1324 (2.2725,13.4662)	7.02 (2.75,11.48)
**HIV/AIDS-XDR-TB**				
High-income Asia Pacific	0.75 (0.18,2.19)	36.50	3e-04 (1e-04,9e-04)	6.14 (3.27,9.1)
High-income North America	1.7 (0.45,4.9)	1.05	4e-04 (1e-04,0.001)	-0.79 (-1.44,-0.13)
Western Europe	5.63 (1.98,12.03)	5.12	0.001 (4e-04,0.0022)	1.84 (0.38,3.32)
Australasia	0.11 (0.02,0.29)	10.00	3e-04 (1e-04,8e-04)	6.88 (6.04,7.73)
Andean Latin America	15.03 (4.4,35.67)	166.00	0.0223 (0.0065,0.053)	9.6 (5.16,14.24)
Tropical Latin America	32.11 (5.09,109.66)	246.00	0.0124 (0.002,0.0424)	11.3 (7.64,15.09)
Central Latin America	19.34 (5.85,44.62)	95.70	0.0073 (0.0022,0.0168)	8.36 (5.24,11.58)
Southern Latin America	6.69 (1.29,22.88)	50.46	0.0089 (0.0017,0.0303)	7.08 (3.86,10.41)
Caribbean	1.66 (0.32,5.72)	5.64	0.0033 (6e-04,0.0114)	0.84 (-1.18,2.91)
Central Europe	1.55 (0.44,3.58)	10.92	0.0013 (4e-04,0.0031)	5.19 (2.91,7.53)
Eastern Europe	259.41 (127.41,429.03)	78.82	0.1082 (0.0532,0.179)	11.83 (7.92,15.88)
Central Asia	25.61 (11.74,45.13)	283.56	0.025 (0.0115,0.0441)	12.28 (7.6,17.18)
North Africa and Middle East	3.63 (1.17,8.14)	180.50	6e-04 (2e-04,0.0013)	9.96 (6.09,13.97)
South Asia	89.45 (19.72,228.14)	1788.00	0.0048 (0.0011,0.0124)	16.62 (9.36,24.35)
Southeast Asia	120.49 (41.39,278.23)	210.39	0.0156 (0.0054,0.0359)	14.01 (9.65,18.54)
East Asia	60.74 (8.75,204.47)	38.19	0.0033 (5e-04,0.0111)	5.93 (2.89,9.07)
Oceania	5.82 (1.22,14.92)	17964.85	0.0483 (0.0103,0.1248)	30.42 (24.09,37.08)
Western Sub-Saharan Africa	32.27 (9.59,72.81)	55.61	0.0093 (0.0028,0.0211)	6.97 (3.67,10.37)
Eastern Sub-Saharan Africa	82.32 (31.84,173.57)	174.15	0.0252 (0.0099,0.0522)	8.84 (4.69,13.16)
Central Sub-Saharan Africa	7.8 (2.2,21.68)	38.00	0.0075 (0.0021,0.0205)	6.06 (3.06,9.15)
Southern Sub-Saharan Africa	67.87 (23.18,163.69)	217.94	0.0853 (0.0291,0.2052)	11.81 (6.55,17.33)

Abbreviations: MDR-TB=multidrug-resistant tuberculosis. XDR-TB=extensively drug-resistant tuberculosis. ASR=age-standardized rate0. EAPC=estimated annual percentage change.

Table S6 The incidence of cases and ASR of MDR-TB in 2021, and the trends from baseline to 2021 by countries.

Location	Incidence_Number_2021	Incidence_ASR_2021	EAPC

Afghanistan	1477.6 (201.93,5361.7)	8.1019 (1.0819,29.9512)	5.49 (2.46,8.6)
Albania	2.33 (0.41,7.64)	0.0712 (0.0125,0.2341)	-1.55 (-3.03,-0.05)
Algeria	335.35 (42.05,1208.97)	0.8602 (0.1072,3.1217)	-2.69 (-3.08,-2.29)
American Samoa	0.02 (0,0.08)	0.0429 (0.0044,0.1736)	-2.29 (-3.62,-0.94)
Andorra	0.01 (0,0.04)	0.0099 (0.0013,0.0368)	-3.86 (-4.46,-3.26)
Angola	1911.41 (299.41,5433.89)	8.6978 (1.3485,25.2161)	1.94 (0.58,3.32)
Antigua and Barbuda	0.08 (0.01,0.33)	0.0804 (0.0103,0.3509)	-6.11 (-7.28,-4.92)
Argentina	93.55 (14.86,344.93)	0.1961 (0.0311,0.7238)	-0.69 (-2.38,1.03)
Armenia	111.11 (25.83,270.95)	3.3267 (0.7713,8.256)	10.13 (6.56,13.82)
Australia	43.38 (13.26,104.33)	0.155 (0.0472,0.3686)	5.46 (4.81,6.12)
Austria	14.59 (4.56,33.65)	0.1523 (0.0481,0.3662)	3.81 (2.76,4.86)
Azerbaijan	1228.96 (371.23,2498.2)	10.8743 (3.3311,22.1538)	14.11 (10.5,17.85)
Bahrain	5.53 (0.84,22.1)	0.4314 (0.0662,1.6832)	2.72 (0.33,5.17)
Bangladesh	8607.37 (2418.94,22375.54)	5.6304 (1.5953,14.7025)	4.23 (1.5,7.03)
Barbados	0.05 (0,0.2)	0.0139 (0.0012,0.0589)	-10.55 (-12.21,-8.86)
Belarus	907 (554.49,1395.39)	8.1059 (4.9517,12.4658)	4.46 (2.25,6.71)
Belgium	13.29 (3.4,36)	0.1157 (0.0299,0.3054)	-0.77 (-1.41,-0.14)
Belize	0.41 (0.05,1.69)	0.1047 (0.0122,0.4326)	-5.65 (-6.88,-4.41)
Benin	229.89 (44.46,667.59)	2.6647 (0.5105,7.7308)	3.34 (1.42,5.31)
Bermuda	0.05 (0,0.22)	0.0639 (0.0048,0.3229)	-6.53 (-8.15,-4.88)
Bhutan	33.19 (5.49,98.62)	4.6059 (0.7662,13.956)	1.69 (0.27,3.13)
Bolivia	398.2 (66.06,1282.13)	3.6787 (0.6087,11.7517)	-1.54 (-2.75,-0.31)
Bosnia and Herzegovina	3.53 (0.76,11.16)	0.0774 (0.0169,0.2451)	-2.32 (-2.97,-1.65)
Botswana	355.75 (72.15,1072.2)	14.896 (2.9904,43.3944)	8.89 (7.45,10.35)
Brazil	2210.3 (465.65,6382.44)	0.9239 (0.1939,2.6774)	7.2 (4.41,10.05)
Brunei	0.68 (0.08,2.59)	0.1689 (0.0189,0.6209)	-2.85 (-4.17,-1.51)
Bulgaria	22.25 (5.12,62.7)	0.2718 (0.0649,0.778)	3.39 (1.26,5.57)
Burkina Faso	801.53 (145.07,2175.46)	5.5791 (1.0235,14.9767)	2.33 (0.96,3.72)
Burundi	1116.52 (180.81,3684.82)	14.2926 (2.3409,48.2178)	4.44 (2.15,6.78)
Cabo Verde	14.45 (2.3,40.05)	2.8152 (0.4483,7.7617)	0.59 (-0.77,1.97)
Cambodia	287.88 (44.53,988.78)	1.9727 (0.3043,6.7676)	-2.33 (-5.06,0.47)
Cameroon	758.3 (128.52,2314.43)	3.5816 (0.6187,10.7981)	0.87 (-0.67,2.43)
Canada	24.28 (7.53,59.95)	0.0551 (0.0167,0.135)	-0.98 (-1.26,-0.7)
Central African Republic	420.49 (71.01,1424.56)	12.1023 (2.0268,40.0932)	1.14 (-0.06,2.35)
Chad	750.81 (125.11,2194.1)	7.2875 (1.2618,20.7117)	2.13 (0.72,3.56)
Chile	27.23 (7.48,70.31)	0.1243 (0.0342,0.3197)	-0.46 (-1.15,0.24)
China	25431.09 (4308.2,78863.87)	1.4932 (0.2544,4.6586)	-6.6 (-7.8,-5.38)
Colombia	422.81 (81.98,1271.1)	0.7876 (0.1521,2.3731)	3.95 (2.5,5.42)
Comoros	151.25 (20.21,461.14)	25.4969 (3.3972,79.3347)	7.94 (5.91,10.01)
Congo (Brazzaville)	235.77 (36.57,794.49)	5.711 (0.8826,19.3978)	1.03 (-0.26,2.32)
Cook Islands	0.01 (0,0.03)	0.0326 (0.0039,0.1319)	-2.31 (-3.51,-1.11)
Costa Rica	7.45 (1.17,24.87)	0.1418 (0.0223,0.473)	0.28 (-2.23,2.86)
Côte d'Ivoire	1058.87 (198.92,3322.51)	5.5665 (1.0587,17.0469)	-3.02 (-4.04,-1.99)
Croatia	1.26 (0.31,3.88)	0.0213 (0.0052,0.0654)	-6.03 (-7.24,-4.8)

Cuba	13.1 (2.51,39.85)	0.0993 (0.019,0.3016)	-2.9 (-3.64,-2.15)
Cyprus	0.85 (0.16,2.8)	0.0564 (0.0107,0.1834)	-1.52 (-3.69,0.7)
Czechia	8.6 (2.51,22.05)	0.0651 (0.0191,0.1715)	-1.8 (-2.45,-1.14)
Denmark	3.85 (0.94,10.53)	0.0647 (0.0158,0.1755)	3.11 (2.62,3.61)
Djibouti	169.05 (63.14,409.66)	19.2621 (7.183,46.8238)	11.21 (7.9,14.62)
Dominica	0.08 (0.01,0.25)	0.1028 (0.014,0.3463)	-7.6 (-8.83,-6.35)
Dominican Republic	23.22 (3.06,90.37)	0.2104 (0.0278,0.8183)	-5.69 (-7.12,-4.24)
DR Congo	6459.33 (1112.31,19718.92)	10.5585 (1.8305,32.7837)	2.98 (1.78,4.2)
Ecuador	361.09 (63.28,1189.32)	2.0066 (0.3505,6.6586)	-0.46 (-2.77,1.91)
Egypt	398.16 (93.79,1202.27)	0.4582 (0.1092,1.3882)	3.33 (1.04,5.67)
El Salvador	41.02 (6.63,133.8)	0.6397 (0.1038,2.0921)	-2.29 (-4.33,-0.21)
Equatorial Guinea	48.86 (8.15,166.57)	4.3665 (0.7205,15.2514)	-1.69 (-2.76,-0.61)
Eritrea	1007.77 (186.02,2897.33)	23.0983 (4.2333,65.2672)	6.12 (3.95,8.33)
Estonia	32.59 (14.12,62.31)	2.1135 (0.8943,4.0515)	-2.97 (-4.17,-1.77)
Eswatini	496.41 (116.83,1266.06)	45.5615 (10.8334,118.3807)	9.48 (7.08,11.94)
Ethiopia	6778.58 (991.51,21633.06)	9.6087 (1.4433,30.485)	3.07 (0.75,5.44)
Federated States of Micronesia	0.23 (0.02,1.02)	0.2454 (0.0235,1.1104)	-2.03 (-3.26,-0.77)
Fiji	0.53 (0.06,2.35)	0.0618 (0.0066,0.2744)	1.84 (0.93,2.75)
Finland	7.71 (2.01,21.33)	0.1137 (0.0295,0.3139)	5.63 (5.12,6.13)
France	84.62 (22.95,224.49)	0.1064 (0.03,0.2812)	0.02 (-0.61,0.65)
Gabon	86.49 (14.96,280.3)	5.7658 (1.017,18.8098)	3.26 (1.98,4.56)
Georgia	383.17 (184.2,653.87)	9.7899 (4.6654,17.0011)	9.95 (6.98,13)
Germany	168.44 (53.2,389.68)	0.1978 (0.0612,0.4585)	3.01 (2.6,3.41)
Ghana	1293.33 (198.72,4285.41)	5.582 (0.858,18.8734)	1.75 (0.7,2.81)
Greece	12.48 (2.4,37.37)	0.0929 (0.0176,0.2728)	3.78 (2.12,5.46)
Greenland	0.95 (0.14,3.37)	1.4936 (0.2136,5.3391)	0.43 (-0.02,0.88)
Grenada	0.06 (0.01,0.24)	0.0554 (0.0068,0.2285)	-3.56 (-4.76,-2.35)
Guam	0.23 (0.03,0.93)	0.1314 (0.0155,0.5259)	0.88 (-1.61,3.44)
Guatemala	67.31 (11.29,195.36)	0.4933 (0.0817,1.4328)	-2.29 (-4.25,-0.29)
Guinea	343.28 (62.07,1025.94)	4.0705 (0.7269,12.0385)	1.63 (0.31,2.96)
Guinea-Bissau	67.09 (10.12,230.88)	6.3307 (0.9353,22.2141)	0.29 (-1.11,1.71)
Guyana	2 (0.23,8.89)	0.2687 (0.0312,1.199)	-0.96 (-2,0.09)
Haiti	33.92 (4.72,111.96)	0.3111 (0.043,1.0295)	-3.86 (-4.75,-2.97)
Honduras	80.46 (14.06,246.06)	1.0215 (0.1811,3.1223)	2.78 (0.54,5.07)
Hungary	7.07 (1.41,21.36)	0.0553 (0.0109,0.1678)	-2.98 (-5.19,-0.72)
Iceland	0.1 (0.02,0.33)	0.0271 (0.0043,0.0888)	-0.64 (-3.39,2.19)
India	219706.47 (46977.03,526082.81)	15.98 (3.4466,38.5228)	7.6 (4.98,10.28)
Indonesia	1785.15 (238.69,6466.81)	0.7294 (0.0996,2.6312)	-5.85 (-7.96,-3.69)
Iran	211.15 (65.28,529.98)	0.2564 (0.0785,0.6459)	4.86 (2.58,7.19)
Iraq	270.77 (73.38,677.54)	0.8299 (0.2243,2.1229)	4.35 (1.53,7.24)
Ireland	3.33 (0.65,9.84)	0.0652 (0.0126,0.1905)	0.04 (-0.86,0.94)
Israel	14.36 (3.7,38.29)	0.1458 (0.0369,0.3901)	-2.79 (-3.72,-1.85)
Italy	92.35 (27.22,235.97)	0.1631 (0.0476,0.4144)	-0.55 (-2.53,1.48)
Jamaica	1.45 (0.22,5.94)	0.0496 (0.0075,0.1999)	-1.14 (-2.11,-0.17)

Japan	144.15 (17.61,554.08)	0.0511 (0.0062,0.1853)	-6.66 (-7.88,-5.43)
Jordan	25.21 (4.54,90.03)	0.2448 (0.0439,0.876)	2 (-0.47,4.53)
Kazakhstan	2109.08 (991.63,3755.53)	10.9946 (5.1729,19.4374)	5.01 (1.48,8.67)
Kenya	1871.74 (443.3,4352.63)	6.0089 (1.4304,13.9588)	6.38 (3.04,9.83)
Kiribati	1.06 (0.12,4.66)	1.0995 (0.1232,4.8152)	-0.98 (-2.38,0.45)
Kuwait	12.88 (2.84,35.61)	0.2784 (0.0627,0.7794)	1.32 (-0.55,3.22)
Kyrgyzstan	1286.83 (425.52,2552.97)	20.1623 (6.6863,40.0473)	14.75 (11.04,18.58)
Laos	36.93 (4.98,157.2)	0.6501 (0.0876,2.7326)	-7.42 (-9.48,-5.32)
Latvia	47.11 (19.94,88.09)	2.2407 (0.9278,4.2259)	-5.63 (-6.16,-5.09)
Lebanon	13.72 (1.78,55.52)	0.2343 (0.0304,0.952)	0.78 (-1.1,2.71)
Lesotho	572.65 (167.54,1449.11)	36.0303 (10.0827,93.2855)	12 (9.34,14.73)
Liberia	105.14 (16.22,343.03)	2.9653 (0.4482,9.7515)	-2.08 (-3.36,-0.78)
Libya	33.31 (3.79,126.75)	0.5288 (0.0601,1.9695)	1.77 (-0.33,3.9)
Lithuania	182.36 (86.78,307.62)	5.7539 (2.763,9.8531)	0.95 (-0.14,2.05)
Luxembourg	1.02 (0.14,3.56)	0.1617 (0.0227,0.5706)	6.03 (4.59,7.5)
Madagascar	1590.59 (273.05,5119.53)	9.0423 (1.5329,29.3159)	6.3 (4.14,8.5)
Malawi	929.5 (191.14,2669.85)	6.9827 (1.4463,20.6632)	5.53 (2.39,8.77)
Malaysia	161.08 (37.03,482.8)	0.5134 (0.1179,1.5297)	-0.69 (-3.09,1.76)
Maldives	0.98 (0.12,3.66)	0.2154 (0.0265,0.8148)	-8.49 (-10.49,-6.45)
Mali	736.3 (115.58,2146.82)	5.7495 (0.8931,16.221)	0.97 (-0.24,2.19)
Malta	0.59 (0.07,2.29)	0.1543 (0.0172,0.5867)	1.96 (-0.36,4.33)
Marshall Islands	1.86 (0.23,7.67)	3.9551 (0.4899,16.088)	4.62 (2.08,7.23)
Mauritania	59.71 (10.68,173.9)	2.1053 (0.3753,6.2216)	-0.61 (-2,0.81)
Mauritius	0.7 (0.12,2.15)	0.0467 (0.0083,0.1418)	-1.63 (-3.7,0.48)
Mexico	670.63 (125.97,2000.48)	0.5007 (0.0944,1.4892)	3.89 (1.25,6.59)
Moldova	899.81 (529.39,1336.54)	21.0606 (12.4716,31.1598)	7.45 (5.46,9.48)
Monaco	0.03 (0,0.12)	0.0576 (0.0075,0.2179)	0.13 (-0.23,0.5)
Mongolia	311.33 (74.74,795.91)	9.523 (2.2971,24.2809)	7.79 (6.22,9.38)
Montenegro	0.37 (0.06,1.27)	0.0481 (0.0073,0.1624)	-0.14 (-2.38,2.15)
Morocco	543.82 (141.01,1419.64)	1.528 (0.3955,4.0052)	3.36 (0.86,5.94)
Mozambique	4945 (1325.58,12137.81)	25.9126 (6.9321,60.7458)	4.02 (2.35,5.71)
Myanmar	4751.43 (1538.49,10644.81)	8.9861 (2.9281,20.1287)	1.87 (-0.11,3.89)
Namibia	535.29 (184.45,1172.07)	23.2795 (8.2493,49.7963)	10.07 (7.48,12.71)
Nauru	0.04 (0,0.2)	0.468 (0.0453,2.1789)	-0.88 (-2.4,0.67)
Nepal	2172.68 (513.6,5429.19)	8.0797 (1.9212,20.1271)	-0.09 (-1.04,0.88)
Netherlands	14.05 (4.11,34.1)	0.0788 (0.0231,0.1863)	2.76 (2.15,3.38)
New Zealand	5.37 (1.08,15.14)	0.1023 (0.0205,0.2939)	1.54 (1.04,2.04)
Nicaragua	35.44 (5.89,123.92)	0.5808 (0.0954,2.0557)	-1.52 (-2.89,-0.12)
Niger	846.4 (146.44,2348.76)	6.0361 (0.9994,16.9956)	0.75 (-0.52,2.04)
Nigeria	12139.31 (3268.21,33977.05)	8.2431 (2.2226,23.8695)	5.14 (2.44,7.91)
Niue	0 (0,0.01)	0.0937 (0.01,0.3894)	-1.28 (-2.56,0.01)
North Korea	3709.45 (1070.56,8579.27)	12.316 (3.5877,28.6568)	5.84 (3.04,8.72)
North Macedonia	4.06 (1.08,10.71)	0.147 (0.0388,0.3835)	0.04 (-1.52,1.63)
Northern Mariana Islands	0.35 (0.05,1.48)	0.6774 (0.0928,2.8616)	-1.25 (-3.77,1.33)

Norway	6.07 (1.58,17.14)	0.0966 (0.0251,0.2647)	-1.37 (-2.09,-0.64)
Oman	6.17 (1.11,19.34)	0.1876 (0.035,0.5762)	-4.9 (-6.14,-3.63)
Pakistan	21140.65 (6206.34,51957.76)	11.5427 (3.3331,27.6808)	7.74 (4.84,10.73)
Palau	0.02 (0,0.1)	0.1199 (0.0117,0.5291)	-0.6 (-1.83,0.63)
Palestine	7.84 (0.99,28.4)	0.2065 (0.0256,0.7482)	0.9 (-0.82,2.66)
Panama	27.48 (4.68,83.41)	0.6277 (0.1067,1.9035)	0.43 (-1.77,2.67)
Papua New Guinea	442.07 (129.87,1016.01)	6.0972 (1.7854,14.0963)	11.84 (10.28,13.42)
Paraguay	61.12 (8.17,198.58)	0.8812 (0.1181,2.8271)	1.58 (-0.56,3.77)
Peru	2017.76 (835.93,4370.86)	5.4708 (2.2646,11.8527)	-0.05 (-1.56,1.5)
Philippines	9273.31 (2493.73,25024.72)	9.0104 (2.4435,24.2477)	8.1 (5.79,10.46)
Poland	46.53 (12.56,124.76)	0.0897 (0.0242,0.2356)	-4.53 (-5.78,-3.26)
Portugal	19.83 (3.65,63.41)	0.1546 (0.0276,0.4821)	-5.03 (-5.78,-4.29)
Puerto Rico	0.9 (0.16,2.9)	0.0218 (0.0041,0.0728)	-2.02 (-3.79,-0.22)
Qatar	14.21 (2.85,44.39)	0.6539 (0.119,2.089)	0.7 (-0.28,1.69)
Romania	253.2 (75.99,569.17)	1.1271 (0.3385,2.5517)	0.01 (-1.06,1.1)
Russia	28255.81 (15084.46,44579.4)	17.2252 (8.9092,26.7493)	4.18 (1.45,6.98)
Rwanda	643.82 (211.26,1500.74)	7.0809 (2.2603,16.4561)	4.89 (1.48,8.41)
Saint Kitts and Nevis	0.02 (0,0.09)	0.0363 (0.005,0.1386)	-6.27 (-7.42,-5.11)
Saint Lucia	0.08 (0.01,0.31)	0.0409 (0.006,0.1507)	-10.3 (-11.78,-8.8)
Saint Vincent and the Grenadines	0.07 (0.01,0.25)	0.0594 (0.008,0.2049)	-9.51 (-10.77,-8.24)
Samoa	0.06 (0.01,0.21)	0.0312 (0.0037,0.1179)	-4.2 (-6.22,-2.14)
San Marino	0.01 (0,0.04)	0.0242 (0.004,0.0915)	0.09 (-0.3,0.48)
São Tomé and Príncipe	4.37 (0.66,13.89)	2.8849 (0.4181,9.0548)	2.51 (1.05,3.98)
Saudi Arabia	368.4 (72.65,1268.39)	1.1365 (0.2254,3.9016)	2.61 (-0.05,5.34)
Senegal	388.85 (98.96,991.53)	3.6824 (0.9434,9.5085)	3.28 (0.93,5.69)
Serbia	8.85 (2.13,23.36)	0.0751 (0.0177,0.1979)	-0.92 (-2.03,0.21)
Seychelles	0.07 (0.01,0.26)	0.0588 (0.0059,0.225)	-4.08 (-6.41,-1.69)
Sierra Leone	357.2 (65.9,1100.08)	5.8404 (1.0844,17.6524)	0.78 (-0.28,1.87)
Singapore	23.57 (7.44,58.48)	0.3144 (0.0984,0.7773)	0.71 (0.27,1.15)
Slovakia	2.21 (0.34,6.59)	0.0308 (0.0048,0.0918)	-3.51 (-4.71,-2.3)
Slovenia	0.11 (0.02,0.39)	0.0039 (6e-04,0.0139)	-11.85 (-13.51,-10.16)
Solomon Islands	1.22 (0.1,5.14)	0.2349 (0.0193,0.9836)	-0.73 (-2,0.55)
Somalia	5666.92 (1397.02,16612.1)	57.2546 (14.1225,169.5581)	11.42 (7.92,15.03)
South Africa	8095.11 (2257.87,20722.21)	13.9024 (3.8763,35.2994)	3.51 (1.76,5.28)
South Korea	366.43 (52.78,1416.27)	0.4724 (0.0685,1.8396)	-4.6 (-6.25,-2.93)
South Sudan	1297.67 (249.84,3903.63)	20.7975 (4.0662,61.6932)	6.81 (4.7,8.97)
Spain	36.9 (5.34,146.15)	0.0724 (0.0106,0.2836)	-3.51 (-4.3,-2.72)
Sri Lanka	39.2 (4.42,156.75)	0.1572 (0.0176,0.6288)	-2.04 (-3.81,-0.24)
Sudan	327.16 (44.07,1274.3)	0.9967 (0.1346,3.895)	0.08 (-1.93,2.13)
Suriname	0.31 (0.05,1.17)	0.0521 (0.0081,0.195)	-5.88 (-6.93,-4.82)
Sweden	21.06 (6.43,52.05)	0.2051 (0.0633,0.4991)	5.12 (4.33,5.92)
Switzerland	16.75 (5.45,41.98)	0.1928 (0.0624,0.4881)	4.29 (3.72,4.88)
Syria	34.38 (4.35,124.85)	0.2559 (0.0319,0.9358)	-0.66 (-3,1.74)
Taiwan (province of China)	203.97 (29.87,736.62)	0.6527 (0.0957,2.3409)	-5.43 (-7.08,-3.75)

Tajikistan	751.79 (291,1440.92)	8.0466 (3.1232,15.451)	11.05 (7.35,14.87)
Tanzania	2188.72 (380.87,7538.16)	5.4673 (0.9408,19.3656)	4.36 (1.94,6.83)
Thailand	2329.38 (544.58,6097.41)	2.7124 (0.6433,7.0652)	0.28 (-0.44,1)
The Bahamas	0.55 (0.08,2.1)	0.1355 (0.0182,0.5096)	4.16 (2.99,5.35)
The Gambia	82.15 (13.06,243.49)	5.2406 (0.8471,15.5273)	0.7 (-0.59,2.01)
Timor-Leste	16.88 (2.08,72.96)	1.5958 (0.1935,6.7882)	-4.43 (-6.4,-2.43)
Togo	330.81 (49.61,1078.43)	5.8473 (0.8716,18.8772)	1.75 (0.49,3.02)
Tokelau	0 (0,0)	0.0933 (0.01,0.3487)	-2.44 (-3.79,-1.07)
Tonga	0.09 (0.01,0.48)	0.1021 (0.0109,0.5148)	-0.9 (-2.22,0.43)
Trinidad and Tobago	0.67 (0.08,2.36)	0.0428 (0.0053,0.1463)	-4.99 (-6.01,-3.96)
Tunisia	38.16 (10.68,106.45)	0.3055 (0.0873,0.856)	0.17 (-2.19,2.59)
Türkiye	427.79 (154.21,894.28)	0.479 (0.1727,1.0005)	2.2 (-0.49,4.96)
Turkmenistan	406.96 (126.6,873.18)	7.9167 (2.4734,16.9165)	11.09 (7.5,14.8)
Tuvalu	0.08 (0.01,0.34)	0.6696 (0.065,2.9824)	-1.86 (-3.04,-0.67)
Uganda	2706.47 (671.95,7295.38)	9.2468 (2.2914,24.017)	7.59 (4.46,10.81)
UK	58.98 (15.21,157.13)	0.0913 (0.0234,0.2443)	-0.25 (-0.65,0.15)
Ukraine	9218.13 (4219.17,16083.55)	18.198 (8.1018,31.2927)	6.87 (4.73,9.05)
United Arab Emirates	9.19 (1.24,35.61)	0.1856 (0.0244,0.7355)	0.71 (-1.19,2.65)
Uruguay	2.55 (0.47,8.52)	0.0683 (0.0125,0.2291)	1.58 (0.77,2.4)
USA	120.63 (39.96,329.73)	0.0309 (0.0102,0.0838)	-4.62 (-5.37,-3.87)
Uzbekistan	3965.28 (1280.36,7921.7)	12.2161 (3.9661,24.3265)	14.15 (10.23,18.21)
Vanuatu	0.33 (0.03,1.34)	0.1437 (0.0152,0.5849)	2.88 (2.02,3.76)
Venezuela	117.95 (19.03,400.87)	0.4236 (0.0688,1.4545)	0.55 (-1.23,2.36)
Viet Nam	5724.7 (1449.31,14454.22)	5.6579 (1.4303,14.3608)	2.04 (0.86,3.24)
Virgin Islands	0.02 (0,0.09)	0.0191 (0.0027,0.0758)	-6.42 (-7.63,-5.2)
Yemen	143.34 (27.26,505.1)	0.6382 (0.1188,2.2634)	0.81 (-1.41,3.08)
Zambia	1453.4 (239.24,4394.92)	9.2787 (1.579,28.3983)	1.59 (-0.52,3.75)
Zimbabwe	2418.23 (379.38,7832.78)	20.8042 (3.2294,69.8242)	8.63 (7.02,10.27)

Abbreviations: MDR-TB=multidrug-resistant tuberculosis. XDR-TB=extensively drug-resistant tuberculosis. ASR=age-standardized rate. EAPC=estimated annual percentage change.

Table S7 The death of cases and ASR of MDR-TB in 2021, and the trends from baseline to 2021 by countries.

Location	Deaths_Number_2021	Deaths_ASR_2021	EAPC
Afghanistan	710.24 (97.2,2567.19)	5.1479 (0.6988,18.5703)	5.14 (2.02,8.36)
Albania	0.48 (0.06,1.82)	0.0126 (0.0017,0.0473)	-4.25 (-5.52,-2.97)
Algeria	64.33 (5.86,241.98)	0.2119 (0.0195,0.817)	-3.85 (-4.23,-3.47)
American Samoa	0.01 (0,0.03)	0.0148 (0.0013,0.0623)	-2.62 (-3.98,-1.24)
Andorra	0 (0,0)	3e-04 (0,0.0012)	-4.67 (-5.09,-4.23)
Angola	664.33 (86.9,2094.5)	4.2721 (0.5604,13.2784)	0.93 (-0.55,2.42)
Antigua and Barbuda	0 (0,0.01)	0.0037 (4e-04,0.0144)	-9.61 (-10.72,-8.5)
Argentina	20.55 (2.7,79.46)	0.0383 (0.005,0.1476)	-3.19 (-4.69,-1.66)

Armenia	8.1 (1.86,16.87)	0.2145 (0.0491,0.4479)	5.87 (1.48,10.46)
Australia	4.89 (1.27,12.91)	0.0101 (0.0026,0.0265)	1.6 (0.88,2.33)
Austria	3.19 (0.75,8.12)	0.0162 (0.0038,0.0414)	0.5 (-0.41,1.41)
Azerbaijan	110.75 (35.16,230.7)	1.0089 (0.3257,2.0677)	8.95 (4.93,13.12)
Bahrain	0.7 (0.09,2.59)	0.1113 (0.0138,0.4146)	0.93 (-1.6,3.53)
Bangladesh	2447.98 (562.5,6425.51)	1.8473 (0.4206,4.8205)	3.14 (0.07,6.3)
Barbados	0.01 (0,0.05)	0.0023 (2e-04,0.0115)	-12.56 (-14.25,-10.83)
Belarus	97.8 (57.26,140.77)	0.7203 (0.4204,1.0378)	3.03 (-0.04,6.2)
Belgium	2.19 (0.44,6.42)	0.0085 (0.0017,0.0247)	-4.94 (-5.74,-4.14)
Belize	0.11 (0.01,0.45)	0.0325 (0.0029,0.133)	-7.5 (-8.8,-6.18)
Benin	81.08 (12.31,279.71)	1.3058 (0.2055,4.2637)	2.55 (0.8,4.33)
Bermuda	0 (0,0)	3e-04 (0,0.0016)	-11.68 (-13.26,-10.08)
Bhutan	8.96 (1.11,29.34)	1.4354 (0.1794,4.6888)	-0.35 (-1.82,1.14)
Bolivia	139.3 (19.99,449.47)	1.472 (0.2144,4.7315)	-3.08 (-4.25,-1.9)
Bosnia and Herzegovina	0.95 (0.17,3.63)	0.0163 (0.0029,0.0627)	-5.06 (-5.68,-4.44)
Botswana	74.44 (11.48,224.57)	4.1749 (0.6398,12.5519)	6.32 (4.93,7.73)
Brazil	326.22 (55.16,988.99)	0.1313 (0.0222,0.3965)	4.68 (1.7,7.75)
Brunei	0.09 (0.01,0.33)	0.0344 (0.0033,0.1275)	-4.26 (-5.56,-2.94)
Bulgaria	3.3 (0.61,10.39)	0.0272 (0.005,0.0858)	-0.64 (-2.78,1.54)
Burkina Faso	347.25 (57.31,1011.52)	3.1277 (0.5113,9.1912)	2.61 (1.41,3.83)
Burundi	489.85 (69.7,1668.72)	8.6201 (1.2259,29.5752)	4.3 (1.82,6.84)
Cabo Verde	4.15 (0.55,14.12)	0.918 (0.1204,3.1506)	0.19 (-1.22,1.62)
Cambodia	99.48 (12.02,371.98)	0.8156 (0.0985,3.0773)	-2.55 (-5.72,0.72)
Cameroon	250.5 (32.91,828.56)	1.7406 (0.2252,5.7149)	0.31 (-1.15,1.78)
Canada	3.01 (0.72,8.2)	0.0041 (0.001,0.0114)	-5.49 (-5.94,-5.04)
Central African Republic	241.53 (28.81,852.42)	8.2629 (1.0129,29.9267)	0.89 (-0.33,2.13)
Chad	349.74 (54.57,1106.03)	4.5474 (0.7094,14.1486)	2.04 (0.48,3.62)
Chile	9.73 (2.06,27.08)	0.0393 (0.0083,0.1094)	-2.26 (-3.03,-1.49)
China	2876.38 (479.78,8389.16)	0.1469 (0.0246,0.4272)	-10.03 (-11.23,-8.82)
Colombia	84.65 (13.75,247.56)	0.1555 (0.0253,0.4552)	1.28 (-0.2,2.79)
Comoros	46.08 (5.72,135.29)	9.6647 (1.1967,28.0117)	6.56 (4.23,8.95)
Congo (Brazzaville)	83.57 (9.24,333.67)	2.6917 (0.3092,10.5311)	-0.14 (-1.53,1.27)
Cook Islands	0 (0,0.01)	0.0063 (6e-04,0.0266)	-4.51 (-5.73,-3.28)
Costa Rica	2.16 (0.27,7.53)	0.0395 (0.0049,0.138)	-0.81 (-3.39,1.84)
Côte d'Ivoire	336.01 (49.94,1104.4)	2.5893 (0.3783,8.276)	-3.45 (-4.59,-2.3)
Croatia	0.34 (0.06,1.12)	0.004 (7e-04,0.0133)	-8.42 (-9.54,-7.28)
Cuba	1.46 (0.25,4.45)	0.0081 (0.0014,0.0246)	-5.71 (-6.5,-4.92)
Cyprus	0.2 (0.03,0.62)	0.0117 (0.002,0.0367)	-4.44 (-6.28,-2.56)
Czechia	1.07 (0.24,2.94)	0.0054 (0.0012,0.0148)	-4.73 (-5.53,-3.92)
Denmark	0.61 (0.11,1.84)	0.005 (9e-04,0.0151)	-0.67 (-1.18,-0.15)
Djibouti	58.99 (15.85,156.11)	9.2221 (2.4597,24.7328)	11.08 (7.45,14.84)
Dominica	0.03 (0,0.11)	0.0362 (0.0038,0.1386)	-8.55 (-9.81,-7.28)
Dominican Republic	6.83 (0.79,29.38)	0.0648 (0.0075,0.2806)	-6.01 (-7.26,-4.73)
DR Congo	2439.58 (345.86,9804.81)	5.4767 (0.7668,21.1848)	2.48 (1.15,3.82)

Ecuador	44.05 (6.61,140.71)	0.264 (0.0394,0.84)	-3.39 (-5.78,-0.93)
Egypt	81.09 (14.23,248.42)	0.1355 (0.0236,0.4074)	2.26 (-0.19,4.78)
El Salvador	8 (0.94,25.87)	0.1266 (0.0148,0.4088)	-3.69 (-5.7,-1.63)
Equatorial Guinea	10.83 (1.44,41.14)	1.6567 (0.2244,6.0985)	-3.97 (-5.16,-2.77)
Eritrea	384.67 (51.45,1258.47)	12.0639 (1.6411,38.935)	6.05 (3.62,8.55)
Estonia	4.96 (2.11,8.87)	0.2418 (0.1033,0.4286)	-4.76 (-6.54,-2.96)
Eswatini	123.32 (24.91,316.12)	17.1916 (3.5464,44.6467)	8.96 (6,12)
Ethiopia	2092.61 (312.29,6803.61)	4.3344 (0.6553,14.0229)	1.64 (-0.93,4.27)
Federated States of Micronesia	0.08 (0.01,0.36)	0.0975 (0.0077,0.4491)	-2.36 (-3.63,-1.07)
Fiji	0.17 (0.01,0.79)	0.0224 (0.0019,0.106)	1.35 (0.27,2.45)
Finland	2.87 (0.6,8.08)	0.0207 (0.0044,0.0581)	2.15 (1.59,2.72)
France	26.78 (6.17,78.32)	0.015 (0.0035,0.0441)	-3.89 (-4.75,-3.01)
Gabon	26.72 (3.43,97.43)	2.3862 (0.3067,8.4887)	1.9 (0.59,3.23)
Georgia	29.4 (12.28,50.42)	0.5919 (0.2487,1.0075)	5.25 (2.01,8.59)
Germany	33.34 (8.71,80.54)	0.0159 (0.0041,0.0387)	-1.76 (-2.19,-1.34)
Ghana	466.18 (55.81,1661.05)	2.7806 (0.3332,9.7388)	1.4 (0.35,2.45)
Greece	5.37 (0.88,17.5)	0.0201 (0.0033,0.0654)	1.5 (-0.47,3.5)
Greenland	0.1 (0.01,0.34)	0.1821 (0.0218,0.6314)	-4.59 (-5.23,-3.95)
Grenada	0.01 (0,0.02)	0.005 (5e-04,0.0234)	-6.7 (-7.75,-5.64)
Guam	0.07 (0.01,0.29)	0.0351 (0.0033,0.1481)	-1.03 (-3.23,1.21)
Guatemala	24.23 (3.54,73.93)	0.2027 (0.0297,0.6206)	-4.79 (-6.81,-2.72)
Guinea	147.72 (19.38,545.89)	2.308 (0.2997,8.2569)	1.04 (-0.25,2.35)
Guinea-Bissau	34.64 (3.87,120.44)	4.3482 (0.498,15.4338)	0.11 (-1.34,1.59)
Guyana	0.62 (0.06,2.62)	0.0895 (0.0088,0.376)	-3.3 (-4.49,-2.1)
Haiti	12.71 (1.11,55.06)	0.1429 (0.0119,0.6435)	-4.88 (-5.75,-4.01)
Honduras	33.64 (4.7,100.93)	0.5142 (0.0712,1.5613)	2.3 (-0.11,4.76)
Hungary	1.32 (0.21,4.36)	0.0076 (0.0012,0.0252)	-6.66 (-8.73,-4.55)
Iceland	0.02 (0,0.08)	0.0037 (5e-04,0.0127)	-3.89 (-6.45,-1.25)
India	53333.12 (10384.24,133519.76)	4.3893 (0.8589,10.9695)	5.84 (3.06,8.7)
Indonesia	692.31 (68.42,2951.55)	0.3257 (0.033,1.4058)	-5.92 (-8.24,-3.55)
Iran	33.37 (7.51,89.63)	0.0455 (0.0103,0.122)	2.71 (0.38,5.09)
Iraq	57.65 (12.73,155.16)	0.2495 (0.055,0.6685)	2.8 (-0.12,5.8)
Ireland	0.65 (0.09,2.16)	0.008 (0.0011,0.0265)	-3.83 (-4.75,-2.9)
Israel	2.82 (0.6,7.77)	0.0216 (0.0046,0.0593)	-5.89 (-6.94,-4.83)
Italy	14.32 (3.18,37.56)	0.0089 (0.002,0.023)	-4.76 (-6.24,-3.25)
Jamaica	0.42 (0.05,1.77)	0.0135 (0.0016,0.0568)	-2.31 (-3.44,-1.16)
Japan	62.28 (6.49,245.45)	0.0108 (0.0011,0.0438)	-8.26 (-9.6,-6.89)
Jordan	3.92 (0.56,13.21)	0.0558 (0.0078,0.1917)	0.32 (-2.31,3.02)
Kazakhstan	167.93 (73.92,281.46)	0.8653 (0.3816,1.4452)	-0.07 (-4.42,4.47)
Kenya	906.95 (154.23,2666.11)	3.7431 (0.6511,10.8014)	7.38 (3.96,10.92)
Kiribati	0.5 (0.04,2.3)	0.6112 (0.0499,2.7836)	-1.36 (-2.84,0.14)
Kuwait	1.59 (0.27,5.15)	0.0599 (0.0101,0.1936)	0.83 (-1.53,3.23)
Kyrgyzstan	102.27 (37.27,183.02)	1.7042 (0.622,3.0494)	9.83 (4.95,14.94)
Laos	17.15 (1.56,72.78)	0.3517 (0.032,1.5008)	-7.37 (-9.65,-5.03)

Latvia	7.11 (2.45,14.08)	0.2399 (0.0827,0.4758)	-6.82 (-7.9,-5.72)
Lebanon	2.11 (0.22,7.75)	0.0338 (0.0036,0.1243)	-1.79 (-3.87,0.33)
Lesotho	265.86 (61.19,748.48)	20.701 (4.7425,58.9463)	12.6 (9.67,15.61)
Liberia	32.22 (3.97,118.71)	1.3301 (0.167,4.8336)	-1.57 (-2.99,-0.13)
Libya	6.64 (0.65,25.94)	0.1297 (0.0125,0.5017)	1.75 (-0.19,3.72)
Lithuania	24.08 (10.17,40.82)	0.5769 (0.2437,0.9704)	-1.54 (-3.17,0.12)
Luxembourg	0.06 (0.01,0.21)	0.0053 (6e-04,0.0182)	0.87 (-0.46,2.22)
Madagascar	624.53 (81.81,2220.72)	4.8549 (0.6604,17.2987)	6.78 (4.27,9.34)
Malawi	318.31 (54.09,1065.34)	3.8828 (0.6667,12.5626)	5.44 (2.31,8.68)
Malaysia	36.07 (6.71,110.96)	0.1325 (0.0247,0.4015)	-1.1 (-4,1.89)
Maldives	0.17 (0.01,0.7)	0.0496 (0.0044,0.2038)	-11.08 (-13.09,-9.02)
Mali	273.39 (34.05,843.17)	3.135 (0.4068,9.815)	0.44 (-0.74,1.64)
Malta	0.01 (0,0.05)	0.0011 (1e-04,0.0047)	-4.21 (-6.65,-1.7)
Marshall Islands	0.68 (0.07,2.91)	1.8147 (0.1729,7.8154)	4.19 (1.48,6.97)
Mauritania	23.54 (3.05,81.89)	1.0801 (0.1341,3.6966)	-1.4 (-2.8,0.03)
Mauritius	0.11 (0.02,0.4)	0.0066 (9e-04,0.0235)	-3.53 (-5.97,-1.03)
Mexico	144.08 (24.92,443.78)	0.1113 (0.0194,0.3427)	0.71 (-1.86,3.34)
Moldova	58.23 (34.39,81.36)	1.1272 (0.6668,1.5728)	3.52 (0.54,6.58)
Monaco	0.01 (0,0.06)	0.0135 (0.0016,0.056)	-1.67 (-1.97,-1.36)
Mongolia	69.49 (14.33,172.87)	2.1892 (0.4464,5.4532)	4.7 (2.85,6.58)
Montenegro	0.07 (0.01,0.3)	0.0084 (0.0011,0.0331)	-1.26 (-3.61,1.14)
Morocco	144.32 (25.04,426.45)	0.4549 (0.0787,1.357)	2.34 (-0.27,5.01)
Mozambique	2023.89 (460.63,5435.57)	15.6098 (3.5539,41.4206)	4.2 (2.39,6.04)
Myanmar	1652.34 (393.36,4356.2)	3.4906 (0.8302,9.1585)	0.91 (-1.5,3.37)
Namibia	123.25 (34.09,313.77)	7.8648 (2.1946,19.8937)	8.93 (5.93,12.02)
Nauru	0.01 (0,0.07)	0.1713 (0.0126,0.8735)	-2.14 (-3.87,-0.38)
Nepal	715.69 (143.47,1981.11)	3.1052 (0.6234,8.5768)	-1.58 (-2.36,-0.78)
Netherlands	3.08 (0.68,7.99)	0.0082 (0.0018,0.0213)	-0.78 (-1.4,-0.16)
New Zealand	0.18 (0.03,0.6)	0.0022 (4e-04,0.007)	-5.42 (-6.82,-4)
Nicaragua	8.93 (1.2,30.49)	0.1693 (0.0226,0.5782)	-3.42 (-4.82,-1.99)
Niger	339.78 (42.8,1126.08)	3.2807 (0.4218,11.2756)	0.17 (-1.11,1.45)
Nigeria	3882.11 (798.01,10624.71)	3.6049 (0.7406,9.9624)	4.91 (2.17,7.72)
Niue	0 (0,0)	0.0318 (0.003,0.1528)	-2.46 (-3.87,-1.03)
North Korea	999.73 (221.34,2867.59)	3.1314 (0.6912,8.9216)	6.27 (2.88,9.78)
North Macedonia	0.76 (0.13,2.35)	0.0286 (0.005,0.0875)	-2.45 (-4.14,-0.73)
Northern Mariana Islands	0.07 (0.01,0.3)	0.1555 (0.0154,0.6476)	-3.47 (-5.71,-1.17)
Norway	1.83 (0.38,5.59)	0.0157 (0.0032,0.0478)	-3.4 (-4.13,-2.66)
Oman	0.76 (0.1,2.65)	0.0394 (0.0048,0.1377)	-6.06 (-7.23,-4.88)
Pakistan	7649.7 (1824.07,21775.14)	5.2959 (1.2466,15.077)	7.85 (4.58,11.21)
Palau	0.01 (0,0.04)	0.0429 (0.0037,0.1922)	-1.58 (-2.87,-0.28)
Palestine	1.23 (0.14,5.22)	0.0527 (0.0055,0.2239)	-0.32 (-2.2,1.59)
Panama	6.74 (0.96,21.9)	0.1527 (0.0219,0.4965)	-1.8 (-3.98,0.44)
Papua New Guinea	185.13 (39.26,487.6)	3.3601 (0.7081,8.911)	11.25 (9.54,12.98)
Paraguay	15.47 (1.91,50.35)	0.2515 (0.0313,0.822)	1.21 (-0.95,3.41)

Peru	364.56 (111.57,874.79)	1.0416 (0.317,2.5085)	-2.98 (-4.37,-1.58)
Philippines	1402.37 (304.42,3778.48)	1.6479 (0.3555,4.4033)	6.12 (3.24,9.09)
Poland	6.87 (1.52,18.73)	0.0108 (0.0024,0.0292)	-7.41 (-8.62,-6.19)
Portugal	6.27 (0.91,19.7)	0.0238 (0.0034,0.0747)	-6.84 (-7.67,-6)
Puerto Rico	0.31 (0.05,1.04)	0.0052 (8e-04,0.0173)	-4.32 (-6.34,-2.27)
Qatar	1.07 (0.15,3.72)	0.1211 (0.0173,0.4322)	-2.47 (-3.66,-1.28)
Romania	43.97 (10.48,109.95)	0.1501 (0.0359,0.3756)	-2.76 (-4.11,-1.39)
Russia	1720.72 (938.76,2586.4)	0.8734 (0.4747,1.3163)	1.54 (-1.64,4.82)
Rwanda	255.03 (59.12,652.92)	3.8869 (0.8957,9.9572)	3.74 (0.19,7.42)
Saint Kitts and Nevis	0.01 (0,0.03)	0.0113 (0.0013,0.0455)	-7.87 (-8.93,-6.8)
Saint Lucia	0.03 (0,0.12)	0.0137 (0.0019,0.0542)	-11.89 (-13.25,-10.52)
Saint Vincent and the Grenadines	0.02 (0,0.07)	0.0133 (0.0016,0.0497)	-11.35 (-12.44,-10.26)
Samoa	0.02 (0,0.09)	0.013 (0.0011,0.0598)	-4.79 (-6.89,-2.64)
San Marino	0 (0,0.01)	0.0034 (4e-04,0.0121)	-2.11 (-2.37,-1.84)
São Tomé and Príncipe	1.35 (0.16,4.38)	1.2018 (0.146,3.8327)	1.48 (-0.1,3.09)
Saudi Arabia	62.12 (9.91,208.85)	0.3174 (0.0516,1.0308)	1.06 (-1.74,3.94)
Senegal	149.4 (31.12,432.84)	1.859 (0.3894,5.3298)	3.74 (1.34,6.2)
Serbia	2.02 (0.39,6.1)	0.0131 (0.0025,0.0395)	-4.02 (-5.36,-2.65)
Seychelles	0.02 (0,0.07)	0.0138 (0.0012,0.0615)	-5.12 (-7.93,-2.22)
Sierra Leone	117.73 (16.59,399.63)	2.5687 (0.3763,8.8843)	1.1 (0.01,2.2)
Singapore	1.34 (0.33,3.94)	0.0165 (0.004,0.0483)	-4.46 (-5.2,-3.72)
Slovakia	0.44 (0.06,1.57)	0.0051 (7e-04,0.0181)	-6.43 (-7.46,-5.38)
Slovenia	0.01 (0,0.05)	3e-04 (0,0.0012)	-14.39 (-16.3,-12.44)
Solomon Islands	0.47 (0.04,1.95)	0.1144 (0.0087,0.4722)	-1.34 (-2.68,0.01)
Somalia	2750.82 (558.92,8081.86)	37.5814 (7.6297,109.5905)	12 (8.28,15.86)
South Africa	1371.73 (277.02,3703.35)	2.7511 (0.5555,7.3589)	1.84 (-0.34,4.06)
South Korea	64.54 (7.93,259.6)	0.0716 (0.0088,0.2876)	-8.73 (-10.37,-7.06)
South Sudan	502.7 (73.12,1532.27)	10.3046 (1.5578,33.4503)	6.89 (4.57,9.27)
Spain	8.07 (1,29.96)	0.0071 (9e-04,0.0266)	-6.77 (-7.46,-6.08)
Sri Lanka	6.94 (0.79,31.1)	0.0271 (0.0031,0.1214)	-4.73 (-6.81,-2.6)
Sudan	57.88 (6.93,212.81)	0.2582 (0.031,0.9434)	-1.44 (-3.52,0.68)
Suriname	0.08 (0.01,0.35)	0.0132 (0.0014,0.0567)	-7.27 (-8.3,-6.24)
Sweden	3.22 (0.83,8.26)	0.0119 (0.0031,0.0304)	-1.37 (-2.35,-0.37)
Switzerland	2.21 (0.56,5.57)	0.0106 (0.0027,0.0265)	-0.98 (-1.66,-0.29)
Syria	5.39 (0.53,19.9)	0.0474 (0.0047,0.1749)	-2.36 (-4.88,0.23)
Taiwan (province of China)	28.06 (3.48,108.96)	0.0664 (0.0082,0.258)	-9.89 (-11.48,-8.27)
Tajikistan	158.39 (56.92,305.16)	1.8788 (0.6772,3.6434)	9.4 (5.43,13.52)
Tanzania	851.14 (127.62,3091.54)	2.9156 (0.4288,10.5344)	5.22 (2.63,7.87)
Thailand	452.1 (77.72,1273.42)	0.4394 (0.0753,1.2462)	-3.3 (-4.07,-2.52)
The Bahamas	0.16 (0.02,0.6)	0.0387 (0.004,0.147)	2.32 (1.04,3.61)
The Gambia	26.42 (3.77,95.94)	2.4501 (0.3499,8.6028)	0.77 (-0.59,2.14)
Timor-Leste	6.29 (0.52,25.55)	0.7083 (0.0602,2.8371)	-4.86 (-6.94,-2.74)
Togo	115.1 (14.25,411.04)	2.8526 (0.3531,10.3185)	1.64 (0.27,3.03)
Tokelau	0 (0,0)	0.0328 (0.0025,0.1467)	-4.01 (-5.39,-2.6)

Tonga	0.03 (0,0.15)	0.0362 (0.0034,0.1876)	-1.43 (-2.83,-0.01)
Trinidad and Tobago	0.16 (0.02,0.63)	0.0088 (0.0011,0.0354)	-7.79 (-8.85,-6.71)
Tunisia	7.12 (1.44,22.89)	0.0584 (0.0119,0.1889)	-0.01 (-2.45,2.49)
Türkiye	77.63 (22.54,187.13)	0.0893 (0.026,0.2141)	-0.64 (-3.26,2.05)
Turkmenistan	84.74 (26.01,167.84)	1.6909 (0.5199,3.3478)	9.71 (5.36,14.24)
Tuvalu	0.03 (0,0.13)	0.2657 (0.0189,1.2436)	-3.16 (-4.37,-1.93)
Uganda	870.4 (165.19,2684.96)	4.911 (0.9246,15.2076)	7.27 (3.88,10.78)
UK	7.67 (1.63,21.71)	0.0058 (0.0012,0.0164)	-3.57 (-3.99,-3.15)
Ukraine	857.15 (377.82,1440.46)	1.3755 (0.6087,2.3004)	4.23 (1.43,7.12)
United Arab Emirates	1.43 (0.12,5.86)	0.0765 (0.0075,0.3103)	0.98 (-1,2.99)
Uruguay	0.36 (0.05,1.17)	0.0072 (0.0011,0.0237)	-1.81 (-2.74,-0.87)
USA	23.7 (5.92,71.26)	0.0043 (0.0011,0.013)	-7.8 (-9.06,-6.53)
Uzbekistan	408.46 (142.92,717.35)	1.2715 (0.4462,2.2411)	10.31 (5.69,15.13)
Vanuatu	0.14 (0.01,0.62)	0.0779 (0.0065,0.3415)	2.4 (1.42,3.39)
Venezuela	34.77 (4.53,128.62)	0.1206 (0.0157,0.4465)	-1.32 (-3.15,0.54)
Viet Nam	1455.1 (287.48,3953.8)	1.6215 (0.3251,4.4268)	-0.29 (-1.5,0.95)
Virgin Islands	0.01 (0,0.03)	0.005 (5e-04,0.0221)	-7.54 (-8.71,-6.35)
Yemen	42.53 (6.51,157.69)	0.2754 (0.0422,1.0074)	0.13 (-2.29,2.61)
Zambia	222.23 (28.44,722.47)	2.9496 (0.4053,9.691)	-0.94 (-3.27,1.45)
Zimbabwe	1008.29 (141.58,3260.16)	11.8306 (1.7321,37.8523)	9.86 (7.99,11.76)

Abbreviations: MDR-TB=multidrug-resistant tuberculosis. XDR-TB=extensively drug-resistant tuberculosis. ASR=age-standardized rate. EAPC=estimated annual percentage change.

Table S8 The incidence of cases and ASR of XDR-TB in 2021, and the trends from baseline to 2021 by countries.

location	Incidence_Number_2021	Incidence_ASR_2021	EAPC
Afghanistan	77.78 (10.3,282.85)	0.5182 (0.0678,1.92)	10.86 (7,14.87)
Albania	0.55 (0.1,1.82)	0.0165 (0.0029,0.0549)	5.41 (3.29,7.58)
Algeria	12.46 (1.55,45.11)	0.0347 (0.0043,0.1282)	5.7 (4.41,7.01)
American Samoa	0 (0,0.01)	0.0048 (5e-04,0.0193)	4.79 (3.23,6.37)
Andorra	0 (0,0)	0.0012 (2e-04,0.0045)	4.75 (3.2,6.33)
Angola	15.11 (2.33,42.81)	0.0836 (0.0127,0.2425)	8.49 (6.23,10.79)
Antigua and Barbuda	0.01 (0,0.03)	0.0061 (8e-04,0.0267)	1.09 (-0.29,2.48)
Argentina	13.44 (2.15,49.79)	0.0274 (0.0044,0.1015)	5.91 (3.36,8.52)
Armenia	23.38 (5.44,56.98)	0.7002 (0.1627,1.7365)	15.07 (11.14,19.14)
Australia	5.69 (1.74,13.65)	0.0195 (0.006,0.0468)	15.58 (14.69,16.47)
Austria	2.21 (0.69,5.02)	0.0204 (0.0065,0.048)	12.95 (10.57,15.37)
Azerbaijan	259.45 (78.38,527.87)	2.3024 (0.7053,4.6952)	18.4 (14.52,22.41)
Bahrain	0.2 (0.03,0.78)	0.0184 (0.0029,0.0721)	8.04 (5.11,11.05)
Bangladesh	240.02 (67.74,625.45)	0.1669 (0.0466,0.4378)	8.85 (5.62,12.19)
Barbados	0 (0,0.02)	0.0012 (1e-04,0.0049)	-4.12 (-5.84,-2.38)
Belarus	190.71 (116.62,293.51)	1.7044 (1.0415,2.6197)	11.02 (8.45,13.65)
Belgium	1.92 (0.49,5.08)	0.015 (0.0039,0.0399)	7.19 (5.58,8.83)

Belize	0.03 (0,0.14)	0.0094 (0.0011,0.0384)	0.4 (-1.43,2.27)
Benin	1.83 (0.36,5.33)	0.0253 (0.0049,0.0738)	10.18 (7.12,13.33)
Bermuda	0 (0,0.02)	0.0049 (4e-04,0.0246)	0.77 (-0.94,2.51)
Bhutan	0.92 (0.15,2.77)	0.1347 (0.0227,0.4054)	7.86 (5.76,10.01)
Bolivia	38.69 (6.43,122.34)	0.3829 (0.0636,1.201)	5.22 (3.48,7)
Bosnia and Herzegovina	0.86 (0.19,2.69)	0.0181 (0.004,0.0566)	5.58 (3.94,7.23)
Botswana	2.68 (0.54,7.88)	0.1238 (0.0242,0.3606)	16.56 (13.52,19.67)
Brazil	171.91 (36.1,497.71)	0.0719 (0.015,0.2081)	12.2 (9.21,15.27)
Brunei	0.09 (0.01,0.32)	0.0231 (0.0026,0.0839)	4.92 (3.47,6.38)
Bulgaria	4.78 (1.09,13.43)	0.0579 (0.0138,0.1655)	9.88 (6.96,12.88)
Burkina Faso	7.13 (1.29,19.32)	0.0586 (0.0107,0.1581)	10.38 (8.2,12.6)
Burundi	10.28 (1.7,34.87)	0.1615 (0.027,0.5522)	10.33 (7.64,13.09)
Cabo Verde	0.11 (0.02,0.3)	0.022 (0.0035,0.0616)	7.61 (5.6,9.66)
Cambodia	31.89 (4.92,108.92)	0.2407 (0.037,0.828)	2.34 (-0.81,5.58)
Cameroon	5.96 (1.03,18.13)	0.034 (0.0059,0.1009)	7.79 (5.39,10.23)
Canada	3.09 (0.97,7.64)	0.0068 (0.0021,0.0167)	7.75 (6.56,8.95)
Central African Republic	4.54 (0.76,15.14)	0.1483 (0.0245,0.5022)	8.57 (6.6,10.58)
Chad	7.11 (1.22,20.39)	0.0837 (0.0145,0.2355)	9.46 (6.97,12.01)
Chile	4.34 (1.19,11.13)	0.019 (0.0053,0.049)	7.26 (5.22,9.34)
China	2248.11 (379.91,6971.55)	0.132 (0.0224,0.411)	-0.2 (-1.78,1.41)
Colombia	34.82 (6.72,104.68)	0.0649 (0.0125,0.1959)	10.6 (8.58,12.67)
Comoros	1.12 (0.15,3.45)	0.2085 (0.0278,0.664)	13.46 (11.05,15.93)
Congo (Brazzaville)	1.9 (0.29,6.5)	0.0541 (0.0083,0.1849)	7.54 (5.46,9.65)
Cook Islands	0 (0,0)	0.0032 (4e-04,0.0128)	4.3 (2.77,5.86)
Costa Rica	0.68 (0.11,2.25)	0.0128 (0.002,0.0427)	5.27 (2.69,7.91)
Côte d'Ivoire	8.08 (1.54,24.95)	0.0513 (0.0096,0.157)	3.88 (2.33,5.47)
Croatia	0.31 (0.07,0.96)	0.0049 (0.0012,0.015)	1.12 (-1.18,3.48)
Cuba	1 (0.19,3.05)	0.0076 (0.0015,0.023)	5.68 (4.78,6.6)
Cyprus	0.13 (0.02,0.41)	0.0083 (0.0016,0.0266)	5.07 (1.58,8.67)
Czechia	1.85 (0.54,4.74)	0.0139 (0.0041,0.0364)	6.92 (5.15,8.71)
Denmark	0.55 (0.13,1.53)	0.0084 (0.002,0.023)	12 (10.27,13.75)
Djibouti	1.32 (0.49,3.21)	0.1811 (0.0678,0.4379)	16.12 (12.13,20.25)
Dominica	0.01 (0,0.03)	0.0102 (0.0014,0.0352)	-0.16 (-1.22,0.91)
Dominican Republic	2.04 (0.27,7.91)	0.0188 (0.0025,0.0731)	1.87 (0.12,3.64)
DR Congo	53.39 (9.23,162.3)	0.1056 (0.0182,0.3314)	10.16 (8.04,12.32)
Ecuador	28.11 (4.91,93.28)	0.1576 (0.0274,0.525)	4.32 (1.81,6.89)
Egypt	14.67 (3.45,44.33)	0.0191 (0.0045,0.0578)	8.91 (5.88,12.03)
El Salvador	3.33 (0.54,10.79)	0.0518 (0.0084,0.1682)	3.37 (1.37,5.4)
Equatorial Guinea	0.34 (0.06,1.17)	0.0362 (0.0061,0.1281)	4.37 (2.99,5.77)
Eritrea	8.25 (1.52,23.37)	0.2304 (0.0426,0.6484)	12.18 (9.47,14.95)
Estonia	6.86 (2.97,13.15)	0.4447 (0.1883,0.8526)	4.18 (1.94,6.46)
Eswatini	3.99 (0.95,10.02)	0.4276 (0.0989,1.0905)	16.91 (12.5,21.5)
Ethiopia	52.32 (7.67,167.02)	0.0879 (0.0131,0.2806)	7.75 (4.94,10.62)
Federated States of Micronesia	0.02 (0,0.11)	0.0293 (0.0027,0.1325)	5.27 (3.68,6.89)

Fiji	0.06 (0.01,0.26)	0.0072 (8e-04,0.0318)	10.33 (8.8,11.89)
Finland	1.37 (0.35,3.76)	0.0163 (0.0043,0.0447)	14.78 (12.84,16.75)
France	14.43 (3.92,38.95)	0.0148 (0.0041,0.0392)	7.69 (5.68,9.75)
Gabon	0.68 (0.12,2.21)	0.0515 (0.0093,0.1697)	9.97 (7.88,12.1)
Georgia	80.57 (38.7,137.52)	2.0587 (0.9799,3.577)	16.02 (12.78,19.35)
Germany	24.84 (7.88,57.14)	0.0256 (0.008,0.0589)	11.97 (10.6,13.34)
Ghana	10.61 (1.62,35.49)	0.0544 (0.0083,0.1839)	9.42 (7.45,11.42)
Greece	2.33 (0.45,6.99)	0.0138 (0.0026,0.0409)	12.11 (8.8,15.51)
Greenland	0.12 (0.02,0.42)	0.1955 (0.0279,0.6961)	9.19 (8.16,10.24)
Grenada	0 (0,0.02)	0.0044 (5e-04,0.0182)	3.79 (2.31,5.29)
Guam	0.02 (0,0.1)	0.0134 (0.0016,0.0535)	6.72 (3.26,10.29)
Guatemala	6.49 (1.08,18.9)	0.0508 (0.0084,0.1483)	1.98 (-0.05,4.05)
Guinea	3.06 (0.55,9.13)	0.043 (0.0077,0.1279)	8.76 (6.48,11.1)
Guinea-Bissau	0.7 (0.1,2.42)	0.0793 (0.0116,0.2802)	7.68 (5.23,10.2)
Guyana	0.18 (0.02,0.78)	0.0247 (0.0029,0.1095)	5.24 (3.15,7.38)
Haiti	3.3 (0.46,10.82)	0.0344 (0.0048,0.1152)	3.59 (2.11,5.09)
Honduras	8.73 (1.54,26.74)	0.1227 (0.0219,0.374)	8.6 (5.95,11.32)
Hungary	1.54 (0.31,4.64)	0.0119 (0.0023,0.036)	2.67 (-0.29,5.71)
Iceland	0.02 (0,0.05)	0.0038 (6e-04,0.0124)	5.65 (1.48,9.99)
India	5770.92 (1250.47,13903.36)	0.4377 (0.0957,1.0641)	12.5 (9.4,15.69)
Indonesia	202.23 (27.45,728)	0.0914 (0.0126,0.3249)	-0.39 (-2.93,2.22)
Iran	7.66 (2.37,19.2)	0.0095 (0.0029,0.024)	10.44 (7.61,13.33)
Iraq	10.06 (2.71,25.46)	0.0344 (0.0093,0.0877)	9.09 (5.57,12.72)
Ireland	0.49 (0.09,1.47)	0.0089 (0.0017,0.026)	7.84 (5.71,10.01)
Israel	2.12 (0.55,5.63)	0.0203 (0.0052,0.0542)	4.2 (2.48,5.95)
Italy	13.06 (3.88,33.72)	0.0206 (0.006,0.0529)	6.33 (3.22,9.52)
Jamaica	0.13 (0.02,0.55)	0.0045 (7e-04,0.0182)	6.55 (5.42,7.7)
Japan	27.23 (3.27,102.79)	0.0076 (9e-04,0.0281)	0.46 (-1.01,1.95)
Jordan	0.91 (0.16,3.23)	0.0096 (0.0017,0.0343)	7.21 (4.25,10.25)
Kazakhstan	443.76 (208.77,789.48)	2.3133 (1.0891,4.0858)	9.02 (5.3,12.86)
Kenya	18.86 (4.45,44.1)	0.0701 (0.0168,0.1616)	11.91 (8.01,15.96)
Kiribati	0.14 (0.02,0.62)	0.1648 (0.0187,0.7105)	6.05 (4.04,8.1)
Kuwait	0.47 (0.1,1.31)	0.0114 (0.0026,0.032)	7.71 (5.4,10.07)
Kyrgyzstan	271.22 (89.76,538.04)	4.2479 (1.4111,8.4383)	19.01 (14.93,23.23)
Laos	4.72 (0.64,19.84)	0.0931 (0.0125,0.3875)	-1.95 (-4.41,0.57)
Latvia	10.07 (4.29,18.83)	0.4753 (0.1977,0.8938)	2.43 (1.04,3.83)
Lebanon	0.48 (0.06,1.94)	0.0082 (0.0011,0.0331)	6.35 (4.11,8.65)
Lesotho	5.78 (1.59,14.86)	0.4088 (0.1119,1.0637)	19.17 (15.2,23.28)
Liberia	0.78 (0.12,2.54)	0.0264 (0.004,0.088)	4.98 (3.29,6.7)
Libya	1.23 (0.14,4.63)	0.0207 (0.0024,0.0769)	8.32 (5.63,11.09)
Lithuania	38.38 (18.23,64.76)	1.2104 (0.5803,2.0754)	8.78 (6.55,11.05)
Luxembourg	0.13 (0.02,0.45)	0.02 (0.0028,0.0709)	15.27 (12.49,18.13)
Madagascar	13.23 (2.27,42.6)	0.0917 (0.0155,0.3003)	12.78 (10.17,15.45)
Malawi	8.13 (1.66,23.36)	0.0775 (0.0155,0.2214)	10.79 (7.35,14.35)

Malaysia	15.79 (3.64,47.41)	0.0528 (0.0121,0.1588)	4.52 (1.88,7.23)
Maldives	0.09 (0.01,0.35)	0.0218 (0.0027,0.0836)	-3.95 (-6.01,-1.84)
Mali	6.05 (0.96,17.36)	0.0586 (0.0092,0.1683)	8.25 (6.16,10.38)
Malta	0.07 (0.01,0.28)	0.0187 (0.0021,0.071)	9.73 (5.91,13.7)
Marshall Islands	0.21 (0.03,0.87)	0.5109 (0.0632,2.0654)	10.73 (7.19,14.37)
Mauritania	0.5 (0.09,1.46)	0.0203 (0.0036,0.0603)	6.01 (3.66,8.41)
Mauritius	0.06 (0.01,0.19)	0.0041 (7e-04,0.0126)	3.88 (1.42,6.39)
Mexico	54.51 (10.29,161.94)	0.0412 (0.0078,0.1219)	7.87 (5.01,10.8)
Moldova	189.21 (111.34,280.9)	4.4288 (2.6226,6.5499)	14.63 (12.04,17.29)
Monaco	0.01 (0,0.02)	0.0085 (0.0011,0.0319)	8.88 (7.33,10.47)
Mongolia	67.63 (16.24,172.76)	2.0877 (0.5038,5.3421)	14.08 (11.6,16.62)
Montenegro	0.08 (0.01,0.29)	0.0108 (0.0016,0.0366)	6.71 (3.45,10.08)
Morocco	21 (5.38,55.03)	0.0625 (0.0157,0.1633)	8.65 (5.47,11.91)
Mozambique	46.16 (12.11,110.8)	0.2976 (0.0813,0.7127)	11.31 (9.02,13.66)
Myanmar	518.59 (168.81,1162.42)	1.0482 (0.3406,2.3848)	7.39 (4.89,9.95)
Namibia	4.18 (1.48,8.92)	0.208 (0.0762,0.4503)	16.16 (12.3,20.15)
Nauru	0 (0,0.02)	0.0511 (0.005,0.2377)	5.06 (2.72,7.46)
Nepal	63.39 (15,157.97)	0.2529 (0.0603,0.6185)	7.13 (5.12,9.18)
Netherlands	2.16 (0.64,5.29)	0.0106 (0.0031,0.0254)	11.82 (10.14,13.54)
New Zealand	0.66 (0.13,1.87)	0.0125 (0.0025,0.0359)	10.76 (9.23,12.32)
Nicaragua	2.99 (0.49,10.63)	0.0513 (0.0084,0.1853)	4.61 (2.81,6.44)
Niger	7.15 (1.24,20.01)	0.0611 (0.0101,0.1693)	7.77 (5.61,9.97)
Nigeria	99.01 (26.79,282.1)	0.0767 (0.0209,0.2207)	10.69 (7.47,14.01)
Niue	0 (0,0)	0.0106 (0.0011,0.0444)	5.38 (3.61,7.18)
North Korea	356.33 (103.26,817.34)	1.1868 (0.3466,2.7362)	11 (8.22,13.85)
North Macedonia	0.92 (0.24,2.4)	0.0347 (0.0091,0.0886)	6.72 (4.3,9.19)
Northern Mariana Islands	0.03 (0,0.14)	0.0688 (0.0094,0.2897)	4.55 (1.11,8.1)
Norway	1.04 (0.26,2.95)	0.0141 (0.0037,0.0398)	6.72 (4.96,8.51)
Oman	0.22 (0.04,0.68)	0.0072 (0.0013,0.022)	1.81 (0.43,3.22)
Pakistan	638.73 (186.79,1572.49)	0.3988 (0.1163,0.9771)	13.02 (9.56,16.59)
Palau	0 (0,0.01)	0.0138 (0.0013,0.0605)	6.27 (4.54,8.03)
Palestine	0.28 (0.04,1.03)	0.0083 (0.001,0.0301)	7.08 (4.63,9.58)
Panama	2.38 (0.41,7.29)	0.0543 (0.0092,0.1658)	5.61 (3.05,8.22)
Papua New Guinea	55.26 (16.01,126.84)	0.9017 (0.261,2.108)	19.96 (17.45,22.53)
Paraguay	5.33 (0.71,17.02)	0.0798 (0.0107,0.2552)	7.77 (5.39,10.21)
Peru	164.22 (68.02,353.86)	0.4492 (0.1865,0.9687)	6.53 (4.17,8.95)
Philippines	842.78 (226.94,2264.66)	0.8407 (0.2282,2.2446)	13.54 (10.82,16.33)
Poland	9.88 (2.66,26.47)	0.019 (0.0051,0.0498)	2.26 (0.39,4.18)
Portugal	3.3 (0.61,10.52)	0.0215 (0.0039,0.0676)	2.54 (1.03,4.07)
Puerto Rico	0.09 (0.02,0.28)	0.0019 (3e-04,0.0061)	3.36 (0.67,6.12)
Qatar	0.49 (0.1,1.55)	0.0255 (0.0047,0.0815)	7.34 (5.56,9.14)
Romania	54.03 (16.27,120.85)	0.2399 (0.0721,0.5414)	7.47 (5.78,9.19)
Russia	5941.64 (3172.05,9373.24)	3.6222 (1.8733,5.623)	10.29 (7.12,13.56)
Rwanda	5.75 (1.87,13.26)	0.0759 (0.024,0.1776)	8.79 (4.8,12.93)

Saint Kitts and Nevis	0 (0,0.01)	0.0035 (5e-04,0.0138)	0.69 (-0.81,2.21)
Saint Lucia	0.01 (0,0.03)	0.0038 (6e-04,0.0141)	-4.16 (-5.8,-2.49)
Saint Vincent and the Grenadines	0.01 (0,0.02)	0.0051 (7e-04,0.0174)	-2.76 (-3.93,-1.58)
Samoa	0.01 (0,0.02)	0.0039 (5e-04,0.0148)	1.55 (-1.14,4.3)
San Marino	0 (0,0.01)	0.0033 (5e-04,0.0125)	8.9 (7.34,10.49)
São Tomé and Príncipe	0.03 (0,0.1)	0.0248 (0.0035,0.078)	9.12 (6.64,11.66)
Saudi Arabia	13.09 (2.59,45.04)	0.0475 (0.0094,0.1646)	7.42 (4.19,10.76)
Senegal	3.22 (0.82,8.28)	0.0354 (0.009,0.0918)	9.57 (6.72,12.5)
Serbia	2.04 (0.49,5.37)	0.0168 (0.004,0.0445)	5.95 (4.01,7.94)
Seychelles	0.01 (0,0.03)	0.0058 (6e-04,0.0225)	0.62 (-1.87,3.16)
Sierra Leone	2.73 (0.5,8.47)	0.0511 (0.0095,0.1537)	8.83 (6.56,11.15)
Singapore	2.88 (0.91,7.14)	0.0384 (0.012,0.0948)	9.7 (8.22,11.2)
Slovakia	0.49 (0.08,1.47)	0.0067 (0.0011,0.0202)	3.92 (1.61,6.27)
Slovenia	0.02 (0,0.08)	8e-04 (1e-04,0.0029)	-5.8 (-8.25,-3.29)
Solomon Islands	0.14 (0.01,0.6)	0.0315 (0.0025,0.1318)	6.33 (4.42,8.27)
Somalia	54.2 (13.06,160.43)	0.6796 (0.1641,2.0173)	16.86 (12.73,21.14)
South Africa	58.92 (16.7,149.22)	0.1055 (0.0301,0.2679)	10.68 (7.45,14.01)
South Korea	49.94 (7.21,193.82)	0.0635 (0.0093,0.2482)	1.38 (-1.18,4)
South Sudan	11.12 (2.17,33.13)	0.2024 (0.0401,0.5934)	13.11 (10.67,15.6)
Spain	5.61 (0.83,22.39)	0.0096 (0.0014,0.0376)	4.03 (2.25,5.85)
Sri Lanka	3.59 (0.41,14.37)	0.0145 (0.0016,0.0581)	3.11 (1.19,5.06)
Sudan	11.82 (1.59,45.89)	0.0388 (0.0053,0.152)	5.52 (3.03,8.07)
Suriname	0.03 (0,0.1)	0.0045 (7e-04,0.0168)	1.47 (0.39,2.55)
Sweden	3.05 (0.93,7.58)	0.0265 (0.0082,0.0651)	13.56 (11.38,15.78)
Switzerland	2.31 (0.75,5.85)	0.0244 (0.0079,0.062)	13.15 (11.31,15.02)
Syria	1.23 (0.16,4.46)	0.0096 (0.0012,0.0351)	4.51 (1.94,7.14)
Taiwan (province of China)	19.11 (2.81,69.33)	0.0597 (0.0088,0.2138)	0.03 (-1.16,1.23)
Tajikistan	167.67 (65.18,320.68)	1.8047 (0.6995,3.4738)	15.42 (11.4,19.57)
Tanzania	19.4 (3.38,68.64)	0.057 (0.0098,0.2084)	10.68 (8,13.42)
Thailand	226.26 (52.83,592.47)	0.2585 (0.0612,0.671)	7.77 (6.47,9.09)
The Bahamas	0.05 (0.01,0.18)	0.0118 (0.0016,0.0441)	11.14 (9.17,13.14)
The Gambia	0.64 (0.1,1.89)	0.0487 (0.0079,0.1432)	8.12 (6.03,10.26)
Timor-Leste	1.92 (0.23,8.14)	0.1968 (0.0237,0.8234)	1.13 (-1.13,3.45)
Togo	2.65 (0.39,8.65)	0.0563 (0.0083,0.1791)	9.25 (7.01,11.54)
Tokelau	0 (0,0)	0.0107 (0.0011,0.0397)	3.84 (2.03,5.68)
Tonga	0.01 (0,0.05)	0.0117 (0.0012,0.0588)	6.12 (4.26,8.01)
Trinidad and Tobago	0.05 (0.01,0.19)	0.0035 (4e-04,0.0119)	1.72 (0.33,3.13)
Tunisia	1.42 (0.4,3.99)	0.0116 (0.0033,0.0326)	5.76 (3.08,8.51)
Türkiye	15.72 (5.68,32.8)	0.0179 (0.0065,0.0373)	6.69 (3.73,9.73)
Turkmenistan	87.42 (27.25,187.48)	1.7009 (0.5323,3.6315)	15.12 (11.31,19.05)
Tuvalu	0.01 (0,0.04)	0.0803 (0.0079,0.355)	4.8 (3.09,6.54)
Uganda	22.9 (5.73,60.65)	0.0991 (0.0249,0.2608)	12.55 (8.82,16.4)
UK	8.1 (2.1,21.56)	0.0116 (0.003,0.0313)	8.88 (7.43,10.34)
Ukraine	1938.28 (887.09,3382.24)	3.8265 (1.7035,6.5804)	13.87 (11.63,16.16)

United Arab Emirates	0.33 (0.04,1.26)	0.0095 (0.0012,0.0376)	7.53 (5.1,10.01)
Uruguay	0.33 (0.06,1.09)	0.0085 (0.0016,0.0286)	9.87 (8.15,11.63)
USA	15.98 (5.3,43.68)	0.0039 (0.0013,0.0108)	4.87 (4.36,5.39)
Uzbekistan	837.34 (270.67,1671.71)	2.5779 (0.837,5.1338)	18.57 (14.27,23.04)
Vanuatu	0.04 (0,0.17)	0.021 (0.0022,0.0854)	11.5 (9.82,13.21)
Venezuela	10.72 (1.73,36.93)	0.0386 (0.0063,0.1345)	6.46 (4.38,8.59)
Viet Nam	580.79 (146.64,1478.52)	0.6077 (0.1541,1.5487)	8.93 (6.87,11.03)
Virgin Islands	0 (0,0.01)	0.0017 (2e-04,0.0068)	0.85 (-0.57,2.3)
Yemen	5.96 (1.12,21.12)	0.0312 (0.0057,0.1108)	6.19 (3.34,9.12)
Zambia	10.03 (1.65,30.43)	0.0774 (0.0131,0.2355)	6.24 (3.73,8.82)
Zimbabwe	22.68 (3.59,75.92)	0.2318 (0.0353,0.7953)	17.74 (14.46,21.11)

Abbreviations: MDR-TB=multidrug-resistant tuberculosis. XDR-TB=extensively drug-resistant tuberculosis. ASR=age-standardized rate. EAPC=estimated annual percentage change.

Table S9 The death of cases and ASR of XDR-TB in 2021, and the trends from baseline to 2021 by countries.

Location	Deaths_Number_2021	Deaths_ASR_2021	EAPC
Afghanistan	54.34 (6.42,192.12)	0.3936 (0.0473,1.4913)	10.84 (6.9,14.92)
Albania	0.23 (0.03,0.81)	0.0059 (7e-04,0.0207)	3.72 (1.51,5.99)
Algeria	4.9 (0.47,18.6)	0.0162 (0.0016,0.0625)	5.01 (3.43,6.61)
American Samoa	0 (0,0.01)	0.0029 (2e-04,0.0121)	5.02 (3.3,6.78)
Andorra	0 (0,0)	1e-04 (0,4e-04)	4.28 (2.74,5.85)
Angola	9.29 (1.07,30.66)	0.0597 (0.0069,0.1989)	8.31 (5.95,10.72)
Antigua and Barbuda	0 (0,0)	6e-04 (1e-04,0.0026)	-2.11 (-3.73,-0.47)
Argentina	5.54 (0.67,22.67)	0.0103 (0.0013,0.0423)	4.4 (1.83,7.03)
Armenia	3.8 (0.9,8.18)	0.1006 (0.0237,0.2165)	10.48 (5.67,15.5)
Australia	1.31 (0.32,3.41)	0.0027 (7e-04,0.0071)	11.99 (10.95,13.03)
Austria	0.86 (0.2,2.14)	0.0044 (0.001,0.0108)	10.36 (8.04,12.72)
Azerbaijan	51.74 (16.09,107.05)	0.4713 (0.1497,0.963)	14.29 (9.71,19.06)
Bahrain	0.05 (0.01,0.19)	0.0085 (0.0011,0.031)	6.99 (3.65,10.44)
Bangladesh	129.55 (27.31,341.08)	0.0978 (0.0204,0.2555)	8.52 (4.85,12.31)
Barbados	0 (0,0.01)	4e-04 (0,0.0019)	-5.56 (-7.53,-3.55)
Belarus	45.8 (25.07,71.06)	0.3373 (0.1854,0.5243)	9.26 (5.46,13.2)

Belgium	0.59 (0.11,1.76)	0.0023 (4e-04,0.0068)	3.31 (1.4,5.25)
Belize	0.02 (0,0.08)	0.0055 (5e-04,0.0234)	-0.08 (-2.09,1.97)
Benin	1.12 (0.17,3.88)	0.0181 (0.0027,0.0624)	10.13 (7.05,13.31)
Bermuda	0 (0,0)	1e-04 (0,3e-04)	-4.37 (-6.3,-2.39)
Bhutan	0.47 (0.06,1.61)	0.0756 (0.0095,0.2561)	6.94 (4.68,9.26)
Bolivia	23.56 (3.24,78.26)	0.249 (0.0344,0.8299)	4.74 (2.92,6.6)
Bosnia and Herzegovina	0.44 (0.08,1.52)	0.0076 (0.0013,0.0258)	4.08 (2.38,5.82)
Botswana	1.04 (0.17,3.54)	0.0584 (0.0093,0.1967)	15.27 (12.14,18.49)
Brazil	55.29 (8.18,175.01)	0.0223 (0.0033,0.0704)	10.51 (6.87,14.27)
Brunei	0.02 (0,0.09)	0.0093 (9e-04,0.0338)	3.96 (2.15,5.81)
Bulgaria	1.55 (0.28,4.9)	0.0128 (0.0023,0.0404)	6.54 (3.59,9.58)
Burkina Faso	4.85 (0.75,15.51)	0.0437 (0.0067,0.1366)	10.71 (8.43,13.04)
Burundi	6.83 (0.85,22.51)	0.1201 (0.0151,0.3893)	10.33 (7.49,13.25)
Cabo Verde	0.06 (0.01,0.2)	0.0128 (0.0015,0.0445)	7.58 (5.29,9.93)
Cambodia	19.39 (2.17,71.09)	0.159 (0.0184,0.5746)	2.22 (-1.3,5.87)
Cameroon	3.45 (0.46,12.41)	0.0239 (0.0031,0.0869)	7.71 (5.24,10.24)
Canada	0.81 (0.18,2.24)	0.0011 (2e-04,0.0031)	3.77 (2.53,5.02)
Central African Republic	3.35 (0.38,12.49)	0.1145 (0.0131,0.4211)	8.54 (6.54,10.58)
Chad	4.85 (0.67,14.95)	0.0631 (0.0085,0.1952)	9.45 (6.88,12.09)
Chile	2.63 (0.53,7.66)	0.0106 (0.0021,0.0309)	6.52 (4.36,8.73)
China	561.18 (87.56,1714.74)	0.0287 (0.0045,0.0874)	-2.89 (-4.95,-0.78)
Colombia	14.2 (2.3,43.1)	0.0261 (0.0042,0.0791)	8.83 (6.56,11.14)
Comoros	0.64 (0.08,1.96)	0.1349 (0.0159,0.4054)	13.08 (10.37,15.86)
Congo (Brazzaville)	1.16 (0.12,4.71)	0.0374 (0.0039,0.1519)	7.21 (5.05,9.42)
Cook Islands	0 (0,0)	0.0012 (1e-04,0.0053)	3.12 (1.32,4.95)
Costa Rica	0.37 (0.05,1.34)	0.0067 (8e-04,0.0245)	4.93 (2.03,7.91)
Côte d'Ivoire	4.62 (0.63,14.96)	0.0356 (0.005,0.1192)	3.9 (2.16,5.66)
Croatia	0.16 (0.03,0.51)	0.0019 (3e-04,0.0061)	-0.24 (-2.68,2.26)
Cuba	0.25 (0.04,0.77)	0.0014 (2e-04,0.0043)	3.33 (2.32,4.36)
Cyprus	0.05 (0.01,0.17)	0.0031 (5e-04,0.0101)	3.32 (-0.03,6.78)
Czechia	0.5 (0.11,1.41)	0.0025 (6e-04,0.0071)	4.3 (2.08,6.55)
Denmark	0.16 (0.03,0.52)	0.0013 (2e-04,0.0042)	8.59 (6.75,10.47)
Djibouti	0.82 (0.2,2.08)	0.1279 (0.0324,0.3179)	16.53 (12.08,21.16)
Dominica	0 (0,0.02)	0.0061 (7e-04,0.0231)	-0.34 (-1.67,1)
Dominican Republic	1.16 (0.13,5.2)	0.011 (0.0012,0.0495)	2.03 (0.11,3.98)
DR Congo	34.22 (4.68,133.54)	0.0769 (0.0102,0.3)	10.15 (7.92,12.42)
Ecuador	7.46 (1.03,25.56)	0.0447 (0.0062,0.1529)	2.41 (-0.45,5.35)
Egypt	6.17 (1.05,20.96)	0.0103 (0.0017,0.0351)	8.54 (5.17,12.01)
El Salvador	1.35 (0.16,4.59)	0.0213 (0.0025,0.0725)	2.57 (0.28,4.92)
Equatorial Guinea	0.15 (0.02,0.58)	0.0229 (0.003,0.0867)	3.61 (2.15,5.09)
Eritrea	5.34 (0.73,18.55)	0.1676 (0.0224,0.5691)	12.27 (9.35,15.26)
Estonia	2.32 (0.91,4.21)	0.1131 (0.0443,0.2039)	2.5 (-0.34,5.42)
Eswatini	1.71 (0.33,4.54)	0.2382 (0.0453,0.6339)	16.3 (11.57,21.23)
Ethiopia	29.03 (3.73,88.17)	0.0601 (0.0075,0.1861)	7.34 (4.43,10.34)

Federated States of Micronesia	0.01 (0,0.07)	0.0189 (0.0014,0.0863)	5.37 (3.51,7.26)
Fiji	0.03 (0,0.15)	0.0044 (4e-04,0.0191)	10.29 (8.47,12.15)
Finland	0.78 (0.15,2.28)	0.0056 (0.0011,0.0164)	12.21 (10.11,14.34)
France	7.21 (1.59,21.05)	0.0041 (9e-04,0.0117)	4.54 (2.32,6.81)
Gabon	0.37 (0.05,1.35)	0.033 (0.0042,0.123)	9.49 (7.3,11.73)
Georgia	13.76 (5.55,25.13)	0.2771 (0.111,0.5059)	11.13 (7.47,14.91)
Germany	8.97 (2.29,21.97)	0.0043 (0.0011,0.0105)	7.78 (6.27,9.32)
Ghana	6.49 (0.77,25.43)	0.0387 (0.0046,0.1503)	9.43 (7.34,11.55)
Greece	1.45 (0.24,4.98)	0.0054 (9e-04,0.0186)	10.66 (7.09,14.35)
Greenland	0.03 (0,0.09)	0.0489 (0.0058,0.1767)	5.01 (3.94,6.08)
Grenada	0 (0,0)	9e-04 (1e-04,0.0041)	1.2 (-0.49,2.92)
Guam	0.01 (0,0.06)	0.0069 (6e-04,0.0281)	5.93 (2.58,9.38)
Guatemala	4.12 (0.6,13.71)	0.0345 (0.0051,0.1147)	1.35 (-0.73,3.47)
Guinea	2.05 (0.26,7.79)	0.0321 (0.0041,0.1204)	8.72 (6.4,11.08)
Guinea-Bissau	0.48 (0.06,1.75)	0.0605 (0.0074,0.2161)	7.65 (5.14,10.22)
Guyana	0.11 (0.01,0.47)	0.0153 (0.0015,0.0678)	4.7 (2.49,6.96)
Haiti	2.16 (0.18,10.29)	0.0242 (0.0019,0.1153)	3.52 (1.97,5.1)
Honduras	5.7 (0.73,18.16)	0.0871 (0.0112,0.2724)	8.48 (5.64,11.39)
Hungary	0.61 (0.09,1.93)	0.0035 (5e-04,0.0112)	0.17 (-2.86,3.3)
Iceland	0.01 (0,0.02)	0.001 (1e-04,0.0036)	3.09 (-0.98,7.33)
India	2815.29 (502.44,7178.1)	0.2317 (0.0416,0.5892)	11.87 (8.4,15.44)
Indonesia	134.32 (15.32,530.45)	0.0632 (0.0072,0.2534)	-0.26 (-3.07,2.63)
Iran	2.53 (0.53,6.62)	0.0035 (7e-04,0.009)	9.19 (5.96,12.51)
Iraq	4.36 (0.86,12.22)	0.0189 (0.0036,0.0531)	8.63 (4.79,12.61)
Ireland	0.18 (0.03,0.56)	0.0022 (3e-04,0.0069)	4.82 (2.6,7.09)
Israel	0.76 (0.17,2.02)	0.0058 (0.0013,0.0155)	1.87 (-0.03,3.81)
Italy	3.86 (0.79,10.87)	0.0024 (5e-04,0.0067)	2.86 (0.11,5.67)
Jamaica	0.07 (0.01,0.29)	0.0023 (3e-04,0.0093)	6.02 (4.45,7.61)
Japan	16.83 (1.52,66.64)	0.0029 (3e-04,0.0115)	-0.86 (-2.7,1.02)
Jordan	0.3 (0.04,1.08)	0.0043 (6e-04,0.0154)	6.12 (2.79,9.55)
Kazakhstan	78.57 (35.1,137.08)	0.4048 (0.1805,0.7024)	3.74 (-0.71,8.39)
Kenya	12.54 (2.36,36.23)	0.0518 (0.0095,0.1484)	12.75 (8.49,17.17)
Kiribati	0.1 (0.01,0.49)	0.1202 (0.0095,0.5965)	6.04 (3.91,8.22)
Kuwait	0.12 (0.02,0.38)	0.0045 (7e-04,0.0143)	7 (3.99,10.1)
Kyrgyzstan	47.71 (16.76,85.94)	0.795 (0.2796,1.4373)	14.17 (8.83,19.77)
Laos	3.39 (0.28,14.1)	0.0695 (0.0058,0.2887)	-1.85 (-4.5,0.88)
Latvia	3.34 (1.12,7.23)	0.1126 (0.0379,0.2447)	1.08 (-0.99,3.2)
Lebanon	0.16 (0.02,0.6)	0.0026 (2e-04,0.0096)	4.48 (1.78,7.25)
Lesotho	3.72 (0.79,10.9)	0.2896 (0.0615,0.8452)	19.52 (15.32,23.88)
Liberia	0.44 (0.05,1.62)	0.0183 (0.0021,0.0656)	5.52 (3.42,7.66)
Libya	0.5 (0.05,2.08)	0.0099 (0.001,0.0406)	8.96 (5.92,12.09)
Lithuania	11.24 (4.64,19.49)	0.2693 (0.1116,0.4651)	6.37 (3.58,9.23)
Luxembourg	0.02 (0,0.06)	0.0014 (2e-04,0.0051)	9.99 (7.19,12.87)
Madagascar	8.73 (1.09,32.93)	0.0679 (0.0085,0.2554)	13.19 (10.21,16.25)

Malawi	4.44 (0.65,14.17)	0.0542 (0.0082,0.1751)	10.94 (7.22,14.79)
Malaysia	7.04 (1.25,23.06)	0.0259 (0.0046,0.0849)	4.24 (0.92,7.67)
Maldives	0.03 (0,0.14)	0.0098 (8e-04,0.0407)	-5.44 (-7.74,-3.1)
Mali	3.78 (0.47,12.5)	0.0433 (0.0057,0.1445)	8.23 (6.08,10.43)
Malta	0 (0,0.01)	3e-04 (0,0.0013)	3.11 (-0.9,7.29)
Marshall Islands	0.13 (0.01,0.58)	0.3523 (0.0351,1.5349)	10.77 (7.04,14.63)
Mauritania	0.33 (0.04,1.15)	0.0149 (0.0019,0.0521)	5.92 (3.49,8.42)
Mauritius	0.02 (0,0.08)	0.0013 (2e-04,0.0046)	2.3 (-0.81,5.51)
Mexico	24.5 (3.73,80.39)	0.0189 (0.0029,0.0623)	6.66 (3.61,9.8)
Moldova	27.23 (15.2,40.71)	0.5271 (0.2948,0.7874)	9.91 (6.22,13.73)
Monaco	0 (0,0.02)	0.0036 (4e-04,0.0151)	7.95 (6.41,9.51)
Mongolia	32.19 (7.08,83.29)	1.0141 (0.2249,2.6238)	12.62 (9.84,15.46)
Montenegro	0.03 (0,0.14)	0.0039 (5e-04,0.0158)	5.87 (2.32,9.54)
Morocco	11 (1.72,32.81)	0.0346 (0.0054,0.1022)	8.54 (4.98,12.22)
Mozambique	28.07 (6.13,73.56)	0.2166 (0.0463,0.5556)	11.24 (8.82,13.71)
Myanmar	323.25 (73.06,860.95)	0.6829 (0.1548,1.8535)	7.02 (4.21,9.9)
Namibia	1.72 (0.47,4.61)	0.1099 (0.0299,0.289)	15.38 (11.24,19.68)
Nauru	0 (0,0.01)	0.0332 (0.0022,0.1724)	4.83 (2.29,7.43)
Nepal	37.85 (6.93,112.36)	0.1642 (0.0301,0.4792)	6.73 (4.72,8.79)
Netherlands	0.83 (0.18,2.19)	0.0022 (5e-04,0.0059)	8.82 (7.04,10.63)
New Zealand	0.05 (0.01,0.16)	6e-04 (1e-04,0.0019)	3.36 (0.65,6.14)
Nicaragua	1.5 (0.19,5.41)	0.0285 (0.0036,0.1031)	3.84 (1.82,5.91)
Niger	4.74 (0.55,15.56)	0.0458 (0.0052,0.1537)	7.81 (5.58,10.08)
Nigeria	53.95 (10.47,151.31)	0.0501 (0.0097,0.1383)	10.81 (7.39,14.34)
Niue	0 (0,0)	0.0062 (5e-04,0.0286)	4.91 (2.89,6.97)
North Korea	197.02 (43.53,582.92)	0.6171 (0.1356,1.8151)	11.5 (7.81,15.32)
North Macedonia	0.35 (0.06,1.12)	0.0133 (0.0022,0.0411)	5.18 (2.48,7.96)
Northern Mariana Islands	0.01 (0,0.06)	0.0304 (0.0032,0.1258)	3.39 (0,6.89)
Norway	0.49 (0.1,1.41)	0.0042 (8e-04,0.0121)	5.19 (3.25,7.16)
Oman	0.06 (0.01,0.18)	0.003 (4e-04,0.0093)	1.35 (-0.38,3.11)
Pakistan	404.54 (88.12,1217.03)	0.2801 (0.0601,0.8437)	13.36 (9.47,17.4)
Palau	0 (0,0.01)	0.0083 (7e-04,0.0371)	5.97 (4,7.99)
Palestine	0.09 (0.01,0.41)	0.004 (4e-04,0.0179)	6.49 (3.69,9.37)
Panama	1.15 (0.15,3.84)	0.026 (0.0034,0.0869)	4.41 (1.7,7.19)
Papua New Guinea	35.97 (7.77,98.58)	0.6528 (0.1403,1.7848)	19.84 (17.16,22.57)
Paraguay	2.63 (0.32,8.67)	0.0427 (0.0052,0.1404)	7.81 (5.07,10.62)
Peru	61.8 (19.75,145.28)	0.1766 (0.0563,0.4137)	4.65 (2.17,7.19)
Philippines	272.46 (61.46,706.02)	0.3202 (0.0719,0.8303)	12.1 (8.61,15.7)
Poland	3.2 (0.72,8.78)	0.005 (0.0011,0.0137)	0.3 (-1.79,2.43)
Portugal	1.69 (0.22,5.43)	0.0064 (8e-04,0.0206)	1.24 (-0.49,3)
Puerto Rico	0.05 (0.01,0.18)	9e-04 (1e-04,0.0029)	2.36 (-0.61,5.41)
Qatar	0.08 (0.01,0.28)	0.0091 (0.0013,0.0341)	5.16 (3,7.38)
Romania	20.48 (5.15,53.47)	0.0699 (0.0175,0.1834)	5.1 (2.98,7.27)
Russia	805.36 (413.79,1333.49)	0.4088 (0.2093,0.6768)	7.29 (3.53,11.2)

Rwanda	3.55 (0.72,9.17)	0.0542 (0.0111,0.1408)	8.56 (4.41,12.87)
Saint Kitts and Nevis	0 (0,0.01)	0.0019 (2e-04,0.0087)	0.04 (-1.55,1.67)
Saint Lucia	0.01 (0,0.02)	0.0023 (3e-04,0.0089)	-4.56 (-6.28,-2.82)
Saint Vincent and the Grenadines	0 (0,0.01)	0.0023 (3e-04,0.0089)	-3.56 (-4.9,-2.2)
Samoa	0 (0,0.02)	0.0026 (2e-04,0.0118)	1.56 (-1.3,4.51)
San Marino	0 (0,0)	9e-04 (1e-04,0.0032)	7.41 (5.93,8.91)
São Tomé and Príncipe	0.02 (0,0.07)	0.0168 (0.0018,0.0583)	8.9 (6.26,11.6)
Saudi Arabia	4.74 (0.7,17.28)	0.0242 (0.0037,0.0843)	6.82 (3.2,10.58)
Senegal	2.07 (0.41,5.94)	0.0258 (0.005,0.0745)	10 (6.9,13.19)
Serbia	0.94 (0.17,2.68)	0.0061 (0.0011,0.0175)	3.83 (1.52,6.2)
Seychelles	0 (0,0.01)	0.0027 (2e-04,0.0119)	-0.08 (-3.15,3.08)
Sierra Leone	1.63 (0.23,5.43)	0.0356 (0.0048,0.1176)	9.29 (6.79,11.84)
Singapore	0.36 (0.09,1.09)	0.0044 (0.0011,0.0134)	4.52 (2.43,6.65)
Slovakia	0.21 (0.03,0.8)	0.0024 (3e-04,0.0093)	2.09 (-0.33,4.56)
Slovenia	0.01 (0,0.02)	2e-04 (0,6e-04)	-8.06 (-10.99,-5.03)
Solomon Islands	0.09 (0.01,0.4)	0.0223 (0.0016,0.0961)	6.29 (4.25,8.36)
Somalia	38.33 (7.22,117.44)	0.5241 (0.1029,1.6128)	17.38 (12.89,22.05)
South Africa	19.09 (4.11,54.1)	0.0383 (0.0081,0.1093)	9.22 (5.55,13.01)
South Korea	17.2 (2.15,65.46)	0.0191 (0.0024,0.0729)	-1.77 (-4.44,0.97)
South Sudan	6.97 (1.03,22.62)	0.1428 (0.022,0.4873)	13.34 (10.62,16.12)
Spain	2.18 (0.26,8.42)	0.0019 (2e-04,0.0076)	1.48 (-0.33,3.32)
Sri Lanka	1.36 (0.14,5.78)	0.0053 (5e-04,0.0226)	1.3 (-1.2,3.87)
Sudan	4.39 (0.49,17.21)	0.0196 (0.0023,0.0772)	4.88 (2.05,7.8)
Suriname	0.01 (0,0.06)	0.0022 (2e-04,0.0093)	0.88 (-0.49,2.27)
Sweden	0.87 (0.21,2.41)	0.0032 (8e-04,0.0089)	7.56 (5.14,10.04)
Switzerland	0.59 (0.15,1.5)	0.0028 (7e-04,0.0071)	8.03 (5.97,10.13)
Syria	0.41 (0.04,1.59)	0.0036 (3e-04,0.0137)	3.22 (0.23,6.3)
Taiwan (province of China)	5.52 (0.7,22.86)	0.0131 (0.0016,0.0541)	-3.4 (-5.26,-1.5)
Tajikistan	74.15 (25.8,148.94)	0.8798 (0.3017,1.7877)	14.84 (10.34,19.53)
Tanzania	11.8 (1.74,46.16)	0.0404 (0.0059,0.1555)	11.24 (8.17,14.39)
Thailand	88.29 (15.83,270.03)	0.0858 (0.0153,0.2649)	5.08 (3.45,6.74)
The Bahamas	0.03 (0,0.09)	0.0066 (7e-04,0.0233)	10.52 (8.33,12.76)
The Gambia	0.37 (0.05,1.24)	0.0341 (0.0047,0.1182)	8.37 (6.05,10.74)
Timor-Leste	1.22 (0.11,5.37)	0.1376 (0.0123,0.6014)	1.19 (-1.3,3.73)
Togo	1.61 (0.19,6.02)	0.0398 (0.0048,0.1525)	9.33 (6.91,11.79)
Tokelau	0 (0,0)	0.0064 (5e-04,0.0285)	3.42 (1.44,5.44)
Tonga	0.01 (0,0.03)	0.007 (6e-04,0.0325)	6.12 (3.99,8.3)
Trinidad and Tobago	0.03 (0,0.11)	0.0015 (2e-04,0.006)	0.09 (-1.57,1.76)
Tunisia	0.54 (0.11,1.75)	0.0044 (9e-04,0.0143)	6.03 (2.84,9.32)
Türkiye	5.9 (1.57,15.45)	0.0068 (0.0018,0.0178)	5.14 (1.85,8.53)
Turkmenistan	39.48 (12.66,82.23)	0.7878 (0.2525,1.6387)	14.57 (9.8,19.55)
Tuvalu	0.01 (0,0.03)	0.0517 (0.0038,0.2538)	4.48 (2.63,6.37)
Uganda	12.17 (2.33,41.83)	0.0687 (0.0126,0.2341)	12.53 (8.53,16.68)
UK	2.07 (0.4,6.42)	0.0016 (3e-04,0.0049)	6 (4.62,7.39)

Ukraine	400.25 (170.42,700.23)	0.6423 (0.2749,1.1201)	10.65 (7.38,14.03)
United Arab Emirates	0.11 (0.01,0.47)	0.0058 (6e-04,0.0245)	7.85 (5.12,10.65)
Uruguay	0.1 (0.01,0.33)	0.0019 (3e-04,0.0066)	6.91 (4.9,8.96)
USA	6.35 (1.59,17.97)	0.0012 (3e-04,0.0033)	2.69 (2.22,3.18)
Uzbekistan	190.84 (66.52,340.57)	0.5941 (0.2074,1.0571)	15.19 (9.93,20.71)
Vanuatu	0.03 (0,0.12)	0.0152 (0.0013,0.0694)	11.41 (9.61,13.24)
Venezuela	5.89 (0.75,22.27)	0.0204 (0.0026,0.0773)	5.53 (3.17,7.94)
Viet Nam	285.81 (50.86,819.27)	0.3185 (0.0566,0.9024)	7.79 (5.56,10.06)
Virgin Islands	0 (0,0.01)	9e-04 (1e-04,0.0036)	0.45 (-1.23,2.15)
Yemen	3.25 (0.46,11.84)	0.021 (0.0031,0.0756)	6.15 (3.06,9.34)
Zambia	3.1 (0.39,10.45)	0.0411 (0.005,0.135)	4.91 (2.22,7.67)
Zimbabwe	14.04 (1.95,46.15)	0.1647 (0.0229,0.5466)	18.59 (15.06,22.22)

Abbreviations: MDR-TB=multidrug-resistant tuberculosis. XDR-TB=extensively drug-resistant tuberculosis, ASR=age-standardized rate. EAPC=estimated annual percentage change.

Table S10 The incidence of cases and ASR of HIV/AIDS-MDR-TB in 2021, and the trends from baseline to 2021 by countries.

Location	Incidence_Number_2021	Incidence_ASR_2021	EAPC
Afghanistan	1.81 (0.25,5.87)	0.0079 (0.0011,0.0255)	5.49 (2.05,9.04)
Albania	0.05 (0.01,0.16)	0.0018 (3e-04,0.0059)	-1.24 (-3.17,0.72)
Algeria	4.89 (0.57,19.5)	0.0105 (0.0012,0.0419)	0.07 (-0.34,0.49)
American Samoa	0 (0,0)	0.0012 (1e-04,0.0051)	-0.13 (-1.93,1.69)
Andorra	0 (0,0)	3e-04 (0,0.001)	-6.77 (-7.87,-5.65)
Angola	398.89 (59.87,1191.98)	1.6183 (0.243,4.8347)	7.45 (4.93,10.03)
Antigua and Barbuda	0.01 (0,0.06)	0.0127 (0.0018,0.0554)	-7.35 (-8.71,-5.97)
Argentina	37.83 (6.06,135.2)	0.0752 (0.012,0.2686)	-0.75 (-2.87,1.41)
Armenia	5.79 (1.33,13.51)	0.163 (0.0374,0.3804)	11.53 (7.82,15.36)
Australia	1.38 (0.43,3.48)	0.0043 (0.0013,0.0108)	-0.14 (-1.13,0.86)
Austria	0.72 (0.22,1.68)	0.0065 (0.002,0.0153)	-0.26 (-1.2,0.68)
Azerbaijan	19.54 (5.65,40.85)	0.1571 (0.0454,0.3286)	12.74 (8.89,16.73)
Bahrain	0.2 (0.03,0.83)	0.0109 (0.0016,0.0441)	2.56 (0.05,5.13)
Bangladesh	135.68 (37.86,362.92)	0.0804 (0.0224,0.215)	15.99 (12.06,20.07)
Barbados	0.01 (0,0.04)	0.0024 (2e-04,0.0102)	-12.73 (-14.66,-10.75)

Belarus	45.06 (27.15,70.38)	0.4229 (0.2549,0.6606)	7.59 (5,10.25)
Belgium	0.88 (0.21,2.39)	0.006 (0.0014,0.0164)	-4.55 (-5.3,-3.8)
Belize	0.13 (0.01,0.51)	0.0293 (0.0034,0.118)	-6.39 (-7.87,-4.88)
Benin	41.06 (7.69,118.58)	0.454 (0.085,1.3115)	5.7 (1.65,9.92)
Bermuda	0.01 (0,0.04)	0.01 (7e-04,0.0483)	-7.93 (-9.69,-6.14)
Bhutan	0.19 (0.03,0.59)	0.0234 (0.0039,0.0724)	2.92 (0.3,5.61)
Bolivia	9.78 (1.66,31.35)	0.0841 (0.0143,0.2698)	4.16 (0.5,7.96)
Bosnia and Herzegovina	0.05 (0.01,0.15)	0.0012 (3e-04,0.0036)	-0.61 (-1.35,0.14)
Botswana	389.38 (79.97,1157.57)	16.7003 (3.4162,49.5918)	8.57 (5.73,11.47)
Brazil	535.63 (105.11,1531.57)	0.216 (0.0424,0.6181)	7.01 (4.11,9.98)
Brunei	0.01 (0,0.04)	0.0018 (2e-04,0.007)	-1.25 (-2.59,0.11)
Bulgaria	0.08 (0.02,0.23)	0.0012 (3e-04,0.0033)	2.96 (0.57,5.41)
Burkina Faso	136.94 (24.01,375.59)	0.9069 (0.1592,2.4871)	-3.08 (-4.8,-1.34)
Burundi	156.55 (26.07,488.2)	1.7755 (0.2948,5.5302)	-0.1 (-3.3,3.2)
Cabo Verde	1.18 (0.19,3.42)	0.2065 (0.0337,0.597)	-1.6 (-3.77,0.62)
Cambodia	19.38 (3.02,66.38)	0.1219 (0.019,0.4178)	3.25 (-3.03,9.93)
Cameroon	350.78 (58.53,1017.05)	1.4625 (0.2442,4.2372)	2.47 (-0.38,5.41)
Canada	2.49 (0.74,6.42)	0.0054 (0.0016,0.0138)	-5.64 (-6.13,-5.14)
Central African Republic	142.82 (22.87,470.14)	3.3117 (0.5312,10.9042)	-0.1 (-2.27,2.12)
Chad	161.69 (27.11,473.96)	1.5153 (0.2538,4.4428)	1.72 (-0.41,3.9)
Chile	7.35 (2.04,18.3)	0.0341 (0.0095,0.0849)	1.73 (0.54,2.93)
China	1053.15 (165.26,3231.26)	0.0612 (0.0096,0.1864)	-2.16 (-3.74,-0.54)
Colombia	64.09 (12.37,189.11)	0.1191 (0.023,0.3515)	5.63 (3.82,7.46)
Comoros	2.45 (0.34,7.9)	0.3463 (0.0479,1.1189)	10.27 (7.83,12.77)
Congo (Brazzaville)	73.33 (10.76,245.47)	1.4866 (0.2182,4.9792)	-0.75 (-2.8,1.34)
Cook Islands	0 (0,0)	0.0015 (2e-04,0.0056)	2.52 (0.96,4.11)
Costa Rica	1.01 (0.15,3.36)	0.0191 (0.0029,0.0636)	0.65 (-2.02,3.39)
Côte d'Ivoire	268.21 (45.6,785.06)	1.2701 (0.216,3.7173)	-5.81 (-7.51,-4.07)
Croatia	0.02 (0,0.05)	4e-04 (1e-04,0.0011)	-5.73 (-7.27,-4.17)
Cuba	1.13 (0.21,3.4)	0.0082 (0.0015,0.0247)	-1.21 (-2.19,-0.22)
Cyprus	0.05 (0.01,0.14)	0.0029 (5e-04,0.0088)	0.1 (-2.68,2.96)
Czechia	0.27 (0.08,0.66)	0.0024 (7e-04,0.0061)	-1.65 (-2.49,-0.81)
Denmark	0.16 (0.04,0.45)	0.0021 (5e-04,0.0058)	-1.46 (-2.35,-0.56)
Djibouti	28.09 (9.96,67.94)	2.267 (0.803,5.4837)	17.3 (11.17,23.76)
Dominica	0.01 (0,0.05)	0.0203 (0.0026,0.0722)	-8.71 (-10.13,-7.26)
Dominican Republic	3.82 (0.51,14.8)	0.034 (0.0045,0.1318)	-6.77 (-8.92,-4.58)
DR Congo	494.22 (83.76,1540.37)	0.7329 (0.1242,2.2855)	-0.51 (-2.56,1.59)
Ecuador	78.85 (13.88,275.9)	0.4263 (0.075,1.4916)	3.5 (0.39,6.71)
Egypt	5.42 (1.23,15.8)	0.0053 (0.0012,0.0154)	4.18 (1.84,6.57)
El Salvador	5.05 (0.73,16.19)	0.0799 (0.0115,0.2561)	-0.85 (-3.6,1.98)
Equatorial Guinea	23.22 (3.84,73.55)	1.8967 (0.3136,5.9872)	3.44 (1.35,5.59)
Eritrea	74.82 (14.06,222.46)	1.3862 (0.2604,4.1201)	3.89 (0.73,7.15)
Estonia	5.77 (2.48,10.96)	0.4284 (0.1843,0.8132)	-0.27 (-1.67,1.15)
Eswatini	576.8 (136.42,1484.27)	57.0104 (13.6275,148.7341)	12.96 (8.33,17.78)

Ethiopia	877.3 (136.14,3081.32)	1.1386 (0.1767,3.9356)	1.83 (-1.39,5.16)
Federated States of Micronesia	0.01 (0,0.05)	0.0109 (0.001,0.0519)	4.03 (2.48,5.61)
Fiji	0.01 (0,0.06)	0.0015 (2e-04,0.0067)	3.3 (2.28,4.33)
Finland	0.27 (0.07,0.72)	0.0041 (0.001,0.011)	3.64 (3.01,4.27)
France	7.93 (2.23,21.43)	0.0098 (0.0028,0.0266)	-6.45 (-7.17,-5.73)
Gabon	36.96 (6.41,118.99)	2.3484 (0.4074,7.5575)	3.96 (1.63,6.33)
Georgia	13.66 (6.22,23.69)	0.3144 (0.1433,0.5453)	12.6 (9.47,15.82)
Germany	9.61 (3,21.92)	0.0087 (0.0027,0.0199)	-1.86 (-2.64,-1.08)
Ghana	414.48 (65.58,1316.47)	1.43 (0.2263,4.542)	1.94 (-0.07,4)
Greece	0.93 (0.17,2.81)	0.0083 (0.0015,0.0251)	2.39 (1.07,3.73)
Greenland	0.1 (0.01,0.37)	0.1511 (0.0212,0.5331)	-1.78 (-2.32,-1.23)
Grenada	0.01 (0,0.03)	0.0065 (8e-04,0.0281)	-5.39 (-6.62,-4.13)
Guam	0.01 (0,0.04)	0.0054 (6e-04,0.0223)	1.64 (-0.91,4.26)
Guatemala	16.42 (2.75,47.57)	0.1117 (0.0187,0.3238)	-1.11 (-3.72,1.58)
Guinea	103.69 (18.81,331.34)	1.1065 (0.2008,3.5333)	3.14 (0.7,5.62)
Guinea-Bissau	47.21 (6.99,159.6)	2.9856 (0.4403,10.0638)	3.72 (1.69,5.79)
Guyana	0.59 (0.07,2.5)	0.0775 (0.0093,0.3286)	-2.51 (-4.38,-0.61)
Haiti	8.38 (1.14,29.03)	0.0725 (0.0099,0.2508)	-7.36 (-8.71,-5.99)
Honduras	10.11 (1.72,30.65)	0.1042 (0.0178,0.3158)	1.2 (-0.85,3.29)
Hungary	0.19 (0.04,0.59)	0.0015 (3e-04,0.0047)	-6.55 (-8.67,-4.39)
Iceland	0 (0,0.02)	0.0012 (2e-04,0.0042)	-3.78 (-6.07,-1.44)
India	5939.39 (1330.89,14515.42)	0.4088 (0.0916,0.9994)	11.64 (6.44,17.09)
Indonesia	38.76 (5.21,145.96)	0.0126 (0.0017,0.0476)	-2.31 (-4.62,0.06)
Iran	49.9 (15.29,119.96)	0.05 (0.0153,0.12)	9.27 (6.96,11.63)
Iraq	0.11 (0.03,0.27)	3e-04 (1e-04,6e-04)	5.86 (3.03,8.76)
Ireland	0.37 (0.07,1.12)	0.0066 (0.0013,0.0201)	-2.63 (-3.51,-1.74)
Israel	0.69 (0.18,1.83)	0.0069 (0.0018,0.0185)	-5.44 (-6.37,-4.5)
Italy	3 (0.88,7.89)	0.0036 (0.0011,0.0095)	-5.91 (-7.66,-4.13)
Jamaica	0.64 (0.1,2.58)	0.0207 (0.0031,0.0838)	-1.38 (-2.64,-0.1)
Japan	1.91 (0.23,7.32)	0.0011 (1e-04,0.0041)	-4.6 (-6.19,-2.98)
Jordan	0.24 (0.04,0.87)	0.002 (3e-04,0.0073)	5.03 (2.36,7.78)
Kazakhstan	33.06 (15.37,56.88)	0.1589 (0.0739,0.2734)	4.76 (0.76,8.92)
Kenya	670.14 (152.87,1674.43)	1.7176 (0.3924,4.2712)	3.61 (-0.86,8.28)
Kiribati	0.01 (0,0.05)	0.0104 (0.0012,0.0434)	-1.85 (-3.62,-0.05)
Kuwait	0.07 (0.01,0.2)	0.0013 (3e-04,0.0037)	-0.11 (-1.75,1.57)
Kyrgyzstan	36.89 (12.13,75.5)	0.5569 (0.1831,1.1398)	15.48 (11.33,19.8)
Laos	2.18 (0.29,9.15)	0.0307 (0.0041,0.1288)	3.96 (0.02,8.06)
Latvia	6.5 (2.59,12.9)	0.3289 (0.131,0.653)	-3.45 (-4.09,-2.79)
Lebanon	0.58 (0.07,2.32)	0.0094 (0.0012,0.0376)	2.48 (0.45,4.55)
Lesotho	622.8 (173.26,1583.84)	37.7591 (10.634,96.1312)	11.87 (8.18,15.7)
Liberia	8.04 (1.25,25.73)	0.1835 (0.0285,0.5872)	-1.98 (-4.27,0.35)
Libya	3.58 (0.41,13.14)	0.0452 (0.0051,0.1657)	3.79 (1.45,6.19)
Lithuania	5.52 (2.54,9.36)	0.1867 (0.0858,0.3169)	3.92 (2.69,5.18)
Luxembourg	0.06 (0.01,0.2)	0.0068 (9e-04,0.0244)	1.94 (0.63,3.27)

Madagascar	15.29 (2.54,52.83)	0.0672 (0.0112,0.2322)	18.19 (13.14,23.46)
Malawi	787.1 (156.41,2257.07)	5.758 (1.1494,16.4951)	4.07 (0.03,8.29)
Malaysia	18.48 (4.21,54.36)	0.0529 (0.012,0.1554)	1.65 (-1.39,4.78)
Maldives	0.01 (0,0.04)	0.0014 (2e-04,0.0057)	-6 (-8.56,-3.36)
Mali	121.08 (18.52,349.89)	0.7221 (0.1104,2.0909)	1.66 (-0.54,3.91)
Malta	0.03 (0,0.12)	0.0061 (7e-04,0.0243)	-1.02 (-3.15,1.17)
Marshall Islands	0.04 (0.01,0.15)	0.07 (0.0091,0.2743)	10.07 (7.28,12.93)
Mauritania	4.21 (0.73,12.6)	0.1318 (0.0229,0.395)	-1.27 (-3.18,0.67)
Mauritius	0.07 (0.01,0.22)	0.0048 (9e-04,0.0154)	7.1 (4.06,10.22)
Mexico	99.77 (18.38,300.06)	0.0724 (0.0133,0.2178)	4.45 (1.45,7.53)
Moldova	40.87 (22.85,62.45)	0.9083 (0.5077,1.3881)	9.35 (6.91,11.85)
Monaco	0 (0,0.01)	0.0031 (4e-04,0.0114)	-1.95 (-2.68,-1.21)
Mongolia	0.75 (0.19,1.93)	0.0232 (0.0059,0.0598)	9.72 (8.15,11.31)
Montenegro	0.01 (0,0.02)	8e-04 (1e-04,0.0029)	-0.06 (-2.81,2.77)
Morocco	11.76 (2.99,30.29)	0.0299 (0.0076,0.0771)	4.48 (1.13,7.94)
Mozambique	4651.01 (1261.89,11314.92)	20.1074 (5.4377,48.9051)	7.29 (4.61,10.05)
Myanmar	659.71 (207.8,1558.97)	1.1456 (0.3609,2.7076)	5.33 (1.56,9.24)
Namibia	372.72 (128.98,851.17)	17.7696 (6.1381,40.6596)	11.4 (7.33,15.63)
Nauru	0 (0,0.01)	0.0125 (0.0012,0.0579)	4.09 (2.28,5.93)
Nepal	38.87 (9.32,100.43)	0.1348 (0.0323,0.3482)	15.97 (9.94,22.33)
Netherlands	1.33 (0.39,3.28)	0.0061 (0.0018,0.0149)	-2.39 (-3.21,-1.56)
New Zealand	0.17 (0.04,0.54)	0.0029 (6e-04,0.009)	-2.76 (-3.32,-2.19)
Nicaragua	1.64 (0.27,5.78)	0.0241 (0.004,0.085)	0.5 (-0.86,1.88)
Niger	53.53 (8.9,156.23)	0.3683 (0.0613,1.0749)	0.05 (-2.42,2.59)
Nigeria	2861.79 (737.21,7750.35)	1.7294 (0.4418,4.6764)	5.71 (2.05,9.5)
Niue	0 (0,0)	0.0044 (5e-04,0.0181)	4.05 (2.15,6)
North Korea	84.72 (25.29,202.24)	0.2721 (0.0812,0.6494)	13.3 (8.85,17.93)
North Macedonia	0.03 (0.01,0.08)	0.0011 (3e-04,0.0028)	0.17 (-1.74,2.12)
Northern Mariana Islands	0.01 (0,0.04)	0.0187 (0.0024,0.0744)	1.67 (-1.25,4.68)
Norway	0.28 (0.07,0.74)	0.0048 (0.0012,0.0126)	-4.33 (-5.14,-3.53)
Oman	0.21 (0.04,0.65)	0.0036 (6e-04,0.0113)	-3.07 (-4.41,-1.72)
Pakistan	210.93 (59.94,536.96)	0.0944 (0.0268,0.2401)	27.81 (23.79,31.97)
Palau	0 (0,0)	0.0052 (5e-04,0.0224)	5.1 (3.28,6.94)
Palestine	0.07 (0.01,0.26)	0.0017 (2e-04,0.0059)	2.36 (0.39,4.37)
Panama	5.95 (0.95,18.28)	0.1357 (0.0217,0.417)	1.02 (-1.47,3.56)
Papua New Guinea	51.76 (14.98,125.83)	0.5733 (0.1659,1.3931)	20.76 (16.54,25.14)
Paraguay	13.6 (1.91,43.23)	0.1854 (0.026,0.5895)	3.15 (0.71,5.64)
Peru	151.69 (61.34,323.94)	0.3966 (0.1604,0.8471)	1.22 (-1.13,3.63)
Philippines	22.48 (5.94,63.34)	0.0196 (0.0052,0.0548)	21.85 (16.63,27.31)
Poland	0.87 (0.23,2.44)	0.0019 (5e-04,0.0052)	0.07 (-2.53,2.74)
Portugal	3.26 (0.59,10.02)	0.0235 (0.0042,0.072)	-7.6 (-8.97,-6.21)
Puerto Rico	0.25 (0.05,0.77)	0.006 (0.0011,0.0186)	-6.72 (-8.66,-4.75)
Qatar	0.06 (0.01,0.18)	0.0015 (3e-04,0.0049)	-0.56 (-1.65,0.54)
Romania	6.45 (2.02,15.19)	0.0351 (0.011,0.0826)	-0.64 (-2,0.74)

Russia	2220.07 (1163.8,3608.65)	1.3849 (0.7267,2.2487)	7.5 (4.51,10.57)
Rwanda	232.36 (75.67,534.44)	2.2625 (0.7393,5.2005)	4.44 (0.3,8.75)
Saint Kitts and Nevis	0.01 (0,0.03)	0.0115 (0.0016,0.0422)	-5.36 (-6.88,-3.82)
Saint Lucia	0.02 (0,0.06)	0.0072 (0.0011,0.0264)	-10.97 (-12.53,-9.38)
Saint Vincent and the Grenadines	0.02 (0,0.07)	0.0165 (0.0022,0.0582)	-10.2 (-11.52,-8.85)
Samoa	0 (0,0.01)	0.0014 (2e-04,0.0053)	1.69 (-0.89,4.34)
San Marino	0 (0,0)	0.0017 (3e-04,0.0062)	-0.65 (-1.1,-0.19)
São Tomé and Príncipe	0.38 (0.06,1.21)	0.2184 (0.0328,0.6925)	-0.99 (-2.97,1.03)
Saudi Arabia	12.27 (2.43,39.57)	0.0268 (0.0053,0.0866)	4.64 (1.61,7.76)
Senegal	31.74 (7.99,79.59)	0.2596 (0.0653,0.6509)	3.06 (-0.38,6.62)
Serbia	0.83 (0.19,2.22)	0.0087 (0.002,0.0234)	-0.45 (-1.88,1)
Seychelles	0 (0,0.01)	0.0027 (3e-04,0.0107)	-1.68 (-4.82,1.57)
Sierra Leone	40.06 (6.89,127.01)	0.5961 (0.1026,1.8901)	3.16 (0.77,5.6)
Singapore	0.78 (0.25,1.96)	0.0099 (0.0031,0.0248)	1.21 (0.43,2)
Slovakia	0.01 (0,0.02)	1e-04 (0,3e-04)	-4.37 (-5.73,-2.99)
Slovenia	0 (0,0)	0 (0,2e-04)	-12.94 (-14.85,-10.99)
Solomon Islands	0.07 (0.01,0.27)	0.0103 (9e-04,0.0429)	5.82 (4.11,7.55)
Somalia	258.55 (63.26,718.06)	1.8409 (0.4505,5.1105)	14.89 (9.42,20.63)
South Africa	8992.99 (2472.7,23010.69)	14.8552 (4.0897,37.8753)	6.88 (3.19,10.71)
South Korea	7.53 (1.08,28.28)	0.0107 (0.0015,0.0402)	-2.28 (-4.1,-0.42)
South Sudan	197.72 (36.46,591.3)	2.7932 (0.5151,8.3536)	10.04 (6.6,13.59)
Spain	3.6 (0.53,14.65)	0.0054 (8e-04,0.0219)	-9.24 (-10.17,-8.29)
Sri Lanka	0.23 (0.03,0.9)	0.001 (1e-04,0.0038)	-1.52 (-3.84,0.87)
Sudan	32.91 (4.28,127.87)	0.0982 (0.0128,0.3807)	3.16 (0.14,6.26)
Suriname	0.13 (0.02,0.5)	0.0207 (0.003,0.0818)	-6.47 (-7.62,-5.31)
Sweden	0.78 (0.22,1.94)	0.0069 (0.0019,0.0171)	-0.5 (-1.28,0.28)
Switzerland	0.92 (0.29,2.34)	0.0077 (0.0025,0.0197)	-1.39 (-2.47,-0.29)
Syria	0.2 (0.03,0.72)	0.0014 (2e-04,0.005)	1.68 (-0.86,4.28)
Taiwan (province of China)	3.36 (0.49,12.12)	0.0116 (0.0017,0.0417)	-1.59 (-3.79,0.65)
Tajikistan	13.1 (5.02,24.76)	0.1404 (0.0538,0.2654)	10.12 (6.15,14.24)
Tanzania	981.16 (170.6,3338.08)	2.2154 (0.3855,7.558)	1.95 (-1.27,5.27)
Thailand	481.83 (114.08,1282.25)	0.5962 (0.1411,1.5805)	3.57 (1.96,5.2)
The Bahamas	0.22 (0.03,0.86)	0.0509 (0.0067,0.1958)	2.19 (0.57,3.84)
The Gambia	19.81 (3.17,59.97)	1.0989 (0.1759,3.3254)	4.8 (2.27,7.38)
Timor-Leste	0.27 (0.04,1.05)	0.0239 (0.0031,0.0922)	-1.36 (-5.29,2.74)
Togo	62.4 (9.01,203.12)	0.9255 (0.1337,3.0117)	1.72 (-0.84,4.34)
Tokelau	0 (0,0)	0.0037 (4e-04,0.0145)	2.99 (1.05,4.97)
Tonga	0 (0,0.01)	0.0018 (2e-04,0.0089)	-0.06 (-1.84,1.76)
Trinidad and Tobago	0.23 (0.03,0.86)	0.0148 (0.0019,0.0541)	-6.25 (-7.72,-4.77)
Tunisia	1.12 (0.31,3.04)	0.0084 (0.0023,0.0227)	6.97 (3.77,10.27)
Türkiye	4.12 (1.43,8.78)	0.0046 (0.0016,0.0098)	6.84 (3.87,9.89)
Turkmenistan	17.02 (5.36,35.58)	0.3208 (0.1009,0.6706)	10.84 (6.76,15.08)
Tuvalu	0 (0,0.01)	0.0173 (0.0017,0.076)	2.88 (1.27,4.51)
Uganda	1861.11 (467.71,4820.22)	6.4448 (1.6145,16.6907)	4.37 (1.13,7.7)

UK	4 (0.95,11.43)	0.0051 (0.0012,0.0148)	-3.33 (-3.98,-2.68)
Ukraine	1110.8 (499.15,1948.88)	2.1829 (0.9808,3.8297)	9.09 (6.2,12.06)
United Arab Emirates	0.13 (0.02,0.52)	8e-04 (1e-04,0.0034)	1.17 (-1.31,3.72)
Uruguay	0.53 (0.1,1.72)	0.0148 (0.0027,0.0485)	2.67 (1.52,3.84)
USA	11.12 (3.59,29.93)	0.0027 (9e-04,0.0072)	-9.74 (-10.8,-8.66)
Uzbekistan	102.83 (34.47,209.93)	0.2771 (0.0929,0.5657)	14.88 (10.69,19.22)
Vanuatu	0.02 (0,0.08)	0.0069 (8e-04,0.0262)	10.39 (8.97,11.82)
Venezuela	20.27 (3.27,69.66)	0.0702 (0.0113,0.2411)	1.66 (-0.27,3.63)
Viet Nam	476.76 (123.38,1211.3)	0.4295 (0.1111,1.0914)	7.15 (4.79,9.57)
Virgin Islands	0.01 (0,0.02)	0.0059 (8e-04,0.0225)	-5.86 (-7.3,-4.39)
Yemen	6.94 (1.33,24.3)	0.0244 (0.0047,0.0856)	4.75 (2.2,7.37)
Zambia	1557.51 (259.4,4979.27)	11.0858 (1.8383,35.4805)	1.56 (-1.11,4.3)
Zimbabwe	2168.7 (352.6,6926.19)	17.9387 (2.8979,57.1174)	4.25 (1.81,6.74)

Abbreviations: MDR-TB=multidrug-resistant tuberculosis. XDR-TB=extensively drug-resistant tuberculosis, ASR=age-standardized rate. EAPC=estimated annual percentage change.

Table S11 The death of cases and ASR of HIV/AIDS-MDR-TB in 2021, and the trends from baseline to 2021 by countries.

Location	Deaths_Number_2021	Deaths_ASR_2021	EAPC
Afghanistan	1.42 (0.19,5.18)	0.0062 (8e-04,0.0227)	5.44 (1.98,9.02)
Albania	0.01 (0,0.02)	2e-04 (0,9e-04)	-0.67 (-3.01,1.73)
Algeria	3.17 (0.33,12.54)	0.0068 (7e-04,0.0266)	3 (2.24,3.77)
American Samoa	0 (0,0)	3e-04 (0,0.0014)	-1.16 (-2.73,0.44)
Andorra	0 (0,0)	1e-04 (0,4e-04)	-2.07 (-2.73,-1.4)
Angola	243.54 (32.19,782.5)	1.0209 (0.1353,3.2938)	11.29 (7.51,15.21)
Antigua and Barbuda	0 (0,0.01)	0.0017 (2e-04,0.007)	-10.14 (-11.43,-8.83)
Argentina	19.47 (2.54,73.24)	0.039 (0.0051,0.1468)	0.21 (-2.45,2.93)
Armenia	0.98 (0.22,2.17)	0.028 (0.0064,0.0622)	13.79 (9.95,17.77)
Australia	0.38 (0.09,1.05)	0.0012 (3e-04,0.0034)	-2.1 (-3.24,-0.95)
Austria	0.37 (0.08,0.99)	0.0035 (8e-04,0.0093)	-1.27 (-2.34,-0.19)
Azerbaijan	3.79 (1.09,8.45)	0.0305 (0.0088,0.0685)	10.64 (6.34,15.11)
Bahrain	0.06 (0.01,0.23)	0.003 (4e-04,0.0116)	-1.29 (-4.3,1.82)
Bangladesh	18.15 (3.95,51.7)	0.0108 (0.0024,0.0304)	32.06 (26.01,38.4)
Barbados	0.01 (0,0.02)	0.0014 (1e-04,0.0066)	-13.75 (-15.81,-11.65)

Belarus	8.68 (4.67,14.62)	0.0806 (0.0434,0.1359)	5.81 (2.37,9.37)
Belgium	0.42 (0.08,1.23)	0.0032 (6e-04,0.0092)	-5.63 (-6.42,-4.83)
Belize	0.08 (0.01,0.31)	0.018 (0.0016,0.075)	-6.94 (-8.46,-5.4)
Benin	22.75 (3.37,75.3)	0.251 (0.0372,0.8294)	7.42 (2.19,12.92)
Bermuda	0 (0,0)	2e-04 (0,0.0011)	-11.41 (-13.25,-9.54)
Bhutan	0.12 (0.01,0.45)	0.0147 (0.0018,0.0546)	1.62 (-1.12,4.44)
Bolivia	7.6 (1.05,24.09)	0.0654 (0.009,0.207)	6.3 (1.58,11.23)
Bosnia and Herzegovina	0.01 (0,0.05)	4e-04 (1e-04,0.0014)	-0.77 (-1.59,0.05)
Botswana	210.61 (37.99,645.51)	9.1987 (1.6605,28.4191)	8.27 (5.01,11.63)
Brazil	181.95 (28.93,576.81)	0.0723 (0.0115,0.229)	5.94 (2.63,9.35)
Brunei	0 (0,0.01)	6e-04 (1e-04,0.0022)	-2.23 (-3.52,-0.92)
Bulgaria	0.03 (0,0.08)	4e-04 (1e-04,0.0012)	1.08 (-1.67,3.91)
Burkina Faso	76.61 (12.1,214.24)	0.5138 (0.0824,1.4087)	-3.82 (-5.64,-1.96)
Burundi	65.05 (9.24,215.7)	0.728 (0.0968,2.3952)	-0.12 (-4.14,4.07)
Cabo Verde	0.68 (0.1,2.33)	0.121 (0.0172,0.415)	-1.34 (-3.83,1.22)
Cambodia	7.61 (0.93,29.82)	0.0466 (0.0058,0.1808)	16.08 (3.96,29.61)
Cameroon	198.94 (27.58,700.63)	0.8781 (0.1216,3.0843)	3.57 (0.1,7.16)
Canada	0.87 (0.2,2.56)	0.0019 (4e-04,0.0056)	-7.65 (-8.45,-6.84)
Central African Republic	71.99 (9.42,268.34)	1.7262 (0.2329,6.1028)	-0.13 (-2.92,2.75)
Chad	80.24 (12.49,254.15)	0.7547 (0.1123,2.37)	3.33 (0.27,6.48)
Chile	5.17 (1.14,15.42)	0.0238 (0.0052,0.071)	3.86 (2.02,5.72)
China	291.83 (39.45,922.25)	0.0164 (0.0022,0.0516)	-0.75 (-3.07,1.63)
Colombia	29.05 (4.46,88.3)	0.0539 (0.0083,0.1639)	4.4 (2.38,6.46)
Comoros	0.1 (0.01,0.28)	0.0138 (0.0016,0.0405)	12.9 (8.84,17.11)
Congo (Brazzaville)	46.15 (5.44,188.57)	0.9681 (0.115,3.9606)	-1.06 (-3.28,1.21)
Cook Islands	0 (0,0)	0.001 (1e-04,0.0042)	2 (0.39,3.64)
Costa Rica	0.56 (0.07,2.02)	0.0105 (0.0013,0.038)	0.33 (-2.54,3.27)
Côte d'Ivoire	149.92 (22.2,482)	0.733 (0.1097,2.3452)	-6.17 (-8.07,-4.22)
Croatia	0.01 (0,0.03)	2e-04 (0,8e-04)	-5.32 (-7.08,-3.54)
Cuba	0.31 (0.05,1)	0.0023 (3e-04,0.0074)	-1.26 (-2.56,0.06)
Cyprus	0.02 (0,0.07)	0.0012 (2e-04,0.0041)	-0.94 (-3.53,1.72)
Czechia	0.09 (0.02,0.24)	8e-04 (2e-04,0.0022)	-0.18 (-1.26,0.92)
Denmark	0.07 (0.01,0.23)	0.001 (2e-04,0.0033)	-1.14 (-2.95,0.72)
Djibouti	16.86 (4.3,43.03)	1.4202 (0.3629,3.6086)	23.31 (14.31,33.03)
Dominica	0.01 (0,0.04)	0.0137 (0.0015,0.0526)	-8.92 (-10.35,-7.47)
Dominican Republic	2 (0.19,8.07)	0.0179 (0.0017,0.0725)	-6.28 (-8.68,-3.82)
DR Congo	169.56 (24.08,589.57)	0.2412 (0.0348,0.8532)	-1.58 (-4.2,1.11)
Ecuador	23.85 (3.26,77.79)	0.1327 (0.0181,0.4327)	7.58 (3.4,11.94)
Egypt	1.96 (0.34,5.93)	0.002 (4e-04,0.0061)	6.44 (3.63,9.33)
El Salvador	2.07 (0.25,7.36)	0.033 (0.004,0.1176)	-1.18 (-4.27,2.01)
Equatorial Guinea	9.34 (1.15,31.69)	0.8039 (0.0991,2.726)	5.38 (1.75,9.13)
Eritrea	37.42 (5.32,114.88)	0.7116 (0.1052,2.1717)	5.13 (0.95,9.47)
Estonia	1.35 (0.54,2.64)	0.0964 (0.0383,0.1883)	4.46 (1.83,7.15)
Eswatini	283.44 (67.21,687.22)	27.9755 (6.3501,68.607)	19.31 (11.73,27.4)

Ethiopia	348.1 (52.42,1091.62)	0.4559 (0.0681,1.4773)	3.02 (-1.49,7.73)
Federated States of Micronesia	0.01 (0,0.03)	0.0073 (6e-04,0.0336)	7.57 (5.4,9.77)
Fiji	0.01 (0,0.04)	0.001 (1e-04,0.0043)	4.36 (3.22,5.52)
Finland	0.18 (0.04,0.48)	0.003 (6e-04,0.0081)	3.72 (2.97,4.47)
France	5.76 (1.15,17.78)	0.0072 (0.0014,0.0222)	-6.93 (-7.7,-6.15)
Gabon	19.83 (2.52,72.9)	1.2872 (0.1633,4.7387)	4.39 (1.42,7.44)
Georgia	3.33 (1.34,6.11)	0.0839 (0.0338,0.154)	14.15 (11.21,17.16)
Germany	5.05 (1.24,13.04)	0.0045 (0.0011,0.0115)	-3.67 (-4.52,-2.82)
Ghana	245.11 (27.71,855.93)	0.8762 (0.0991,3.064)	2.94 (0.31,5.64)
Greece	0.59 (0.09,1.84)	0.005 (8e-04,0.0159)	2.37 (0.93,3.82)
Greenland	0.02 (0,0.07)	0.0292 (0.0035,0.1028)	-5.61 (-6.39,-4.81)
Grenada	0 (0,0.01)	0.0014 (2e-04,0.0066)	-8.09 (-9.29,-6.87)
Guam	0 (0,0.02)	0.0024 (2e-04,0.0103)	-0.56 (-2.6,1.52)
Guatemala	11.45 (1.67,35.38)	0.0797 (0.0116,0.2467)	0.24 (-3.03,3.62)
Guinea	65.75 (9.32,222.08)	0.7306 (0.1036,2.4397)	5.53 (2.28,8.89)
Guinea-Bissau	29.16 (3.03,108.65)	1.8721 (0.2011,6.8603)	9.59 (6.02,13.27)
Guyana	0.43 (0.04,1.75)	0.0567 (0.0055,0.2296)	-2.81 (-4.72,-0.86)
Haiti	5.58 (0.52,25.72)	0.0498 (0.0046,0.2307)	-7.73 (-9.07,-6.37)
Honduras	5.45 (0.79,17.71)	0.0566 (0.0082,0.1839)	2.44 (0.09,4.84)
Hungary	0.05 (0.01,0.19)	5e-04 (1e-04,0.0018)	-9.37 (-11.49,-7.19)
Iceland	0 (0,0.01)	5e-04 (1e-04,0.002)	-5.7 (-8.01,-3.32)
India	1552.47 (319.88,3853.27)	0.1071 (0.0218,0.2647)	17.27 (8.9,26.29)
Indonesia	15.62 (1.7,61.5)	0.005 (5e-04,0.0198)	2.36 (-0.59,5.39)
Iran	11.27 (2.68,28.8)	0.0113 (0.0027,0.0289)	14.89 (11.68,18.19)
Iraq	0.04 (0.01,0.12)	1e-04 (0,3e-04)	3.29 (0.44,6.22)
Ireland	0.15 (0.02,0.49)	0.0027 (4e-04,0.0089)	-2.36 (-3.51,-1.2)
Israel	0.4 (0.08,1.08)	0.0042 (8e-04,0.0113)	-6.24 (-7.18,-5.3)
Italy	1.06 (0.21,2.97)	0.0013 (3e-04,0.0038)	-7.74 (-9.14,-6.33)
Jamaica	0.42 (0.05,1.81)	0.0137 (0.0015,0.0593)	-1.4 (-2.81,0.03)
Japan	1.24 (0.12,4.96)	7e-04 (1e-04,0.0029)	-1.5 (-3.62,0.68)
Jordan	0.07 (0.01,0.24)	6e-04 (1e-04,0.002)	0.85 (-1.97,3.75)
Kazakhstan	6.37 (2.64,11.57)	0.0307 (0.0127,0.0558)	1.68 (-3.07,6.66)
Kenya	467.28 (86.06,1273.2)	1.196 (0.217,3.3468)	4.36 (-0.5,9.46)
Kiribati	0.01 (0,0.03)	0.0066 (5e-04,0.0274)	-1.61 (-3.52,0.34)
Kuwait	0.02 (0,0.05)	3e-04 (0,9e-04)	-0.15 (-2.32,2.07)
Kyrgyzstan	7.35 (2.47,14.1)	0.1081 (0.0363,0.2074)	11.71 (6.69,16.97)
Laos	1.36 (0.13,5.21)	0.019 (0.0018,0.0735)	19.1 (12.04,26.6)
Latvia	1.81 (0.56,3.77)	0.091 (0.0284,0.1898)	-3.88 (-5.09,-2.66)
Lebanon	0.21 (0.02,0.8)	0.0035 (3e-04,0.0131)	1.38 (-0.93,3.74)
Lesotho	352.94 (86.44,939.8)	21.9686 (5.2292,59.1373)	15.86 (10.36,21.64)
Liberia	4.62 (0.59,17.06)	0.1102 (0.0144,0.4045)	-0.82 (-3.86,2.33)
Libya	0.44 (0.01,2.51)	0.0058 (2e-04,0.0322)	4.07 (1.19,7.04)
Lithuania	1.19 (0.48,2.19)	0.043 (0.0172,0.0792)	1.47 (-0.45,3.43)
Luxembourg	0.01 (0,0.03)	0.0011 (1e-04,0.0039)	-1.87 (-2.98,-0.75)

Madagascar	11.22 (1.33,39.9)	0.0504 (0.006,0.1807)	23.06 (15.81,30.76)
Malawi	408.09 (72.28,1251.75)	3.049 (0.5636,9.2615)	3.93 (-0.77,8.85)
Malaysia	9.29 (1.57,29.19)	0.027 (0.0045,0.0848)	3.53 (-0.63,7.86)
Maldives	0 (0,0.02)	7e-04 (1e-04,0.0028)	-4.82 (-8.44,-1.06)
Mali	71.93 (9.21,229.9)	0.4351 (0.0555,1.4063)	3.44 (0.34,6.65)
Malta	0 (0,0.01)	3e-04 (0,0.0011)	-4.39 (-6.59,-2.14)
Marshall Islands	0.02 (0,0.09)	0.0364 (0.0039,0.155)	15.19 (11.76,18.74)
Mauritania	0.07 (0.01,0.26)	0.0023 (3e-04,0.0085)	0.58 (-2.06,3.28)
Mauritius	0.03 (0,0.11)	0.0018 (2e-04,0.007)	6.94 (3.93,10.03)
Mexico	47.84 (8.01,149.97)	0.0346 (0.0058,0.1085)	3.47 (0.26,6.78)
Moldova	4.44 (2.33,7.17)	0.1027 (0.0539,0.166)	5.5 (1.87,9.27)
Monaco	0 (0,0.01)	0.0024 (2e-04,0.0104)	-1.42 (-2.28,-0.55)
Mongolia	0.35 (0.07,1)	0.0098 (0.0018,0.0275)	9.7 (7.83,11.61)
Montenegro	0 (0,0.01)	4e-04 (0,0.0016)	-0.29 (-2.89,2.38)
Morocco	6.12 (1.27,16.67)	0.0155 (0.0032,0.0422)	6.97 (2.81,11.29)
Mozambique	2321.13 (546.97,5904.22)	9.5174 (2.2129,24.1161)	12.12 (7.54,16.89)
Myanmar	178.34 (45.16,448.74)	0.3074 (0.078,0.7715)	13.78 (7.05,20.92)
Namibia	160.76 (47.68,376.11)	7.6308 (2.2574,18.1136)	13.73 (8.02,19.74)
Nauru	0 (0,0)	0.0077 (5e-04,0.0377)	8.34 (6.03,10.71)
Nepal	24.44 (5.07,67.7)	0.0845 (0.0174,0.2345)	28.2 (17.66,39.68)
Netherlands	0.78 (0.17,2.15)	0.0038 (8e-04,0.0104)	-3.47 (-4.29,-2.63)
New Zealand	0.01 (0,0.04)	2e-04 (0,7e-04)	-8.5 (-9.91,-7.06)
Nicaragua	1 (0.13,3.61)	0.015 (0.002,0.0545)	1.26 (-0.02,2.56)
Niger	25.92 (3.47,85.03)	0.1837 (0.0245,0.6034)	1.92 (-1.64,5.61)
Nigeria	1264.96 (255.27,3414.12)	0.7746 (0.1578,2.121)	7.08 (2.64,11.71)
Niue	0 (0,0)	0.0034 (3e-04,0.0155)	4.96 (2.75,7.22)
North Korea	17.72 (2.44,60.27)	0.06 (0.0087,0.2056)	22.49 (15.96,29.38)
North Macedonia	0.01 (0,0.03)	4e-04 (1e-04,0.0012)	1.74 (-0.44,3.96)
Northern Mariana Islands	0 (0,0.01)	0.0067 (7e-04,0.026)	-0.45 (-2.96,2.12)
Norway	0.16 (0.03,0.51)	0.0026 (5e-04,0.0082)	-5.65 (-6.56,-4.74)
Oman	0.04 (0.01,0.13)	8e-04 (1e-04,0.0029)	-5.39 (-6.73,-4.04)
Pakistan	95.94 (6.43,323.46)	0.0441 (0.0031,0.1517)	46.09 (39.33,53.17)
Palau	0 (0,0)	0.0041 (4e-04,0.0188)	5.73 (3.71,7.79)
Palestine	0.02 (0,0.08)	5e-04 (0,0.0019)	0.13 (-2.34,2.66)
Panama	2.99 (0.4,9.73)	0.0686 (0.0091,0.2232)	-0.15 (-2.79,2.56)
Papua New Guinea	28.52 (6.25,72.54)	0.3262 (0.071,0.846)	30.34 (22.59,38.59)
Paraguay	7.79 (0.95,24.59)	0.1094 (0.0134,0.3442)	6.06 (3.26,8.93)
Peru	57.19 (16.54,135.68)	0.1519 (0.0439,0.3603)	2.58 (-0.65,5.91)
Philippines	6.79 (1.45,19.64)	0.006 (0.0013,0.0172)	20.9 (15.09,27.01)
Poland	0.28 (0.06,0.84)	6e-04 (1e-04,0.0019)	1.67 (-1.83,5.29)
Portugal	2.26 (0.31,7.33)	0.0168 (0.0023,0.0547)	-7.09 (-8.74,-5.4)
Puerto Rico	0.19 (0.03,0.69)	0.0046 (7e-04,0.0168)	-6.73 (-8.68,-4.73)
Qatar	0.01 (0,0.02)	2e-04 (0,6e-04)	-6.43 (-8.18,-4.65)
Romania	2.52 (0.56,6.32)	0.0137 (0.003,0.0344)	-1.59 (-3.08,-0.07)

Russia	302.55 (147.66,528.48)	0.1816 (0.0886,0.3172)	5.55 (2.12,9.09)
Rwanda	124.9 (29.94,322.35)	1.2271 (0.298,3.1748)	5.7 (0.46,11.21)
Saint Kitts and Nevis	0.01 (0,0.02)	0.0079 (9e-04,0.0306)	-6.27 (-7.87,-4.64)
Saint Lucia	0.01 (0,0.04)	0.0052 (6e-04,0.0207)	-11.15 (-12.7,-9.58)
Saint Vincent and the Grenadines	0.01 (0,0.05)	0.0098 (0.0011,0.0398)	-10.7 (-11.96,-9.42)
Samoa	0 (0,0.01)	0.001 (1e-04,0.0044)	2.53 (-0.48,5.62)
San Marino	0 (0,0)	0.0014 (2e-04,0.0052)	-0.28 (-0.74,0.19)
São Tomé and Príncipe	0.01 (0,0.02)	0.0036 (4e-04,0.0128)	-0.86 (-3.64,2)
Saudi Arabia	4.3 (0.68,13.93)	0.009 (0.0014,0.0293)	3.19 (-0.37,6.87)
Senegal	16.72 (3.46,45.23)	0.1378 (0.0285,0.3736)	4.82 (0.49,9.33)
Serbia	0.26 (0.05,0.75)	0.0027 (5e-04,0.0079)	0.75 (-0.98,2.5)
Seychelles	0 (0,0.01)	0.0014 (1e-04,0.0061)	-2.8 (-6.42,0.97)
Sierra Leone	25.78 (3.7,89.88)	0.3928 (0.0563,1.3735)	6.03 (2.67,9.51)
Singapore	0.13 (0.03,0.39)	0.0017 (4e-04,0.0049)	-2.44 (-3.71,-1.14)
Slovakia	0 (0,0.01)	0 (0,2e-04)	-5.62 (-6.8,-4.42)
Slovenia	0 (0,0)	0 (0,1e-04)	-14.02 (-16.1,-11.9)
Solomon Islands	0.05 (0,0.21)	0.0071 (6e-04,0.0327)	7.53 (5.24,9.87)
Somalia	172.08 (35.91,525.31)	1.3115 (0.2614,4.0728)	21.07 (13.13,29.56)
South Africa	2926.64 (662.59,7577.46)	4.8292 (1.0948,12.5302)	9.31 (3.84,15.07)
South Korea	1.44 (0.17,5.61)	0.0019 (2e-04,0.0073)	-0.35 (-2.55,1.91)
South Sudan	117.17 (18.06,356.69)	1.7069 (0.2632,5.1734)	12.11 (7.58,16.83)
Spain	1.82 (0.2,7)	0.003 (3e-04,0.0116)	-10.17 (-11.18,-9.14)
Sri Lanka	0.09 (0.01,0.36)	4e-04 (0,0.0015)	-3.73 (-6.37,-1.01)
Sudan	11.79 (1.25,46.35)	0.0425 (0.0045,0.1669)	2.56 (-0.96,6.21)
Suriname	0.07 (0.01,0.31)	0.0119 (0.0013,0.0503)	-6.87 (-8.03,-5.69)
Sweden	0.28 (0.06,0.78)	0.0024 (5e-04,0.0067)	-3.35 (-4.28,-2.41)
Switzerland	0.26 (0.06,0.7)	0.0025 (6e-04,0.0066)	-2.81 (-5.13,-0.43)
Syria	0.06 (0.01,0.25)	4e-04 (0,0.0018)	0.1 (-2.57,2.84)
Taiwan (province of China)	1.21 (0.16,4.5)	0.0039 (5e-04,0.0144)	-1.37 (-4.13,1.47)
Tajikistan	5.09 (1.68,10.3)	0.0518 (0.0171,0.1048)	8.97 (4.36,13.78)
Tanzania	503.53 (71.22,1843.42)	1.1557 (0.1639,4.2323)	2.52 (-1.3,6.5)
Thailand	220.04 (40.86,638.77)	0.2562 (0.0479,0.7442)	5.99 (4.64,7.36)
The Bahamas	0.15 (0.02,0.66)	0.0345 (0.0036,0.1491)	1.95 (0.26,3.66)
The Gambia	10.87 (1.47,38.9)	0.6354 (0.0855,2.2523)	7.83 (4.11,11.67)
Timor-Leste	0.21 (0.02,0.92)	0.0187 (0.0015,0.0815)	-0.46 (-4.91,4.19)
Togo	37.9 (4.97,124.57)	0.5645 (0.074,1.8578)	2.58 (-0.6,5.86)
Tokelau	0 (0,0)	0.0025 (2e-04,0.0107)	4.19 (1.83,6.6)
Tonga	0 (0,0)	0.001 (1e-04,0.0048)	-0.11 (-2.03,1.85)
Trinidad and Tobago	0.14 (0.02,0.54)	0.0083 (9e-04,0.0329)	-7.4 (-9,-5.77)
Tunisia	0.54 (0.1,1.76)	0.004 (7e-04,0.0129)	13.64 (9.32,18.12)
Türkiye	1.41 (0.37,3.42)	0.0015 (4e-04,0.0037)	6.95 (3.34,10.68)
Turkmenistan	7.09 (2.07,14.87)	0.1333 (0.039,0.2798)	9.8 (5.1,14.7)
Tuvalu	0 (0,0)	0.0076 (5e-04,0.034)	8.34 (6.08,10.66)
Uganda	845.8 (161.36,2384.45)	2.9315 (0.565,8.2561)	3.02 (-0.46,6.62)

UK	1.27 (0.25,3.61)	0.0017 (3e-04,0.0047)	-4.84 (-5.68,-4)
Ukraine	235.03 (101.4,404.62)	0.4573 (0.1973,0.7872)	7.08 (3.64,10.64)
United Arab Emirates	0.05 (0,0.19)	3e-04 (0,0.0013)	0.08 (-2.31,2.53)
Uruguay	0.17 (0.02,0.59)	0.0046 (6e-04,0.0156)	0.5 (-0.83,1.84)
USA	5.44 (1.25,17.16)	0.0013 (3e-04,0.004)	-11.05 (-12.5,-9.57)
Uzbekistan	20.48 (6.57,39.48)	0.0559 (0.018,0.1079)	11.96 (6.94,17.22)
Vanuatu	0.01 (0,0.07)	0.0051 (4e-04,0.0235)	11.78 (10,13.58)
Venezuela	13.92 (1.7,51.38)	0.0474 (0.0058,0.1749)	0.99 (-1.02,3.04)
Viet Nam	175.59 (31.91,499.31)	0.1564 (0.0285,0.4452)	10.88 (7.14,14.75)
Virgin Islands	0 (0,0.02)	0.0043 (4e-04,0.0178)	-5.3 (-6.67,-3.9)
Yemen	4.77 (0.68,17.08)	0.0169 (0.0024,0.0617)	7.4 (4.01,10.89)
Zambia	482.1 (64.71,1558.68)	3.4991 (0.4686,11.3156)	-0.09 (-3.41,3.34)
Zimbabwe	942.45 (144.1,2940.96)	8.1044 (1.2461,25.313)	3.46 (0.26,6.75)

Abbreviations: MDR-TB=multidrug-resistant tuberculosis. XDR-TB=extensively drug-resistant tuberculosis. ASR=age-standardized rate. EAPC=estimated annual percentage change.

Table S12 The incidence of cases and ASR of HIV/AIDS-XDR-TB in 2021, and the trends from baseline to 2021 by countries.

Location	Incidence_Number_2021	Incidence_ASR_2021	EAPC
Afghanistan	0.1359 (0.0185,0.4407)	6e-04 (1e-04,0.0019)	10.69 (6.52,15.03)
Albania	0.0112 (0.0019,0.036)	4e-04 (1e-04,0.0013)	5.58 (3.11,8.12)
Algeria	0.3033 (0.0353,1.2101)	7e-04 (1e-04,0.0026)	10.55 (9.06,12.05)
American Samoa	1e-04 (0,2e-04)	1e-04 (0,5e-04)	6.18 (4.06,8.34)
Andorra	0 (0,2e-04)	0 (0,1e-04)	1.14 (-0.7,3.02)
Angola	4.2606 (0.64,12.7303)	0.0179 (0.0027,0.0533)	15.28 (11.58,19.11)
Antigua and Barbuda	0.001 (1e-04,0.0044)	0.001 (1e-04,0.0042)	-0.55 (-2.05,0.98)
Argentina	6.7353 (1.0796,24.086)	0.0135 (0.0022,0.0481)	5.91 (2.87,9.03)
Armenia	1.221 (0.2803,2.8501)	0.0345 (0.0079,0.0805)	17.27 (13.25,21.43)
Australia	0.1711 (0.053,0.4312)	5e-04 (2e-04,0.0013)	8.71 (8.09,9.32)
Austria	0.1311 (0.0409,0.3058)	0.0012 (4e-04,0.0029)	8.34 (6.38,10.34)
Azerbaijan	4.1524 (1.2002,8.6716)	0.0336 (0.0097,0.0701)	16.39 (12.4,20.52)
Bahrain	0.0073 (0.0011,0.0296)	4e-04 (1e-04,0.0016)	5.03 (1.87,8.29)
Bangladesh	3.22 (0.9002,8.6232)	0.0019 (5e-04,0.0051)	20.46 (16.52,24.53)
Barbados	0.0011 (1e-04,0.0046)	3e-04 (0,0.0013)	-7.19 (-9.4,-4.92)

Belarus	9.6694 (5.8282,15.0953)	0.0912 (0.0549,0.1423)	13.46 (9.97,17.06)
Belgium	0.1549 (0.0373,0.4221)	0.0011 (3e-04,0.0031)	2.75 (1.39,4.13)
Belize	0.0165 (0.0019,0.0663)	0.0039 (5e-04,0.0158)	0.18 (-2.02,2.42)
Benin	0.4022 (0.0751,1.1576)	0.0044 (8e-04,0.0128)	9.55 (4.86,14.45)
Bermuda	6e-04 (0,0.003)	8e-04 (1e-04,0.0037)	-0.93 (-2.79,0.97)
Bhutan	0.0082 (0.0014,0.0252)	0.001 (2e-04,0.0031)	7.33 (4.35,10.4)
Bolivia	1.6154 (0.2738,5.1923)	0.0139 (0.0024,0.0447)	9.25 (4.8,13.89)
Bosnia and Herzegovina	0.0121 (0.0026,0.0373)	3e-04 (1e-04,0.001)	8.15 (6.24,10.09)
Botswana	3.7054 (0.7639,10.9752)	0.162 (0.0332,0.4795)	13.99 (9.83,18.32)
Brazil	46.9423 (9.2352,134.2383)	0.0189 (0.0037,0.0541)	11.33 (8.16,14.58)
Brunei	0.0014 (1e-04,0.0052)	3e-04 (0,0.0011)	6.42 (4.89,7.97)
Bulgaria	0.0184 (0.0041,0.0534)	3e-04 (1e-04,8e-04)	8.17 (4.91,11.53)
Burkina Faso	1.3429 (0.2355,3.6798)	0.009 (0.0016,0.0247)	2.55 (0.08,5.08)
Burundi	1.1555 (0.1925,3.6081)	0.0129 (0.0021,0.0404)	2.84 (-0.75,6.57)
Cabo Verde	0.0119 (0.0019,0.0343)	0.0021 (3e-04,0.0061)	4 (1.2,6.88)
Cambodia	2.0324 (0.3171,6.9361)	0.0126 (0.002,0.0431)	3.78 (-1.56,9.4)
Cameroon	3.4249 (0.5709,9.9164)	0.0151 (0.0025,0.0438)	8.03 (4.28,11.91)
Canada	0.3178 (0.0948,0.8201)	7e-04 (2e-04,0.0018)	1.45 (0.53,2.38)
Central African Republic	1.2596 (0.2022,4.1451)	0.0301 (0.0048,0.0992)	4.95 (2.19,7.78)
Chad	1.3963 (0.2343,4.0901)	0.0132 (0.0022,0.0385)	8.05 (4.86,11.33)
Chile	1.7495 (0.4848,4.3548)	0.008 (0.0022,0.02)	11.05 (8.46,13.7)
China	96.2108 (14.9527,297.4445)	0.0056 (9e-04,0.0172)	4.45 (2.25,6.69)
Colombia	6.223 (1.1974,18.3568)	0.0116 (0.0022,0.0341)	11.38 (8.75,14.07)
Comoros	0.0153 (0.0021,0.0493)	0.0022 (3e-04,0.007)	16.41 (14,18.87)
Congo (Brazzaville)	0.8062 (0.1182,2.6993)	0.0169 (0.0025,0.0566)	4.9 (2.38,7.48)
Cook Islands	0 (0,2e-04)	2e-04 (0,9e-04)	9.72 (7.87,11.6)
Costa Rica	0.1222 (0.0185,0.4079)	0.0023 (3e-04,0.0077)	5.59 (2.84,8.41)
Côte d'Ivoire	2.5864 (0.4391,7.556)	0.0126 (0.0021,0.0369)	-0.37 (-2.61,1.93)
Croatia	0.0061 (0.0015,0.019)	1e-04 (0,4e-04)	1.97 (-0.83,4.85)
Cuba	0.0875 (0.016,0.2626)	6e-04 (1e-04,0.0019)	7.12 (6.04,8.21)
Cyprus	0.0077 (0.0014,0.0237)	5e-04 (1e-04,0.0014)	6.22 (2.38,10.21)
Czechia	0.0668 (0.0194,0.1655)	6e-04 (2e-04,0.0015)	7.54 (5.62,9.49)
Denmark	0.0258 (0.0063,0.0727)	4e-04 (1e-04,0.001)	5.91 (4.62,7.22)
Djibouti	0.295 (0.1047,0.7133)	0.0248 (0.0088,0.0601)	19.62 (13.56,26.02)
Dominica	0.0021 (3e-04,0.0076)	0.0029 (4e-04,0.0104)	-1.19 (-2.69,0.34)
Dominican Republic	0.4424 (0.0589,1.7114)	0.004 (5e-04,0.0153)	-0.77 (-3.2,1.72)
DR Congo	3.3316 (0.5647,10.3758)	0.0048 (8e-04,0.0149)	4.04 (0.84,7.33)
Ecuador	6.7652 (1.1914,23.6665)	0.0369 (0.0065,0.1292)	10.01 (6.18,13.98)
Egypt	0.2353 (0.0536,0.6847)	2e-04 (1e-04,7e-04)	11.31 (8.32,14.39)
El Salvador	0.4861 (0.0699,1.5582)	0.0078 (0.0011,0.0248)	3.75 (0.59,7.02)
Equatorial Guinea	0.1724 (0.0285,0.5465)	0.0146 (0.0024,0.046)	8.95 (5.79,12.21)
Eritrea	0.6534 (0.1228,1.9421)	0.0124 (0.0023,0.0369)	8.1 (4.53,11.8)
Estonia	1.24 (0.5335,2.352)	0.0922 (0.0397,0.1748)	8.29 (5.63,11.02)
Eswatini	4.9736 (1.1801,12.7877)	0.4912 (0.1181,1.2841)	17.91 (11.62,24.54)

Ethiopia	6.3548 (0.9827,22.121)	0.0083 (0.0013,0.0284)	5.2 (1.54,8.98)
Federated States of Micronesia	0.0018 (2e-04,0.0085)	0.0018 (2e-04,0.0086)	13.76 (11.73,15.82)
Fiji	0.0023 (2e-04,0.0104)	2e-04 (0,0.0011)	12.77 (10.74,14.84)
Finland	0.0645 (0.016,0.172)	0.0011 (3e-04,0.0028)	13.78 (12.14,15.44)
France	1.9595 (0.5513,5.2897)	0.0024 (7e-04,0.0066)	1.43 (-0.01,2.89)
Gabon	0.3438 (0.0596,1.1055)	0.0223 (0.0039,0.0717)	9.39 (6.32,12.54)
Georgia	3.017 (1.3735,5.2339)	0.0702 (0.032,0.1218)	19.04 (15.82,22.35)
Germany	1.7491 (0.546,3.9856)	0.0015 (5e-04,0.0035)	6.11 (5.19,7.03)
Ghana	4.2672 (0.6756,13.5505)	0.0153 (0.0024,0.0484)	8.47 (5.41,11.63)
Greece	0.1993 (0.0363,0.6012)	0.0017 (3e-04,0.0052)	11.13 (8.76,13.55)
Greenland	0.0132 (0.0018,0.0466)	0.0191 (0.0027,0.0672)	5.72 (4.85,6.59)
Grenada	6e-04 (1e-04,0.0025)	5e-04 (1e-04,0.0021)	0.72 (-0.83,2.3)
Guam	0.0011 (1e-04,0.0045)	7e-04 (1e-04,0.0027)	7.21 (4,10.51)
Guatemala	2.452 (0.4118,7.104)	0.0171 (0.0029,0.0495)	4.74 (1.47,8.12)
Guinea	1.1457 (0.2079,3.6509)	0.0127 (0.0023,0.0405)	10.17 (6.74,13.71)
Guinea-Bissau	0.5089 (0.0751,1.718)	0.0327 (0.0048,0.1101)	12.52 (9.42,15.71)
Guyana	0.0932 (0.0112,0.3951)	0.0123 (0.0015,0.0521)	4.16 (1.38,7.01)
Haiti	1.1963 (0.1631,4.1432)	0.0107 (0.0015,0.037)	-0.5 (-2.38,1.41)
Honduras	1.1587 (0.1969,3.5126)	0.0121 (0.002,0.0365)	8.04 (5.53,10.6)
Hungary	0.0442 (0.0087,0.1405)	4e-04 (1e-04,0.0012)	-2.4 (-5.13,0.4)
Iceland	8e-04 (1e-04,0.0027)	2e-04 (0,7e-04)	1.48 (-2.01,5.1)
India	150.4339 (34.0274,369.9189)	0.0104 (0.0023,0.0255)	13.89 (8.53,19.5)
Indonesia	4.7933 (0.6471,18.3403)	0.0016 (2e-04,0.006)	4.89 (2.24,7.6)
Iran	1.7827 (0.5489,4.2805)	0.0018 (6e-04,0.0043)	16.1 (13.25,19.02)
Iraq	0.0049 (0.0013,0.0125)	0 (0,0)	9.92 (6.2,13.78)
Ireland	0.0535 (0.0107,0.1616)	0.001 (2e-04,0.0029)	5.4 (3.77,7.06)
Israel	0.1402 (0.0364,0.3728)	0.0014 (4e-04,0.0038)	1.74 (0.34,3.17)
Italy	0.4361 (0.1263,1.1449)	5e-04 (2e-04,0.0014)	-0.31 (-2.51,1.94)
Jamaica	0.0898 (0.0136,0.3644)	0.0029 (4e-04,0.0119)	6.27 (4.43,8.15)
Japan	0.4432 (0.0528,1.7167)	3e-04 (0,0.001)	3.89 (2.13,5.67)
Jordan	0.0089 (0.0015,0.0317)	1e-04 (0,3e-04)	8.04 (4.72,11.46)
Kazakhstan	7.2223 (3.3566,12.4281)	0.0347 (0.0161,0.0597)	7.05 (2.94,11.32)
Kenya	8.2047 (1.8456,20.7057)	0.021 (0.0047,0.053)	6.83 (2.09,11.79)
Kiribati	0.0019 (2e-04,0.0079)	0.0016 (2e-04,0.0068)	4.89 (2.71,7.13)
Kuwait	0.0024 (5e-04,0.0073)	0 (0,1e-04)	6.34 (4.81,7.9)
Kyrgyzstan	7.813 (2.5697,15.9883)	0.1178 (0.0388,0.2411)	17.61 (13.04,22.36)
Laos	0.3466 (0.0465,1.4573)	0.0049 (7e-04,0.0204)	11.63 (7.35,16.08)
Latvia	1.4128 (0.5617,2.8036)	0.0719 (0.0286,0.1426)	4.22 (2.49,5.98)
Lebanon	0.0261 (0.0032,0.1044)	4e-04 (1e-04,0.0017)	8.01 (5.57,10.5)
Lesotho	6.1776 (1.7315,15.6996)	0.3848 (0.109,0.9834)	17.65 (12.73,22.79)
Liberia	0.0809 (0.0125,0.2589)	0.0019 (3e-04,0.0062)	3.48 (0.59,6.45)
Libya	0.1235 (0.014,0.4532)	0.0016 (2e-04,0.0058)	9.85 (6.96,12.83)
Lithuania	1.3699 (0.6303,2.3188)	0.0481 (0.0221,0.0813)	11.26 (8.5,14.08)
Luxembourg	0.0067 (9e-04,0.0241)	8e-04 (1e-04,0.003)	10.33 (8.18,12.53)

Madagascar	0.1963 (0.0326,0.6782)	9e-04 (1e-04,0.003)	22.45 (17.06,28.09)
Malawi	7.1342 (1.4178,20.4665)	0.0533 (0.0107,0.1524)	6.5 (2.07,11.13)
Malaysia	2.3806 (0.5429,6.9976)	0.0069 (0.0016,0.0203)	6.55 (2.92,10.31)
Maldives	0.0011 (1e-04,0.0044)	2e-04 (0,7e-04)	-1.39 (-4.76,2.09)
Mali	1.2454 (0.1905,3.6)	0.0075 (0.0012,0.0218)	8.16 (4.93,11.49)
Malta	0.0037 (4e-04,0.0146)	7e-04 (1e-04,0.0029)	6.3 (2.97,9.74)
Marshall Islands	0.0055 (7e-04,0.0216)	0.0098 (0.0013,0.0385)	18.42 (15.39,21.53)
Mauritania	0.0262 (0.0046,0.0786)	8e-04 (1e-04,0.0025)	4.94 (2.45,7.49)
Mauritius	0.008 (0.0015,0.0255)	5e-04 (1e-04,0.0017)	13.26 (9.08,17.6)
Mexico	10.8531 (1.9985,33.4019)	0.0079 (0.0014,0.0242)	8.63 (5.32,12.03)
Moldova	8.6282 (4.8257,13.1684)	0.1931 (0.108,0.2948)	14.97 (11.72,18.31)
Monaco	4e-04 (1e-04,0.0017)	8e-04 (1e-04,0.0031)	7.86 (6.5,9.24)
Mongolia	0.2478 (0.0627,0.6388)	0.0073 (0.0018,0.0188)	18.25 (15.9,20.64)
Montenegro	0.0017 (3e-04,0.0061)	3e-04 (0,9e-04)	6.37 (2.77,10.1)
Morocco	0.5915 (0.1499,1.5194)	0.0015 (4e-04,0.0039)	10.12 (5.97,14.42)
Mozambique	42.1492 (11.4673,102.3922)	0.1766 (0.0479,0.4284)	14.18 (10.47,18.01)
Myanmar	58.021 (18.2626,137.0867)	0.1007 (0.0317,0.238)	10.13 (6.68,13.7)
Namibia	2.8598 (0.9894,6.5564)	0.1357 (0.0467,0.309)	15.23 (10.07,20.64)
Nauru	2e-04 (0,9e-04)	0.0019 (2e-04,0.0088)	13.41 (11.29,15.57)
Nepal	1.6625 (0.3987,4.2964)	0.0058 (0.0014,0.0149)	20.29 (13.82,27.11)
Netherlands	0.271 (0.0794,0.6679)	0.0013 (4e-04,0.0032)	6.06 (5.06,7.07)
New Zealand	0.0211 (0.0043,0.0653)	4e-04 (1e-04,0.0011)	5.09 (3.92,6.28)
Nicaragua	0.2168 (0.0362,0.7652)	0.0033 (5e-04,0.0115)	8.67 (6.7,10.68)
Niger	0.4633 (0.0772,1.3536)	0.0033 (5e-04,0.0096)	5.92 (2.27,9.7)
Nigeria	23.4616 (6.0805,63.7935)	0.0142 (0.0037,0.0389)	9.76 (5.76,13.91)
Niue	0 (0,1e-04)	8e-04 (1e-04,0.0034)	11.67 (9.09,14.31)
North Korea	7.5964 (2.2672,18.1638)	0.025 (0.0075,0.0597)	17.09 (13.13,21.18)
North Macedonia	0.0073 (0.0019,0.0194)	3e-04 (1e-04,8e-04)	7.78 (5.21,10.42)
Northern Mariana Islands	0.0011 (1e-04,0.0045)	0.002 (3e-04,0.0081)	6.96 (3.14,10.92)
Norway	0.056 (0.0142,0.1518)	9e-04 (2e-04,0.0025)	2.95 (1.54,4.37)
Oman	0.0073 (0.0013,0.0231)	1e-04 (0,4e-04)	3.06 (1.33,4.82)
Pakistan	6.6983 (1.8988,17.3239)	0.003 (9e-04,0.0079)	35.83 (31.98,39.8)
Palau	2e-04 (0,9e-04)	0.001 (1e-04,0.0043)	12.77 (10.47,15.12)
Palestine	0.0026 (3e-04,0.0094)	1e-04 (0,2e-04)	6.86 (3.86,9.95)
Panama	0.6788 (0.1085,2.0885)	0.0155 (0.0025,0.0477)	5.64 (2.66,8.7)
Papua New Guinea	6.9417 (2.0092,16.8749)	0.0795 (0.023,0.1931)	27.19 (22.31,32.27)
Paraguay	1.6956 (0.2375,5.394)	0.0238 (0.0033,0.0756)	10.69 (8.02,13.42)
Peru	13.0742 (5.2859,27.9509)	0.0346 (0.014,0.074)	7.22 (3.61,10.96)
Philippines	2.3162 (0.61,6.4311)	0.002 (5e-04,0.0056)	24.41 (18.77,30.32)
Poland	0.1893 (0.0501,0.5313)	4e-04 (1e-04,0.0012)	6.01 (2.08,10.08)
Portugal	0.7672 (0.1382,2.3566)	0.0057 (0.001,0.0175)	-0.57 (-2.67,1.58)
Puerto Rico	0.0409 (0.0078,0.1274)	0.001 (2e-04,0.0031)	-0.44 (-3.01,2.19)
Qatar	0.002 (4e-04,0.0062)	1e-04 (0,2e-04)	5.7 (4.58,6.84)
Romania	1.6703 (0.5216,3.9356)	0.009 (0.0028,0.0213)	5.83 (3.91,7.79)

Russia	468.1711 (245.3128,760.5384)	0.2926 (0.1534,0.4747)	12.77 (9.35,16.31)
Rwanda	2.1799 (0.7098,5.014)	0.0214 (0.007,0.0492)	7.28 (2.66,12.11)
Saint Kitts and Nevis	0.0012 (2e-04,0.0046)	0.0017 (2e-04,0.0063)	1 (-0.76,2.79)
Saint Lucia	0.0024 (4e-04,0.0087)	0.0011 (2e-04,0.0041)	-3.77 (-5.63,-1.87)
Saint Vincent and the Grenadines	0.0027 (4e-04,0.0095)	0.0021 (3e-04,0.0076)	-3.16 (-4.72,-1.57)
Samoa	5e-04 (1e-04,0.0018)	2e-04 (0,9e-04)	7.89 (4.52,11.36)
San Marino	2e-04 (0,8e-04)	5e-04 (1e-04,0.0017)	8.89 (7.28,10.53)
São Tomé and Príncipe	0.0024 (4e-04,0.0075)	0.0014 (2e-04,0.0043)	5.2 (2.75,7.7)
Saudi Arabia	0.4678 (0.0927,1.5083)	0.001 (2e-04,0.0033)	8.21 (4.27,12.29)
Senegal	0.291 (0.0733,0.7324)	0.0024 (6e-04,0.006)	7.68 (3.73,11.78)
Serbia	0.1957 (0.0455,0.5249)	0.0021 (5e-04,0.0056)	7.19 (5.06,9.35)
Seychelles	4e-04 (0,0.0017)	4e-04 (0,0.0014)	1.41 (-2.21,5.17)
Sierra Leone	0.4471 (0.0769,1.418)	0.0068 (0.0012,0.0216)	10.75 (6.89,14.74)
Singapore	0.097 (0.0307,0.244)	0.0012 (4e-04,0.0031)	8.48 (6.67,10.32)
Slovakia	0.0016 (3e-04,0.0048)	0 (0,1e-04)	2.65 (0.23,5.12)
Slovenia	3e-04 (0,9e-04)	0 (0,0)	-7.97 (-10.74,-5.11)
Solomon Islands	0.0114 (0.001,0.0475)	0.0018 (2e-04,0.0073)	14.24 (11.68,16.86)
Somalia	3.0184 (0.7384,8.3864)	0.023 (0.0056,0.0638)	18.95 (13.16,25.03)
South Africa	62.9055 (17.4894,158.9383)	0.1038 (0.0289,0.2621)	11.23 (6.08,16.63)
South Korea	0.9332 (0.1333,3.5046)	0.0013 (2e-04,0.005)	4.66 (1.95,7.45)
South Sudan	2.0404 (0.3764,6.1021)	0.0297 (0.0055,0.0889)	15.1 (11.21,19.14)
Spain	0.6672 (0.0983,2.7183)	0.0011 (2e-04,0.0044)	-2.7 (-4.09,-1.28)
Sri Lanka	0.0248 (0.0027,0.0958)	1e-04 (0,4e-04)	1.93 (-0.74,4.66)
Sudan	1.3416 (0.1744,5.204)	0.0045 (6e-04,0.0174)	7.09 (3.62,10.68)
Suriname	0.0157 (0.0023,0.062)	0.0026 (4e-04,0.0101)	0.77 (-0.95,2.53)
Sweden	0.1138 (0.0336,0.2857)	0.001 (3e-04,0.0025)	6.02 (4.29,7.77)
Switzerland	0.1193 (0.0381,0.3041)	0.001 (3e-04,0.0027)	3.63 (1.84,5.46)
Syria	0.0081 (0.0011,0.0295)	1e-04 (0,2e-04)	6.41 (3.59,9.31)
Taiwan (province of China)	0.3385 (0.0489,1.2205)	0.0011 (2e-04,0.0041)	3.37 (1.86,4.91)
Tajikistan	3.4243 (1.312,6.4725)	0.036 (0.0138,0.068)	13.24 (8.74,17.93)
Tanzania	8.7489 (1.5207,29.6773)	0.0201 (0.0035,0.0683)	5.95 (2.41,9.6)
Thailand	57.3112 (13.5741,152.9636)	0.0679 (0.0161,0.1804)	12.2 (10.39,14.03)
The Bahamas	0.0331 (0.0043,0.1273)	0.0075 (0.001,0.0288)	9.33 (7.14,11.56)
The Gambia	0.1896 (0.0304,0.5726)	0.0111 (0.0018,0.0334)	11.71 (8.09,15.45)
Timor-Leste	0.0514 (0.0067,0.1982)	0.0045 (6e-04,0.0175)	2.22 (-1.14,5.69)
Togo	0.6636 (0.0959,2.1625)	0.0099 (0.0014,0.0322)	7.54 (3.95,11.27)
Tokelau	0 (0,0)	6e-04 (1e-04,0.0024)	10.57 (7.92,13.28)
Tonga	2e-04 (0,0.0012)	3e-04 (0,0.0013)	6.67 (4.1,9.29)
Trinidad and Tobago	0.0294 (0.0037,0.1085)	0.0018 (2e-04,0.0066)	-0.29 (-2.37,1.84)
Tunisia	0.0559 (0.0153,0.1519)	4e-04 (1e-04,0.0011)	13.09 (10.15,16.12)
Türkiye	0.1721 (0.0596,0.3665)	2e-04 (1e-04,4e-04)	11.68 (8.17,15.31)
Turkmenistan	4.2343 (1.3308,8.8494)	0.0798 (0.0251,0.1667)	13.64 (9.01,18.46)
Tuvalu	2e-04 (0,0.001)	0.0019 (2e-04,0.0085)	11.08 (9.4,12.79)
Uganda	14.846 (3.7317,38.3137)	0.0515 (0.0129,0.1331)	7.59 (3.69,11.63)

UK	0.5805 (0.1446,1.6031)	7e-04 (2e-04,0.0021)	5.23 (4.23,6.23)
Ukraine	233.8565 (105.0801,410.4389)	0.461 (0.2071,0.809)	13.86 (10.53,17.29)
United Arab Emirates	0.005 (7e-04,0.0204)	0 (0,1e-04)	6.49 (3.66,9.41)
Uruguay	0.0765 (0.014,0.2511)	0.0021 (4e-04,0.0069)	9.5 (7.39,11.66)
USA	1.9035 (0.6108,5.1445)	5e-04 (1e-04,0.0012)	-0.92 (-1.58,-0.25)
Uzbekistan	22.4052 (7.509,45.7506)	0.0605 (0.0203,0.1236)	17.73 (12.9,22.76)
Vanuatu	0.0037 (4e-04,0.0141)	0.0012 (1e-04,0.0048)	20.19 (17.8,22.64)
Venezuela	2.965 (0.4756,10.1927)	0.0101 (0.0016,0.0347)	7.59 (5.28,9.96)
Viet Nam	45.0427 (11.6601,114.4082)	0.0404 (0.0105,0.1026)	14.68 (10.96,18.53)
Virgin Islands	8e-04 (1e-04,0.0031)	9e-04 (1e-04,0.0035)	2.79 (1.09,4.52)
Yemen	0.4586 (0.0881,1.606)	0.0016 (3e-04,0.0057)	12 (8.43,15.68)
Zambia	10.1577 (1.6911,32.4507)	0.0726 (0.012,0.2321)	4.5 (1.37,7.72)
Zimbabwe	16.9362 (2.7134,53.9846)	0.1445 (0.023,0.458)	8.96 (4.96,13.1)

Abbreviations: MDR-TB= multidrug-resistant tuberculosis. XDR-TB=extensively drug-resistant tuberculosis. ASR=age-standardized rate. EAPC=estimated annual percentage change.

Table S13 The death of cases and ASR of HIV/AIDS-XDR-TB in 2021, and the trends from baseline 2021 by countries.

Location	Deaths_Number_2021	Deaths_ASR_2021	EAPC
Afghanistan	0.1084 (0.0131,0.3904)	5e-04 (1e-04,0.0017)	10.73 (6.53,15.09)
Albania	0.0025 (3e-04,0.0087)	1e-04 (0,4e-04)	6.04 (3,9.17)
Algeria	0.2412 (0.0234,0.9704)	5e-04 (1e-04,0.0021)	11.76 (9.71,13.85)
American Samoa	0 (0,1e-04)	1e-04 (0,3e-04)	6.14 (4.09,8.22)
Andorra	0 (0,1e-04)	0 (0,1e-04)	6.66 (4.89,8.47)
Angola	3.3946 (0.4394,11.2788)	0.0142 (0.0018,0.0473)	16.53 (12.13,21.11)
Antigua and Barbuda	3e-04 (0,0.0013)	3e-04 (0,0.0012)	-2.67 (-4.33,-0.97)
Argentina	5.2461 (0.5979,20.8893)	0.0105 (0.0012,0.0419)	6.3 (2.98,9.72)
Armenia	0.459 (0.1073,1.0872)	0.0131 (0.0031,0.0311)	19.82 (15.45,24.34)
Australia	0.1021 (0.0228,0.2839)	3e-04 (1e-04,9e-04)	7.92 (7.14,8.7)
Austria	0.1008 (0.0205,0.2776)	0.001 (2e-04,0.0026)	8.31 (6.32,10.33)
Azerbaijan	1.769 (0.4973,3.8471)	0.0143 (0.004,0.031)	15.67 (10.92,20.62)
Bahrain	0.0045 (5e-04,0.0171)	2e-04 (0,9e-04)	3.82 (0.41,7.36)
Bangladesh	0.9595 (0.2096,2.7079)	6e-04 (1e-04,0.0016)	36.72 (29.78,44.02)
Barbados	9e-04 (1e-04,0.0042)	2e-04 (0,0.0012)	-7.19 (-9.48,-4.83)

Belarus	4.0611 (2.0727,6.9036)	0.0377 (0.0193,0.0641)	12.04 (7.75,16.5)
Belgium	0.1132 (0.0205,0.3411)	9e-04 (2e-04,0.0026)	2.67 (1.22,4.15)
Belize	0.0127 (0.001,0.0508)	0.003 (2e-04,0.0121)	0.12 (-2.1,2.4)
Benin	0.3148 (0.0442,1.0901)	0.0035 (5e-04,0.012)	10.14 (5.08,15.44)
Bermuda	0 (0,2e-04)	0 (0,2e-04)	-4.06 (-6.17,-1.9)
Bhutan	0.0064 (8e-04,0.0236)	8e-04 (1e-04,0.0029)	7.15 (4.13,10.26)
Bolivia	1.2865 (0.1802,4.5878)	0.0111 (0.0016,0.0394)	9.8 (4.96,14.87)
Bosnia and Herzegovina	0.0063 (0.0011,0.0236)	2e-04 (0,7e-04)	8.33 (6.11,10.6)
Botswana	2.9503 (0.4737,9.5673)	0.1289 (0.0206,0.419)	14.26 (9.96,18.72)
Brazil	30.7805 (4.5009,107.4563)	0.0122 (0.0018,0.0427)	11.26 (7.59,15.07)
Brunei	7e-04 (1e-04,0.0028)	2e-04 (0,6e-04)	6.06 (4.21,7.94)
Bulgaria	0.0119 (0.0019,0.0393)	2e-04 (0,6e-04)	7.55 (4.04,11.17)
Burkina Faso	1.0698 (0.1519,3.2264)	0.0072 (0.001,0.0217)	2.66 (0.13,5.26)
Burundi	0.9054 (0.1217,3.07)	0.0101 (0.0013,0.0358)	3.28 (-0.65,7.37)
Cabo Verde	0.0095 (0.0012,0.0334)	0.0017 (2e-04,0.006)	4.29 (1.31,7.36)
Cambodia	1.485 (0.1672,5.6962)	0.0091 (0.001,0.0353)	9.42 (0.56,19.06)
Cameroon	2.732 (0.3457,9.3014)	0.0121 (0.0015,0.0409)	8.39 (4.42,12.5)
Canada	0.2346 (0.0495,0.7012)	5e-04 (1e-04,0.0015)	1.27 (0.2,2.36)
Central African Republic	0.9977 (0.1302,3.5042)	0.0239 (0.0031,0.0885)	4.98 (2.18,7.86)
Chad	1.1116 (0.1724,3.4107)	0.0105 (0.0016,0.0317)	8.67 (5.1,12.36)
Chile	1.396 (0.2945,4.1661)	0.0064 (0.0014,0.0192)	11.47 (8.62,14.39)
China	57.0109 (7.5021,199.9166)	0.0032 (4e-04,0.0112)	5.65 (2.58,8.8)
Colombia	4.8755 (0.7703,14.516)	0.009 (0.0014,0.0269)	11.48 (8.71,14.32)
Comoros	0.0013 (1e-04,0.0042)	2e-04 (0,6e-04)	17.83 (13.17,22.69)
Congo (Brazzaville)	0.641 (0.068,2.5657)	0.0134 (0.0014,0.0538)	4.93 (2.39,7.53)
Cook Islands	0 (0,2e-04)	2e-04 (0,9e-04)	9.78 (7.92,11.68)
Costa Rica	0.0947 (0.0114,0.3418)	0.0018 (2e-04,0.0064)	5.7 (2.78,8.7)
Côte d'Ivoire	2.0653 (0.2829,6.9666)	0.0101 (0.0014,0.0339)	-0.29 (-2.6,2.07)
Croatia	0.0047 (7e-04,0.0162)	1e-04 (0,4e-04)	2.04 (-0.84,5.01)
Cuba	0.0519 (0.0074,0.1711)	4e-04 (1e-04,0.0013)	8.32 (7.19,9.47)
Cyprus	0.0055 (8e-04,0.0185)	3e-04 (0,0.0011)	5.98 (2.09,10.02)
Czechia	0.0422 (0.0088,0.1157)	4e-04 (1e-04,0.001)	8.64 (6.36,10.97)
Denmark	0.0191 (0.0032,0.0604)	3e-04 (0,9e-04)	5.99 (4.52,7.47)
Djibouti	0.234 (0.0562,0.6108)	0.0197 (0.0047,0.0515)	21.79 (14.28,29.79)
Dominica	0.0017 (2e-04,0.0065)	0.0023 (2e-04,0.0089)	-1.16 (-2.72,0.42)
Dominican Republic	0.3406 (0.0314,1.5006)	0.0031 (3e-04,0.0135)	-0.59 (-3.14,2.02)
DR Congo	2.367 (0.3316,8.652)	0.0034 (5e-04,0.0125)	4.07 (0.74,7.5)
Ecuador	4.0401 (0.5414,13.8061)	0.0225 (0.003,0.0768)	12.44 (7.52,17.59)
Egypt	0.1489 (0.0247,0.491)	2e-04 (0,5e-04)	12.71 (8.92,16.62)
El Salvador	0.3479 (0.0429,1.2317)	0.0056 (7e-04,0.0197)	3.96 (0.45,7.59)
Equatorial Guinea	0.1296 (0.0154,0.4388)	0.0112 (0.0013,0.0377)	10 (6.11,14.02)
Eritrea	0.5204 (0.0733,1.706)	0.0099 (0.0014,0.0333)	8.7 (4.7,12.86)
Estonia	0.6323 (0.251,1.256)	0.0451 (0.0179,0.0897)	11.88 (8.01,15.88)
Eswatini	3.9328 (0.8171,10.5283)	0.3882 (0.0799,1.005)	20.51 (12.83,28.72)

Ethiopia	4.8355 (0.6419,15.9514)	0.0063 (8e-04,0.0205)	6.05 (1.76,10.52)
Federated States of Micronesia	0.0014 (1e-04,0.0064)	0.0014 (1e-04,0.0064)	14.94 (12.29,17.65)
Fiji	0.0018 (1e-04,0.0085)	2e-04 (0,9e-04)	13.03 (10.78,15.32)
Finland	0.0491 (0.0092,0.14)	8e-04 (2e-04,0.0023)	13.73 (12.08,15.41)
France	1.5511 (0.3054,4.866)	0.0019 (4e-04,0.0061)	1.43 (-0.02,2.9)
Gabon	0.2745 (0.0343,1.0081)	0.0178 (0.0022,0.0655)	9.75 (6.46,13.15)
Georgia	1.5586 (0.6039,2.8704)	0.0393 (0.0152,0.0724)	21.34 (17.99,24.78)
Germany	1.3561 (0.3293,3.4551)	0.0012 (3e-04,0.0031)	6.01 (5.07,6.95)
Ghana	3.3986 (0.3757,12.2843)	0.0121 (0.0013,0.0439)	8.78 (5.52,12.14)
Greece	0.1575 (0.026,0.5026)	0.0014 (2e-04,0.0043)	11.2 (8.79,13.67)
Greenland	0.0055 (6e-04,0.0198)	0.0078 (9e-04,0.0283)	2.93 (1.59,4.29)
Grenada	3e-04 (0,0.0012)	2e-04 (0,0.0011)	-0.42 (-2.13,1.32)
Guam	8e-04 (1e-04,0.0033)	5e-04 (0,0.002)	6.86 (3.7,10.12)
Guatemala	1.9487 (0.2619,6.7643)	0.0136 (0.0018,0.0471)	5.13 (1.58,8.8)
Guinea	0.9136 (0.1159,3.3516)	0.0102 (0.0013,0.0369)	10.82 (7.01,14.77)
Guinea-Bissau	0.4044 (0.0424,1.5699)	0.026 (0.0028,0.1005)	15.07 (10.81,19.49)
Guyana	0.0738 (0.0069,0.3143)	0.0097 (9e-04,0.0413)	4.15 (1.37,7.01)
Haiti	0.9484 (0.0854,4.8188)	0.0085 (8e-04,0.0428)	-0.51 (-2.38,1.4)
Honduras	0.922 (0.1328,2.9455)	0.0096 (0.0014,0.0306)	8.75 (5.84,11.74)
Hungary	0.0254 (0.0035,0.0902)	2e-04 (0,9e-04)	-3.1 (-5.93,-0.18)
Iceland	5e-04 (1e-04,0.002)	1e-04 (0,5e-04)	1.07 (-2.51,4.77)
India	82.0883 (15.6483,220.5903)	0.0057 (0.0011,0.0152)	16.4 (9.13,24.16)
Indonesia	3.0305 (0.3504,12.3017)	0.001 (1e-04,0.004)	8.11 (4.49,11.85)
Iran	0.8535 (0.2038,2.23)	9e-04 (2e-04,0.0022)	21.5 (17.18,25.97)
Iraq	0.0032 (6e-04,0.0092)	0 (0,0)	9.25 (5.44,13.19)
Ireland	0.04 (0.0063,0.1303)	7e-04 (1e-04,0.0024)	6.04 (4.16,7.95)
Israel	0.1071 (0.0214,0.3013)	0.0011 (2e-04,0.0032)	1.71 (0.24,3.21)
Italy	0.2849 (0.0573,0.8655)	4e-04 (1e-04,0.0011)	-0.65 (-2.81,1.56)
Jamaica	0.071 (0.0078,0.3048)	0.0023 (3e-04,0.01)	6.41 (4.46,8.4)
Japan	0.3334 (0.0307,1.3462)	2e-04 (0,8e-04)	5.17 (2.57,7.83)
Jordan	0.0051 (7e-04,0.0188)	0 (0,2e-04)	6.48 (2.99,10.09)
Kazakhstan	2.9797 (1.187,5.8249)	0.0144 (0.0057,0.0281)	5.12 (0.35,10.12)
Kenya	6.4697 (1.1181,18.4243)	0.0166 (0.0029,0.0465)	7.29 (2.24,12.59)
Kiribati	0.0015 (1e-04,0.0064)	0.0013 (1e-04,0.0055)	5.04 (2.81,7.32)
Kuwait	0.0013 (2e-04,0.0043)	0 (0,1e-04)	6.25 (3.38,9.19)
Kyrgyzstan	3.4263 (1.123,6.7372)	0.0504 (0.0165,0.0991)	15.97 (10.57,21.64)
Laos	0.2689 (0.0244,1.168)	0.0038 (3e-04,0.016)	21.75 (14.03,29.99)
Latvia	0.848 (0.2646,1.8413)	0.0427 (0.0133,0.0928)	4.14 (2.04,6.29)
Lebanon	0.0163 (0.0015,0.0638)	3e-04 (0,0.001)	7.57 (4.77,10.44)
Lesotho	4.9242 (1.1077,13.4025)	0.3065 (0.0682,0.85)	19.12 (13.36,25.18)
Liberia	0.0637 (0.0078,0.2429)	0.0015 (2e-04,0.0058)	3.86 (0.65,7.17)
Libya	0.0338 (9e-04,0.2046)	4e-04 (0,0.0026)	10.16 (6.29,14.18)
Lithuania	0.5548 (0.2169,1.0696)	0.0201 (0.0078,0.0387)	9.43 (6.27,12.68)
Luxembourg	0.0023 (3e-04,0.0092)	3e-04 (0,0.0012)	6.95 (4.78,9.17)

Madagascar	0.1565 (0.0169,0.5531)	7e-04 (1e-04,0.0025)	24.18 (17.59,31.14)
Malawi	5.6894 (0.9163,17.9463)	0.0425 (0.0071,0.1298)	7.16 (2.29,12.26)
Malaysia	1.8139 (0.2873,6.0456)	0.0053 (8e-04,0.0176)	7.68 (3.2,12.36)
Maldives	8e-04 (1e-04,0.0034)	1e-04 (0,5e-04)	-0.57 (-4.58,3.62)
Mali	0.9924 (0.1155,3.3403)	0.006 (7e-04,0.0203)	8.65 (5.1,12.31)
Malta	4e-04 (0,0.0016)	1e-04 (0,3e-04)	2.85 (-0.66,6.48)
Marshall Islands	0.004 (4e-04,0.0159)	0.0071 (7e-04,0.0282)	21.58 (17.42,25.89)
Mauritania	0.001 (1e-04,0.0038)	0 (0,1e-04)	6.51 (3.01,10.13)
Mauritius	0.0056 (7e-04,0.0204)	4e-04 (0,0.0013)	14.01 (9.54,18.66)
Mexico	8.1209 (1.2191,27.8078)	0.0059 (9e-04,0.0201)	8.67 (5.23,12.21)
Moldova	2.0767 (1.0681,3.5482)	0.0481 (0.0247,0.0821)	11.31 (6.97,15.83)
Monaco	4e-04 (0,0.0015)	7e-04 (1e-04,0.0028)	7.93 (6.52,9.35)
Mongolia	0.1642 (0.032,0.447)	0.0045 (9e-04,0.0123)	18.07 (15.38,20.82)
Montenegro	0.0012 (1e-04,0.0049)	2e-04 (0,8e-04)	6.5 (2.8,10.34)
Morocco	0.4674 (0.0852,1.3853)	0.0012 (2e-04,0.0035)	11.53 (6.66,16.62)
Mozambique	32.1559 (7.3995,82.5448)	0.1318 (0.0305,0.3401)	16.08 (11.31,21.07)
Myanmar	34.875 (8.2392,92.1576)	0.0601 (0.0143,0.1591)	14.8 (9.28,20.59)
Namibia	2.2484 (0.6368,5.5453)	0.1067 (0.0298,0.2678)	16.7 (10.7,23.02)
Nauru	2e-04 (0,8e-04)	0.0015 (1e-04,0.008)	15.54 (12.63,18.52)
Nepal	1.2941 (0.2474,3.7342)	0.0045 (9e-04,0.0129)	27.33 (17.41,38.09)
Netherlands	0.209 (0.0431,0.5765)	0.001 (2e-04,0.0028)	6.01 (5,7.03)
New Zealand	0.0031 (5e-04,0.0102)	1e-04 (0,2e-04)	0.35 (-2.11,2.87)
Nicaragua	0.1688 (0.0219,0.6198)	0.0025 (3e-04,0.0093)	9.13 (6.98,11.33)
Niger	0.3629 (0.041,1.2236)	0.0026 (3e-04,0.0087)	6.56 (2.51,10.78)
Nigeria	17.5657 (3.2321,47.9892)	0.0108 (0.002,0.0297)	10.77 (6.14,15.6)
Niue	0 (0,0)	6e-04 (1e-04,0.003)	11.75 (9.08,14.49)
North Korea	3.494 (0.4699,12.136)	0.0118 (0.0017,0.0411)	25.35 (18.66,32.41)
North Macedonia	0.0047 (8e-04,0.0133)	2e-04 (0,6e-04)	8.98 (5.92,12.13)
Northern Mariana Islands	7e-04 (1e-04,0.0028)	0.0013 (1e-04,0.005)	6.68 (2.86,10.63)
Norway	0.0426 (0.0083,0.1381)	7e-04 (1e-04,0.0022)	2.7 (1.23,4.18)
Oman	0.0028 (4e-04,0.0101)	1e-04 (0,2e-04)	1.73 (-0.43,3.94)
Pakistan	5.1058 (0.3274,18.2003)	0.0023 (2e-04,0.0082)	50.9 (43.59,58.58)
Palau	2e-04 (0,8e-04)	8e-04 (1e-04,0.0039)	12.89 (10.52,15.3)
Palestine	0.0015 (1e-04,0.0058)	0 (0,1e-04)	6.36 (2.98,9.86)
Panama	0.5082 (0.0643,1.6493)	0.0117 (0.0015,0.0378)	5.56 (2.5,8.72)
Papua New Guinea	5.5363 (1.1502,14.2274)	0.0633 (0.0135,0.1638)	32.29 (24.55,40.51)
Paraguay	1.326 (0.1604,4.3384)	0.0186 (0.0023,0.061)	12.19 (8.89,15.59)
Peru	9.7029 (2.9394,24.1896)	0.0258 (0.0078,0.0642)	8.45 (4.15,12.91)
Philippines	1.3188 (0.2857,3.8924)	0.0012 (3e-04,0.0034)	25.36 (18.46,32.67)
Poland	0.1292 (0.0258,0.3675)	3e-04 (1e-04,8e-04)	7.9 (3.1,12.93)
Portugal	0.6088 (0.0827,1.908)	0.0045 (6e-04,0.0142)	-0.41 (-2.63,1.86)
Puerto Rico	0.0325 (0.0044,0.1226)	8e-04 (1e-04,0.003)	-0.43 (-3.01,2.22)
Qatar	4e-04 (1e-04,0.0015)	0 (0,0)	0.19 (-2.1,2.53)
Romania	1.1756 (0.2722,3.1852)	0.0064 (0.0015,0.0174)	6.11 (3.97,8.28)

Russia	141.5999 (65.2188,250.0444)	0.085 (0.0391,0.1501)	11.31 (7.37,15.4)
Rwanda	1.7365 (0.4054,4.5165)	0.0171 (0.004,0.045)	8.2 (2.98,13.69)
Saint Kitts and Nevis	0.001 (1e-04,0.0041)	0.0014 (1e-04,0.0056)	0.84 (-0.95,2.66)
Saint Lucia	0.0019 (2e-04,0.0074)	9e-04 (1e-04,0.0035)	-3.78 (-5.66,-1.87)
Saint Vincent and the Grenadines	0.0021 (3e-04,0.0086)	0.0017 (2e-04,0.0068)	-3.12 (-4.75,-1.45)
Samoa	4e-04 (0,0.0017)	2e-04 (0,9e-04)	8.22 (4.69,11.86)
San Marino	2e-04 (0,7e-04)	4e-04 (0,0.0014)	8.99 (7.33,10.68)
São Tomé and Príncipe	1e-04 (0,3e-04)	1e-04 (0,2e-04)	4.74 (1.44,8.15)
Saudi Arabia	0.328 (0.0458,1.1493)	7e-04 (1e-04,0.0024)	8.2 (3.89,12.69)
Senegal	0.2323 (0.0433,0.6818)	0.0019 (4e-04,0.0056)	8.44 (4,13.07)
Serbia	0.1206 (0.0207,0.3415)	0.0013 (2e-04,0.0036)	8.49 (5.85,11.19)
Seychelles	3e-04 (0,0.0015)	3e-04 (0,0.0012)	1.34 (-2.42,5.24)
Sierra Leone	0.3567 (0.0472,1.1877)	0.0054 (7e-04,0.0181)	11.56 (7.24,16.05)
Singapore	0.036 (0.0079,0.1084)	5e-04 (1e-04,0.0014)	5.44 (3.06,7.88)
Slovakia	0.0012 (1e-04,0.0043)	0 (0,1e-04)	2.51 (0.08,5.01)
Slovenia	2e-04 (0,6e-04)	0 (0,0)	-8.1 (-11,-5.09)
Solomon Islands	0.009 (7e-04,0.0397)	0.0014 (1e-04,0.0061)	14.65 (11.84,17.53)
Somalia	2.4039 (0.4566,6.9832)	0.0183 (0.0032,0.0544)	21.21 (14,28.87)
South Africa	40.6861 (8.6255,106.41)	0.0671 (0.0142,0.1752)	12.67 (6.51,19.2)
South Korea	0.3831 (0.0463,1.4681)	5e-04 (1e-04,0.0019)	6.61 (3.3,10.02)
South Sudan	1.6283 (0.252,5.6745)	0.0237 (0.0037,0.0813)	15.85 (11.42,20.45)
Spain	0.4927 (0.0534,1.9849)	8e-04 (1e-04,0.0033)	-2.71 (-4.22,-1.19)
Sri Lanka	0.0168 (0.0016,0.0708)	1e-04 (0,3e-04)	1.54 (-1.24,4.41)
Sudan	0.8925 (0.0961,3.6049)	0.0032 (3e-04,0.0129)	7.09 (3.34,10.98)
Suriname	0.0124 (0.0012,0.0539)	0.002 (2e-04,0.0088)	0.97 (-0.86,2.83)
Sweden	0.0751 (0.0167,0.2153)	6e-04 (1e-04,0.0018)	5.33 (3.48,7.2)
Switzerland	0.0711 (0.0159,0.1946)	7e-04 (1e-04,0.0018)	3.09 (0.89,5.35)
Syria	0.0047 (4e-04,0.0189)	0 (0,1e-04)	5.76 (2.58,9.05)
Taiwan (province of China)	0.2366 (0.0299,0.8983)	8e-04 (1e-04,0.0029)	3.95 (1.45,6.52)
Tajikistan	2.3785 (0.7452,5.0314)	0.0242 (0.0076,0.0512)	13.56 (8.48,18.88)
Tanzania	6.9783 (0.9659,25.5273)	0.016 (0.0022,0.0587)	6.45 (2.56,10.5)
Thailand	42.939 (7.6355,127.3413)	0.05 (0.0089,0.1483)	14.57 (11.99,17.22)
The Bahamas	0.026 (0.0027,0.1066)	0.0059 (6e-04,0.0241)	9.36 (7.15,11.62)
The Gambia	0.1508 (0.0192,0.5717)	0.0088 (0.0011,0.0335)	12.77 (8.54,17.16)
Timor-Leste	0.0409 (0.0036,0.174)	0.0036 (3e-04,0.0154)	2.22 (-1.15,5.71)
Togo	0.5283 (0.0693,1.9003)	0.0079 (0.001,0.0281)	7.78 (4.03,11.66)
Tokelau	0 (0,0)	5e-04 (0,0.0021)	10.88 (8.07,13.76)
Tonga	2e-04 (0,8e-04)	2e-04 (0,9e-04)	6.83 (4.14,9.59)
Trinidad and Tobago	0.0232 (0.0024,0.0996)	0.0014 (1e-04,0.0061)	-0.32 (-2.45,1.85)
Tunisia	0.0411 (0.0078,0.1322)	3e-04 (1e-04,0.001)	18.23 (13.6,23.06)
Türkiye	0.107 (0.0264,0.2807)	1e-04 (0,3e-04)	12.11 (7.83,16.56)
Turkmenistan	3.3024 (0.9568,6.8084)	0.0621 (0.018,0.1281)	14.22 (9.18,19.49)
Tuvalu	2e-04 (0,8e-04)	0.0015 (1e-04,0.0066)	15.55 (12.68,18.49)
Uganda	11.8093 (2.3065,36.0808)	0.0409 (0.008,0.125)	7.77 (3.74,11.95)

UK	0.3419 (0.0642,1.0224)	4e-04 (1e-04,0.0014)	4.68 (3.63,5.73)
Ukraine	109.6416 (48.9258,194.8981)	0.2133 (0.0951,0.3788)	13.04 (9.05,17.18)
United Arab Emirates	0.0035 (3e-04,0.0151)	0 (0,1e-04)	6.25 (3.07,9.53)
Uruguay	0.0466 (0.006,0.1666)	0.0012 (2e-04,0.0044)	8.58 (6.26,10.96)
USA	1.4569 (0.336,4.4318)	3e-04 (1e-04,0.001)	-1.02 (-1.7,-0.34)
Uzbekistan	9.5737 (3.2715,19.2795)	0.0261 (0.0089,0.0527)	16.45 (10.86,22.31)
Vanuatu	0.0029 (2e-04,0.0134)	0.001 (1e-04,0.0045)	20.46 (17.9,23.08)
Venezuela	2.3547 (0.2828,8.592)	0.008 (0.001,0.0293)	7.72 (5.31,10.19)
Viet Nam	34.5301 (6.1491,104.2928)	0.0307 (0.0055,0.0923)	16.77 (12.24,21.48)
Virgin Islands	6e-04 (1e-04,0.0027)	7e-04 (1e-04,0.003)	2.9 (1.12,4.71)
Yemen	0.3642 (0.0498,1.3197)	0.0013 (2e-04,0.0047)	12.85 (8.78,17.08)
Zambia	6.7272 (0.8419,21.2933)	0.0488 (0.0061,0.1527)	4.38 (1.05,7.83)
Zimbabwe	13.1267 (1.9019,43.9267)	0.1129 (0.0164,0.3722)	9.02 (4.95,13.26)

Abbreviations: MDR-TB=multidrug-resistant tuberculosis. XDR-TB=extensively drug-resistant tuberculosis. ASR=age-standardized rate. EAPC=estimated annual percentage change.

Table S14 The global incidence of cases and ASR of drug-resistant tuberculosis in 2021, and the trends from baseline to 2021 by sex and age group.

Age		Number_2021	Fold change	Incidence rate_2021	EAPC
		**MDR-TB**			
<5 years					
	Both	13989.75 (8912.07,20803.98)	2.42	2.1255 (1.3541,3.1609)	0.94 (-0.35,2.25)
	Female	8195.28 (5294.2,12228.99)	2.85	2.5756 (1.6638,3.8433)	1.28 (-0.06,2.64)
	Male	5794.47 (3693.35,8634.1)	1.95	1.7044 (1.0863,2.5396)	0.51 (-0.71,1.76)
5-14 years					
	Both	18525.09 (10032.34,33091.71)	3.64	1.3685 (0.7411,2.4446)	1.4 (0.09,2.72)
	Female	10773.12 (5814.3,18799.69)	4.00	1.6435 (0.887,2.868)	1.53 (0.15,2.93)
	Male	7751.97 (4238.08,14374.98)	3.23	1.1103 (0.607,2.0589)	1.23 (0.01,2.45)
15-49 years					
	Both	241399.26 (137487.23,419391.52)	8.46	6.1136 (3.4819,10.6213)	2.85 (1.4,4.33)
	Female	105646.07 (56996.8,192081.49)	8.42	5.4209 (2.9246,9.8561)	2.92 (1.53,4.33)
	Male	135753.19 (80475.08,231059.87)	8.50	6.7886 (4.0243,11.5546)	2.8 (1.3,4.33)
50-74 years					
	Both	138495.88 (74822.84,252951.94)	8.31	8.4326 (4.5558,15.4015)	1.36 (-0.04,2.78)

Female	53203.26 (27282.09,100063.91)	9.38	6.3193 (3.2405,11.8853)	1.81 (0.45,3.2)
Male	85292.62 (46801.82,152241.52)	7.74	10.6554 (5.8468,19.0191)	1.11 (-0.31,2.55)
75+ years				
Both	31269.92 (16819.31,59181.27)	7.98	10.8377 (5.8293,20.5114)	1.14 (-0.27,2.56)
Female	13796.75 (7378.02,26237.3)	8.07	8.237 (4.4049,15.6643)	1.45 (0.12,2.8)
Male	17473.18 (9292.29,32539.59)	7.92	14.4368 (7.6776,26.8852)	0.74 (-0.71,2.22)
**XDR-TB**				
<5 years				
Both	441.64 (294.61,635)	7.48	0.0671 (0.0448,0.0965)	5.18 (3.56,6.83)
Female	239.23 (159.74,346.72)	8.01	0.0752 (0.0502,0.109)	5.45 (3.83,7.1)
Male	202.41 (134.49,289.92)	6.93	0.0595 (0.0396,0.0853)	4.88 (3.25,6.54)
5-14 years				
Both	751.06 (458.17,1207.59)	10.49	0.0555 (0.0338,0.0892)	6.83 (5.33,8.36)
Female	408.54 (248.05,665.83)	10.44	0.0623 (0.0378,0.1016)	6.7 (5.14,8.28)
Male	342.52 (209.21,547.57)	10.55	0.0491 (0.03,0.0784)	7 (5.56,8.46)
15-49 years				
Both	12860.96 (8757.56,18740.14)	23.43	0.3257 (0.2218,0.4746)	8.19 (5.94,10.49)
Female	4610.84 (3035.95,7017.01)	21.77	0.2366 (0.1558,0.3601)	8.29 (6.27,10.36)
Male	8250.12 (5647.03,11781.27)	24.47	0.4126 (0.2824,0.5891)	8.15 (5.78,10.58)
50-74 years				
Both	7806.54 (5153.08,11938.53)	25.27	0.4753 (0.3138,0.7269)	6.56 (4.5,8.65)
Female	2560.55 (1644.48,4021.77)	26.11	0.3041 (0.1953,0.4777)	6.9 (4.96,8.87)
Male	5245.99 (3486.76,7929.76)	24.87	0.6554 (0.4356,0.9906)	6.41 (4.31,8.55)
75+ years				
Both	2175.87 (1321.85,3555.5)	36.44	0.7541 (0.4581,1.2323)	6.27 (4.13,8.46)
Female	931.35 (574.18,1562.95)	36.14	0.556 (0.3428,0.9331)	6.62 (4.56,8.72)
Male	1244.53 (750.65,1993.92)	36.68	1.0283 (0.6202,1.6474)	5.85 (3.66,8.09)
**HIV/AIDS-MDR-TB**				
<5 years				
Both	701.63 (463.51,1032.97)	4.63	0.1066 (0.0704,0.1569)	1.04 (-2.4,4.6)
Female	349.57 (230.91,513.11)	4.63	0.1099 (0.0726,0.1613)	1.04 (-2.4,4.6)
Male	352.06 (232.92,519.56)	4.64	0.1036 (0.0685,0.1528)	1.04 (-2.39,4.6)
5-14 years				
Both	1378.07 (941.15,2036.63)	162.47	0.1018 (0.0695,0.1505)	11.85 (7.78,16.08)
Female	687.18 (466.06,1018.99)	172.09	0.1048 (0.0711,0.1555)	11.93 (7.79,16.22)
Male	690.89 (474.18,1021.72)	153.91	0.099 (0.0679,0.1463)	11.79 (7.77,15.95)
15-49 years				
Both	33838.87 (23279.44,49655.63)	21.89	0.857 (0.5896,1.2576)	4.73 (2.07,7.46)
Female	18265.93 (12535.13,27335.29)	24.46	0.9373 (0.6432,1.4026)	4.9 (2.15,7.73)
Male	15572.95 (10709.62,22803.77)	19.47	0.7788 (0.5356,1.1403)	4.54 (1.98,7.16)
50-74 years				
Both	9211.91 (6326.05,13544.75)	39.26	0.5609 (0.3852,0.8247)	5.37 (2.86,7.95)
Female	4394.04 (3032.32,6424.23)	49.25	0.5219 (0.3602,0.7631)	5.7 (2.92,8.54)
Male	4817.87 (3212.68,7239.32)	33.08	0.6019 (0.4014,0.9044)	5.13 (2.83,7.48)

75+ years				
Both	458.91 (294.59,726.25)	13.49	0.1591 (0.1021,0.2517)	2.54 (0.75,4.36)
Female	239.34 (155.28,401.88)	33.79	0.1429 (0.0927,0.2399)	4.92 (2.62,7.28)
Male	219.58 (134.13,372.71)	7.85	0.1814 (0.1108,0.3079)	0.9 (-0.66,2.48)
**HIV/AIDS-XDR-TB**				
<5 years				
Both	17.54 (12.66,23.63)	24.06	0.0027 (0.0019,0.0036)	6.57 (2.68,10.61)
Female	8.64 (6.2,11.66)	24.41	0.0027 (0.0019,0.0037)	6.62 (2.72,10.67)
Male	8.9 (6.46,11.94)	23.72	0.0026 (0.0019,0.0035)	6.52 (2.64,10.55)
5-14 years				
Both	21.23 (15.28,29.09)	95.50	0.0016 (0.0011,0.0021)	13.29 (9.73,16.96)
Female	10.48 (7.48,14.4)	103.80	0.0016 (0.0011,0.0022)	13.74 (10.1,17.5)
Male	10.75 (7.75,14.68)	88.58	0.0015 (0.0011,0.0021)	12.89 (9.4,16.49)
15-49 years				
Both	1254.21 (904.7,1699.66)	48.71	0.0318 (0.0229,0.043)	10.21 (7.05,13.47)
Female	477.1 (348.16,638.39)	63.30	0.0245 (0.0179,0.0328)	11.38 (8.07,14.78)
Male	777.11 (551.32,1068.59)	42.61	0.0389 (0.0276,0.0534)	9.61 (6.5,12.81)
50-74 years				
Both	300.26 (206.94,425.22)	77.19	0.0183 (0.0126,0.0259)	10.1 (7.17,13.11)
Female	119.16 (85.67,165.74)	111.42	0.0142 (0.0102,0.0197)	10.94 (7.71,14.27)
Male	181.11 (122.18,267.12)	64.38	0.0226 (0.0153,0.0334)	9.63 (6.86,12.47)
75+ years				
Both	13.18 (6.98,27.97)	27.04	0.0046 (0.0024,0.0097)	6.82 (4.69,9)
Female	4.61 (2.82,8.31)	45.10	0.0028 (0.0017,0.005)	8.18 (5.67,10.75)
Male	8.57 (4.22,19.21)	22.81	0.0071 (0.0035,0.0159)	6.02 (4.04,8.04)

Abbreviations: MDR-TB=multidrug-resistant tuberculosis. XDR-TB=extensively drug-resistant tuberculosis. ASR=age-standardized rate. EAPC=estimated annual percentage change.

Table S15 The global death of cases and ASR of drug-resistant tuberculosis in 2021, and the trends from baseline to 2021 by sex and age group.

Age	Number_2021	Fold change	Death rate_2021	EAPC
**MDR-TB**				
<5 years				
Both	4545.95 (1891.74,9117.07)	1.55	0.6907 (0.2874,1.3852)	0.36 (-1.15,1.9)
Female	2536.16 (1076.43,4952.86)	1.67	0.7971 (0.3383,1.5566)	0.51 (-1.04,2.07)
Male	2009.79 (761.22,4028.8)	1.41	0.5912 (0.2239,1.185)	0.19 (-1.28,1.68)
5-14 years				
Both	1341.46 (566.51,2617.71)	2.99	0.0991 (0.0418,0.1934)	1.07 (-0.6,2.76)
Female	761.96 (307.43,1459.27)	3.34	0.1162 (0.0469,0.2226)	1.28 (-0.47,3.05)
Male	579.5 (236.26,1144.57)	2.60	0.083 (0.0338,0.1639)	0.81 (-0.77,2.42)
15-49 years				
Both	33284.81 (13024.72,67070.34)	6.88	0.843 (0.3299,1.6986)	2.44 (0.67,4.25)
Female	11981.97 (4398.75,24639.05)	6.23	0.6148 (0.2257,1.2643)	2.21 (0.53,3.91)

Male	21302.83 (8580.15,42257.46)	7.30	1.0653 (0.4291,2.1132)	2.58 (0.75,4.45)
50-74 years				
Both	47154.52 (18138.25,94148.16)	5.65	2.8711 (1.1044,5.7324)	0.85 (-0.69,2.41)
Female	16514.74 (6177.13,34352.21)	5.89	1.9616 (0.7337,4.0803)	1.15 (-0.3,2.63)
Male	30639.78 (11928.17,60911.65)	5.53	3.8277 (1.4902,7.6095)	0.7 (-0.88,2.3)
75+ years				
Both	20491.24 (7697.32,40929.56)	6.79	7.102 (2.6678,14.1856)	0.8 (-0.57,2.19)
Female	8930.93 (3345.09,18314.68)	6.81	5.332 (1.9971,10.9343)	1.08 (-0.2,2.39)
Male	11560.31 (4425.38,23924.97)	6.78	9.5515 (3.6564,19.7675)	0.43 (-0.99,1.88)
		**XDR-TB**		
<5 years				
Both	182.68 (78.82,361.65)	8.42	0.0278 (0.012,0.0549)	4.34 (2.22,6.5)
Female	94.36 (40.2,185.3)	8.64	0.0297 (0.0126,0.0582)	4.46 (2.34,6.62)
Male	88.32 (37.91,177.85)	8.19	0.026 (0.0112,0.0523)	4.22 (2.1,6.38)
5-14 years				
Both	70.21 (27.92,139.44)	14.10	0.0052 (0.0021,0.0103)	5 (2.64,7.41)
Female	39.24 (15.18,78.2)	15.70	0.006 (0.0023,0.0119)	5.27 (2.86,7.74)
Male	30.97 (12.59,61.04)	12.47	0.0044 (0.0018,0.0087)	4.68 (2.37,7.04)
15-49 years				
Both	2442.17 (1074.75,4598.74)	32.75	0.0618 (0.0272,0.1165)	6.9 (3.98,9.91)
Female	790.52 (315.26,1532.09)	31.06	0.0406 (0.0162,0.0786)	7.04 (4.36,9.78)
Male	1651.65 (753.78,3092.82)	33.63	0.0826 (0.0377,0.1547)	6.85 (3.81,9.97)
50-74 years				
Both	3711.23 (1561.66,7030.69)	29.92	0.226 (0.0951,0.4281)	5.78 (3.31,8.31)
Female	1105.61 (416.03,2117.93)	30.01	0.1313 (0.0494,0.2516)	6.02 (3.71,8.38)
Male	2605.62 (1141.06,4954.38)	29.88	0.3255 (0.1425,0.6189)	5.69 (3.16,8.28)
75+ years				
Both	1540.15 (575.29,3032.33)	35.09	0.5338 (0.1994,1.051)	5.83 (3.68,8.04)
Female	647.71 (241.31,1243.67)	34.73	0.3867 (0.1441,0.7425)	6.16 (4.1,8.26)
Male	892.43 (336.16,1781.96)	35.35	0.7374 (0.2777,1.4723)	5.43 (3.21,7.7)
		**HIV/AIDS-MDR-TB**		
<5 years				
Both	510.01 (197.2,1006.73)	4.41	0.0775 (0.03,0.153)	0.84 (-2.64,4.44)
Female	254.56 (98.74,502.03)	4.42	0.08 (0.031,0.1578)	0.85 (-2.63,4.45)
Male	255.46 (98.45,504.7)	4.40	0.0751 (0.029,0.1485)	0.83 (-2.65,4.43)
5-14 years				
Both	535.89 (226.37,1011.98)	103.26	0.0396 (0.0167,0.0748)	11.58 (7.57,15.75)
Female	265.22 (110.04,498.06)	110.91	0.0405 (0.0168,0.076)	11.74 (7.65,15.98)
Male	270.67 (115.29,507.46)	96.71	0.0388 (0.0165,0.0727)	11.44 (7.48,15.54)
15-49 years				
Both	12499.13 (5542.76,22856.89)	24.55	0.3165 (0.1404,0.5789)	5.25 (1.94,8.66)
Female	6840.65 (2992.73,12669.33)	30.00	0.351 (0.1536,0.6501)	5.39 (1.95,8.94)
Male	5658.47 (2518.73,10392.93)	20.07	0.283 (0.126,0.5197)	5.11 (1.95,8.38)
50-74 years				

Both	3771.28 (1672.16,7271.15)	34.71	0.2296 (0.1018,0.4427)	4.82 (1.59,8.16)
Female	1718.12 (738.8,3349.06)	38.89	0.2041 (0.0878,0.3978)	4.66 (1.2,8.23)
Male	2053.17 (893.54,3797.98)	31.83	0.2565 (0.1116,0.4745)	4.99 (1.97,8.11)
75+ years				
Both	141.27 (60.58,270.31)	26.59	0.049 (0.021,0.0937)	2.84 (-0.29,6.07)
Female	66.03 (27.95,131.86)	40.27	0.0394 (0.0167,0.0787)	4.05 (0.56,7.65)
Male	75.24 (32.83,143.44)	20.38	0.0622 (0.0271,0.1185)	1.87 (-1.04,4.85)
**HIV/AIDS-XDR-TB**				
<5 years				
Both	12.67 (5.68,23.09)	24.34	0.0019 (9e-04,0.0035)	6.32 (2.36,10.44)
Female	6.23 (2.78,11.38)	24.96	0.002 (9e-04,0.0036)	6.38 (2.41,10.51)
Male	6.43 (2.87,11.69)	23.73	0.0019 (8e-04,0.0034)	6.27 (2.32,10.37)
5-14 years				
Both	11.57 (5,21.02)	88.00	9e-04 (4e-04,0.0016)	12.41 (8.57,16.39)
Female	5.69 (2.48,10.31)	93.83	9e-04 (4e-04,0.0016)	12.9 (8.96,16.97)
Male	5.88 (2.53,10.72)	83.00	8e-04 (4e-04,0.0015)	11.99 (8.23,15.89)
15-49 years				
Both	624.92 (291.93,1101.48)	82.77	0.0158 (0.0074,0.0279)	10.4 (6.58,14.35)
Female	248.78 (112.66,435.48)	121.55	0.0128 (0.0058,0.0223)	11.84 (7.83,15.99)
Male	376.14 (175.78,656.32)	68.27	0.0188 (0.0088,0.0328)	9.66 (5.91,13.54)
50-74 years				
Both	183.93 (81.32,340.69)	114.68	0.0112 (0.005,0.0207)	10.07 (6.63,13.63)
Female	69.65 (32.1,131.42)	144.10	0.0083 (0.0038,0.0156)	10.67 (6.94,14.52)
Male	114.28 (48.57,212.52)	101.95	0.0143 (0.0061,0.0265)	9.74 (6.45,13.13)
75+ years				
Both	6.91 (2.53,16.12)	61.82	0.0024 (9e-04,0.0056)	7.07 (3.99,10.24)
Female	2.2 (0.89,4.54)	72.33	0.0013 (5e-04,0.0027)	8.06 (4.61,11.63)
Male	4.71 (1.56,11.58)	57.88	0.0039 (0.0013,0.0096)	6.42 (3.49,9.42)

Abbreviations: MDR-TB=multidrug-resistant tuberculosis. XDR-TB=extensively drug-resistant tuberculosis. ASR=age-standardized rate. EAPC=estimated annual percentage change.

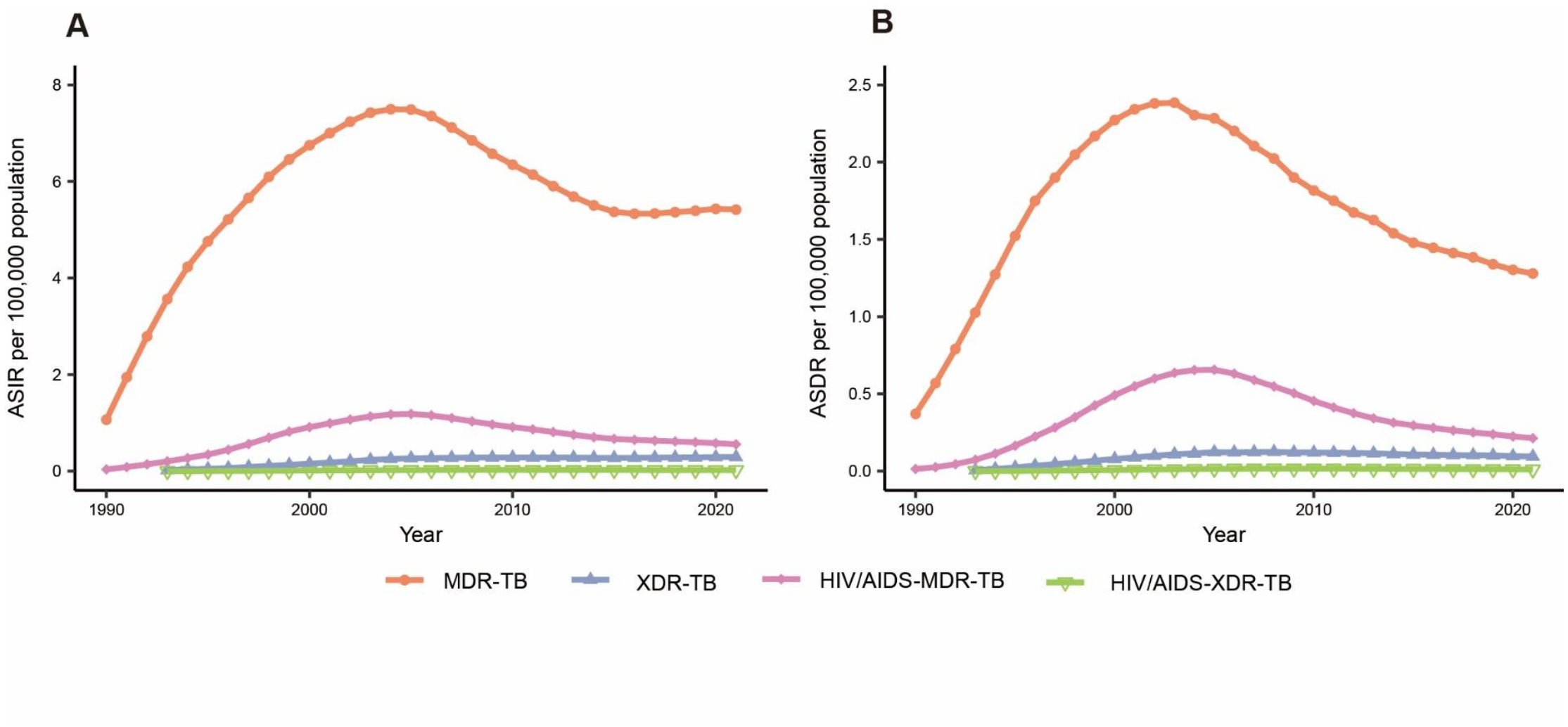

Figure S1 The ASIR (A) and ASDR (B) per 100,000 population of MDR-TB, XDR-TB, HIV/AIDS-MDR-TB, and HIV/AIDS-XDR-TB globally from baseline to 2021

MDR-TB=multidrug-resistant tuberculosis. XDR-TB=extensively drug-resistant tuberculosis. ASIR=age-standardized incidence rate. ASDR=age-standardized death rate.

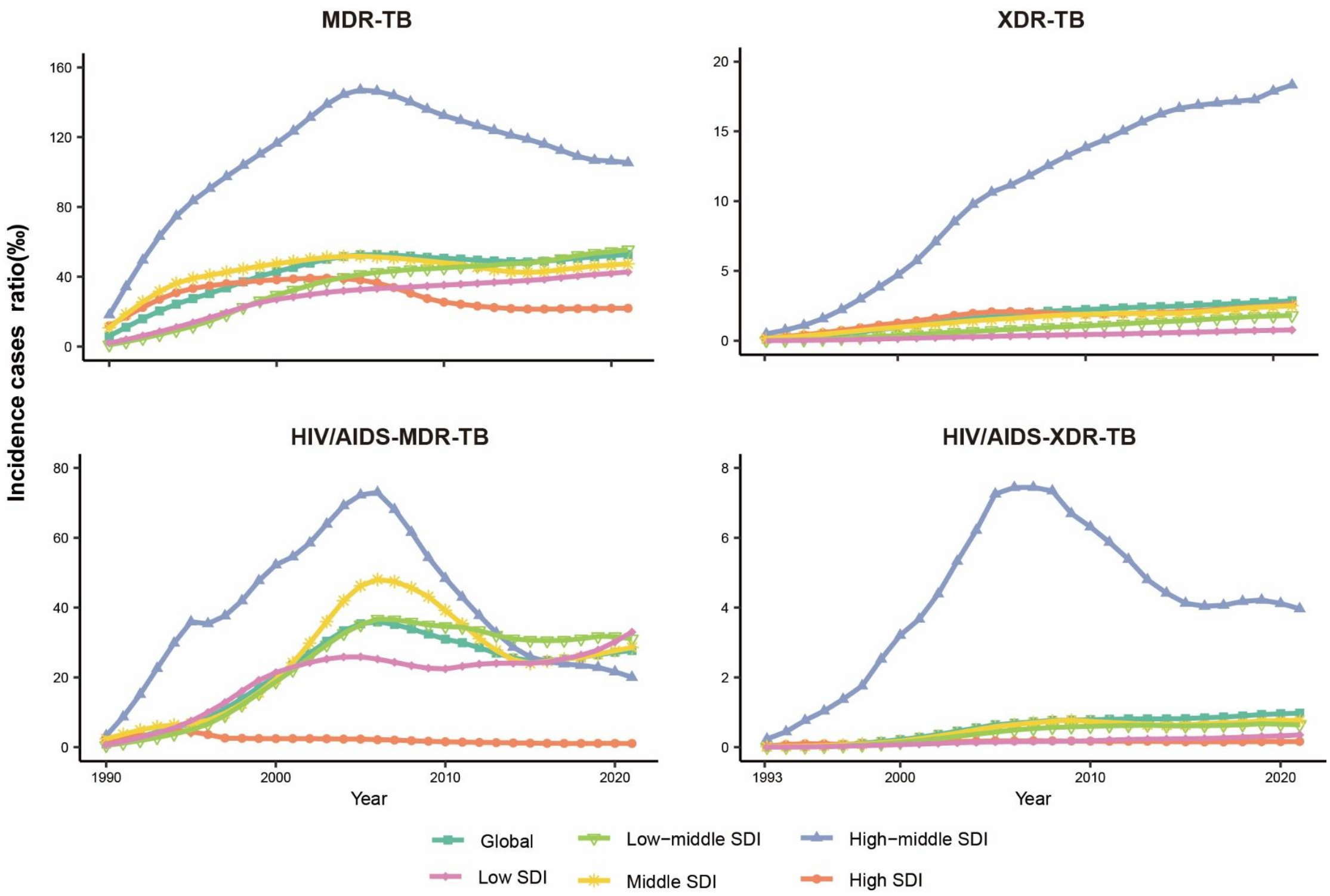

Figure S2 The incidence ratio of MDR-TB, XDR-TB, HIV/AIDS-MDR-TB, and HIV/AIDS-MDR-TB from baseline to 2021 globally and in 5 territories

MDR-TB=multidrug-resistant tuberculosis. XDR-TB=extensively drug-resistant tuberculosis.

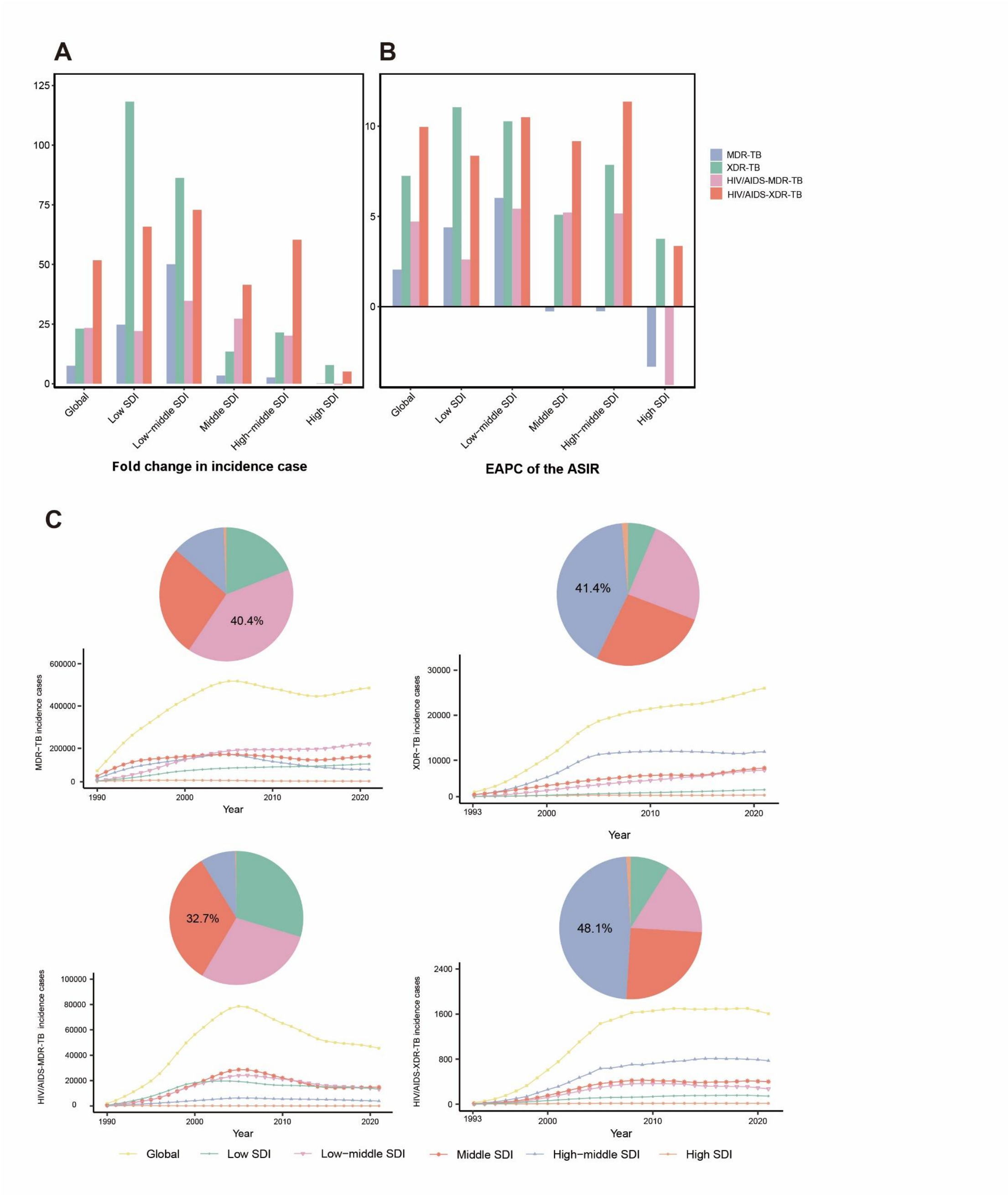

Figure S3 Temporal Trend of MDR-TB, XDR-TB, HIV/AIDS-MDR-TB, and HIV/AIDS-XDR-TB in global and 5 territories. (A) Fold change in incidence cases from baseline to 2021. (B) The EAPC of ASIR from baseline to 2021. (C) The incidence cases of MDR-TB, XDR-TB, HIV/AIDS-MDR-TB, and HIV/AIDS-XDR-TB from baseline to 2021(line chart) and the percentage distribution of global MDR-TB, XDR-TB, HIV/AIDS-MDR-TB, and HIV/AIDS-XDR-TB incidence cases across the 5 SDI regions in 2021 (pie chart)

MDR-TB=multidrug-resistant tuberculosis. XDR-TB=extensively drug-resistant tuberculosis. ASIR=age-standardized incidence rate. EAPC=estimated annual percentage change. SDI=Social Demographic

Index.

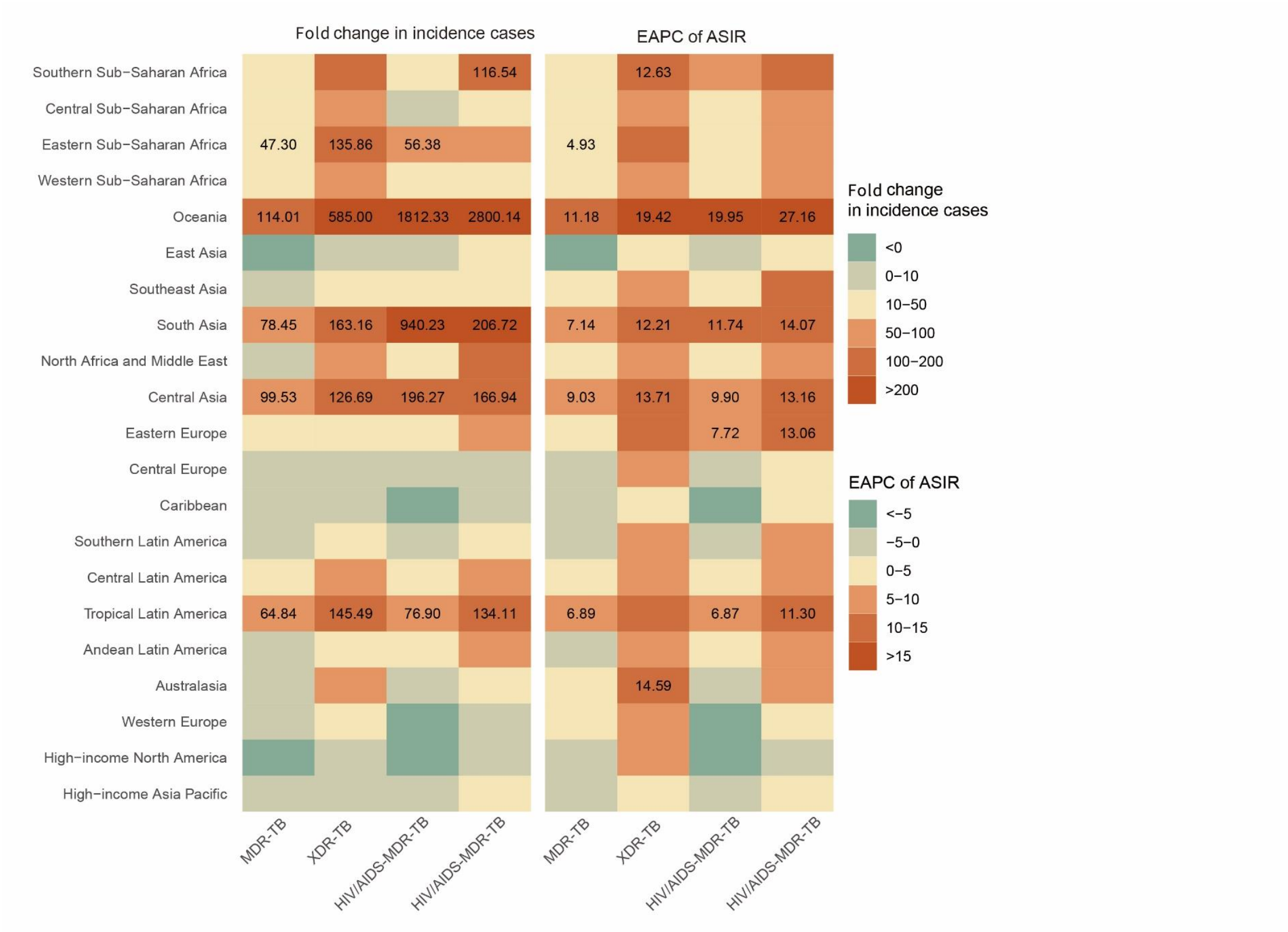

Figure S4 Temporal trend of MDR-TB, XDR-TB, HIV/AIDS-MDR-TB, and HIV/AIDS-XDR-TB in regions. (A) Fold change in incidence cases from baseline to 2021. (B) The EAPC of ASIR from baseline to 2021

MDR-TB=multidrug-resistant tuberculosis. XDR-TB=extensively drug-resistant tuberculosis. ASIR=age-standardized incidence rate. EAPC=estimated annual percentage change.

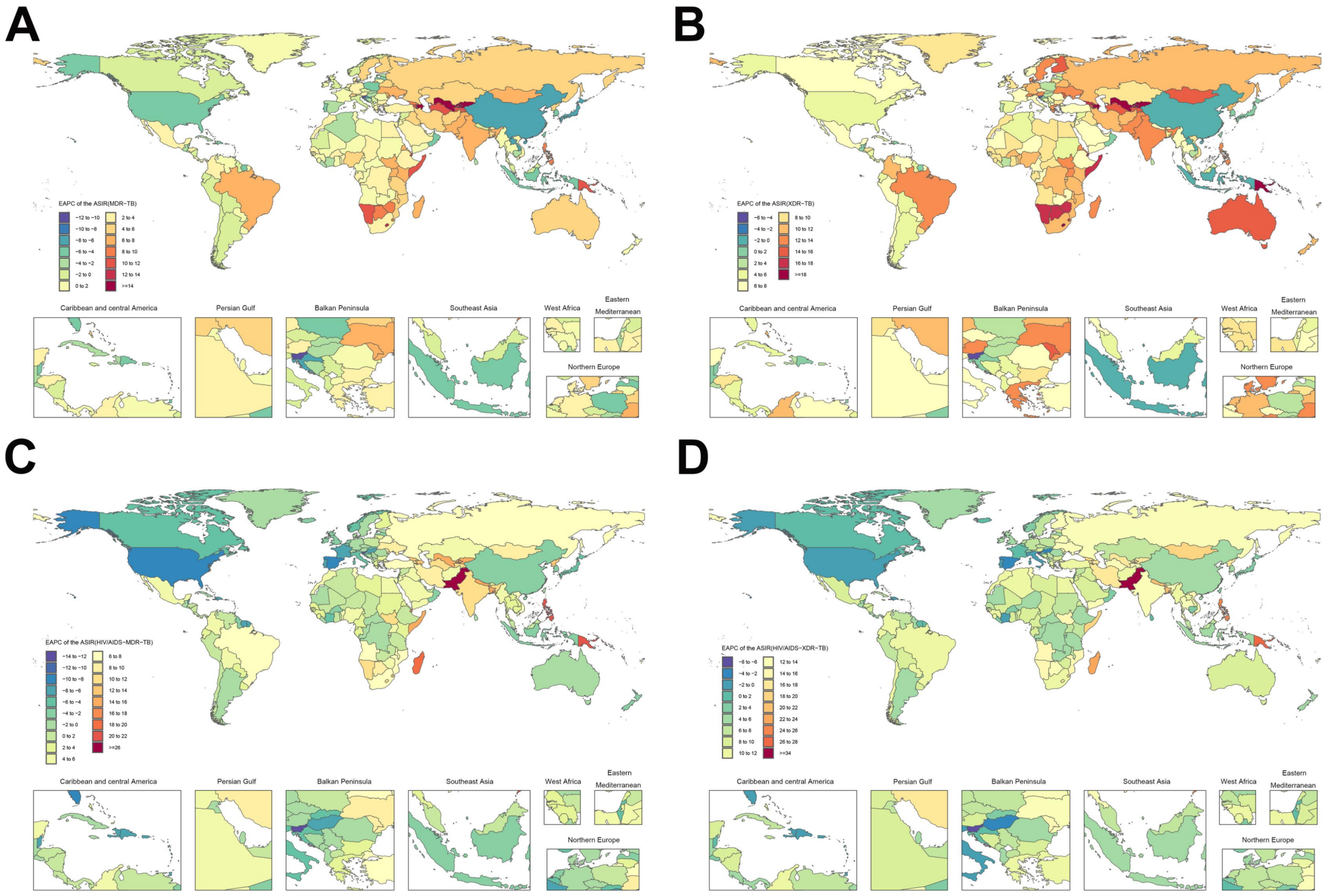

Figure S5 Temporal trend of the EAPC of ASIR for MDR-TB (A), XDR-TB (B), HIV/AIDS-MDR-TB (C), and HIV/AIDS-XDR-TB (D) from baseline to 2021 across 204 countries

MDR-TB=multidrug-resistant tuberculosis. XDR-TB=extensively drug-resistant tuberculosis. ASIR=age-standardized incidence rate. EAPC=estimated annual percentage change.

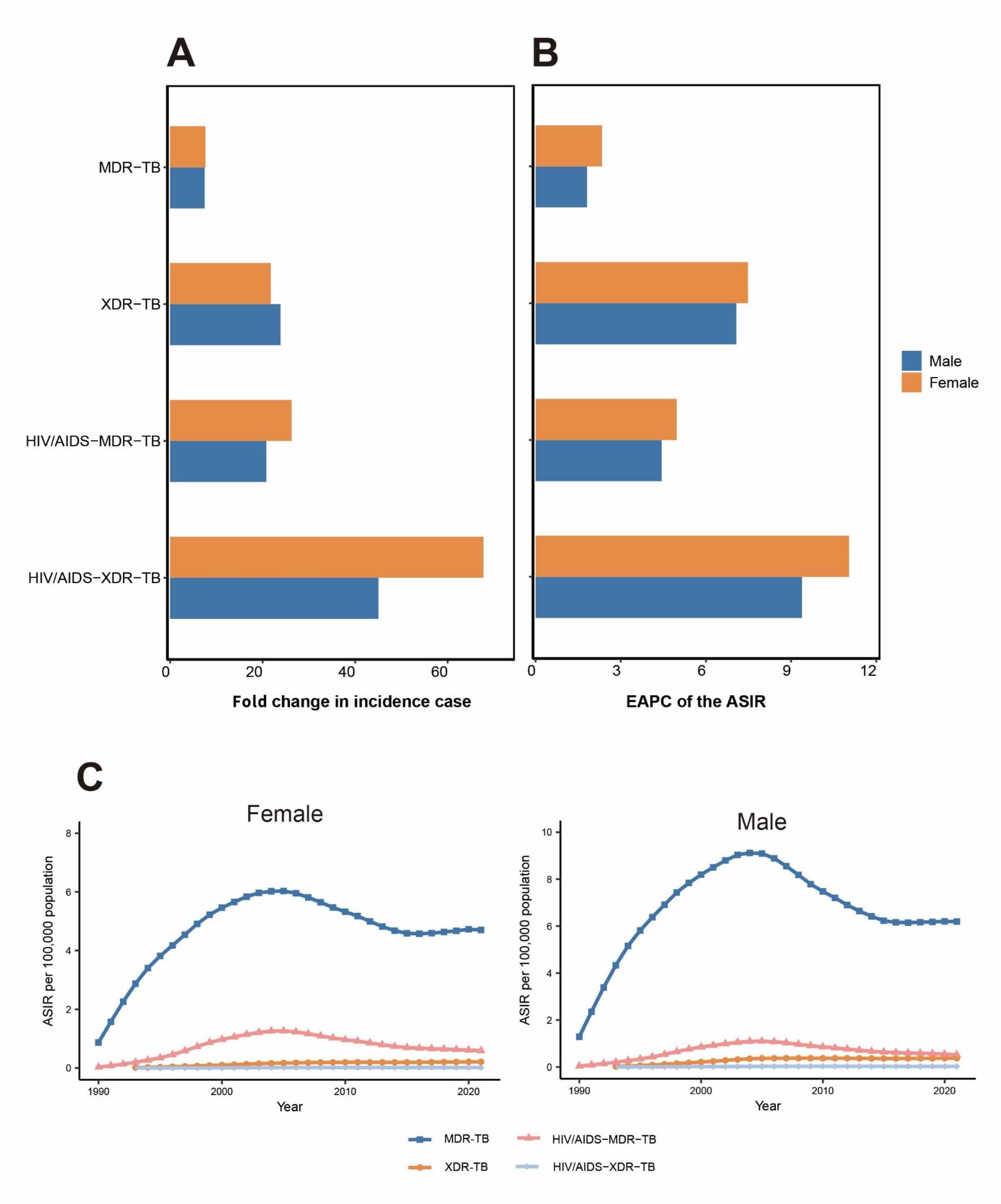

Figure S6 Temporal Trend of MDR-TB, XDR-TB, HIV/AIDS-MDR-TB, and HIV/AIDS-XDR-TB globally by sex pattern. (A) Fold change in incidence cases from baseline to 2021. (B) The EAPC of ASIR globally from baseline to 2021. (C) The ASIR per 100,000 population of MDR-TB, XDR-TB, HIV/AIDS-MDR-TB, and HIV/AIDS-XDR-TB globally from baseline to 2021

MDR-TB=multidrug-resistant tuberculosis. XDR-TB=extensively drug-resistant tuberculosis. ASIR=age-standardized incidence rate. EAPC=estimated annual percentage change.

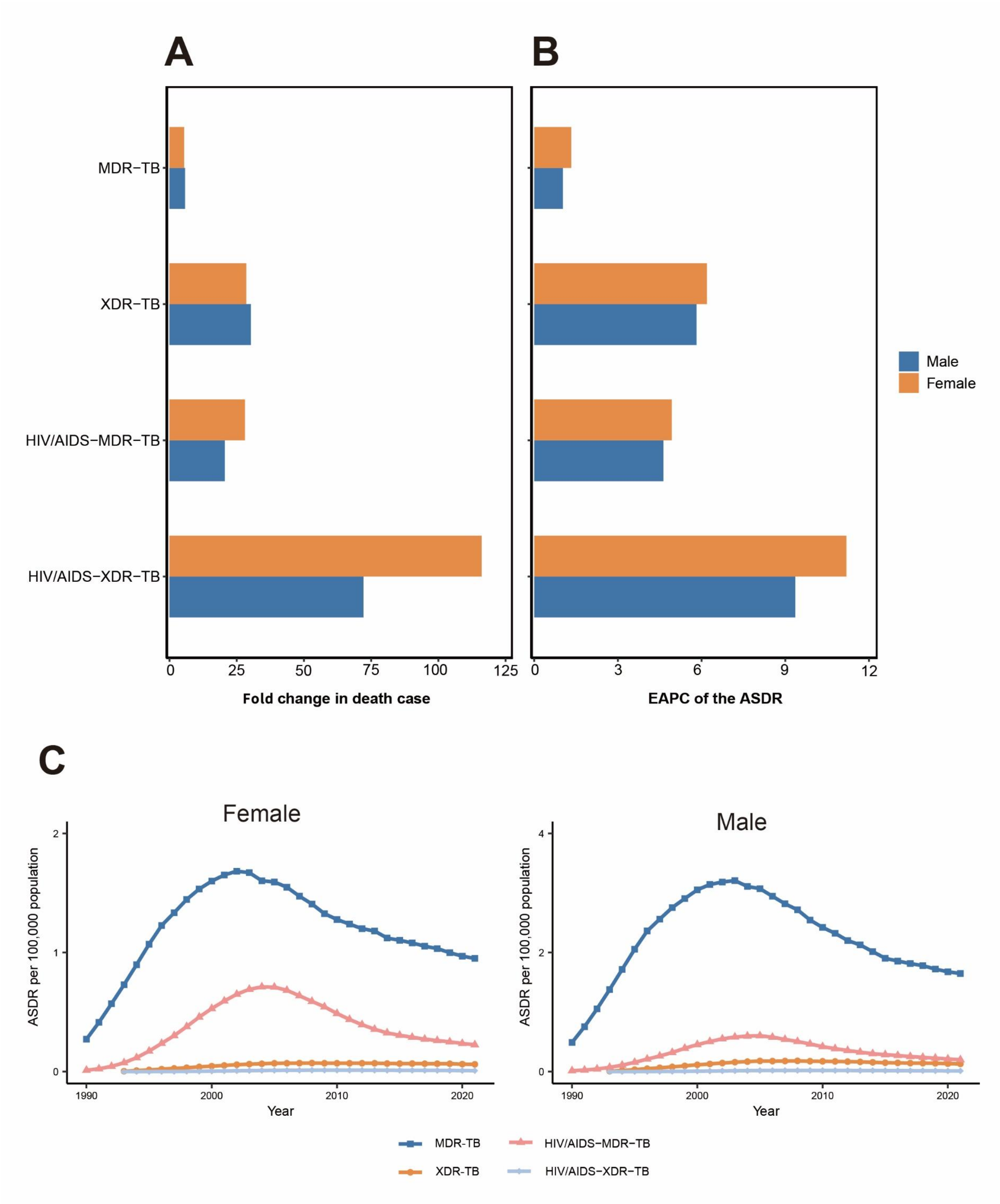

Figure S7 Temporal Trend of MDR-TB, XDR-TB, HIV/AIDS-MDR-TB, and HIV/AIDS-XDR-TB globally by sex pattern. (A) Fold change in death cases from baseline to 2021. (B) The EAPC of ASDR globally from baseline to 2021. (C) The ASDR per 100,000 population of MDR-TB, XDR-TB, HIV/AIDS-MDR-TB, and HIV/AIDS-XDR-TB globally from baseline to 2021.

MDR-TB=multidrug-resistant tuberculosis. XDR-TB=extensively drug-resistant tuberculosis. ASDR=age-standardized death rate. EAPC=estimated annual percentage change.

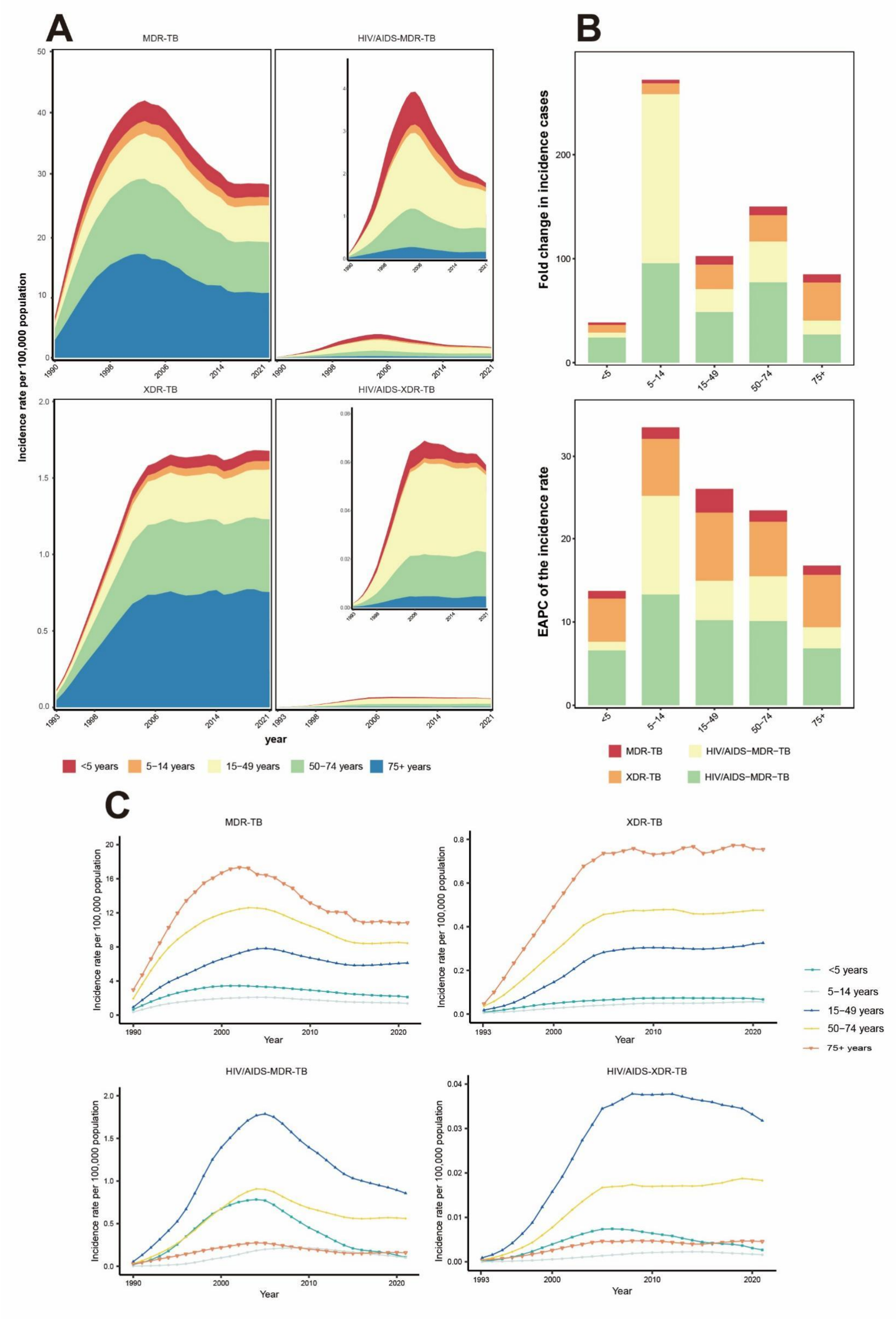

Figure S8 Global temporal trend of MDR-TB, XDR-TB, HIV/AIDS-MDR-TB, and HIV/AIDS-XDR-TB by age pattern. (A) Incidence cases in 5 age groups from baseline to 2021. (B) Fold change in incidence cases across 5 age groups globally from baseline to 2021. (C) The EAPC of ASDR for 5 age groups globally from baseline to 2021.

MDR-TB=multidrug-resistant tuberculosis. XDR-TB=extensively drug-resistant tuberculosis. ASIR=age-standardized incidence rate. EAPC=estimated annual percentage change.

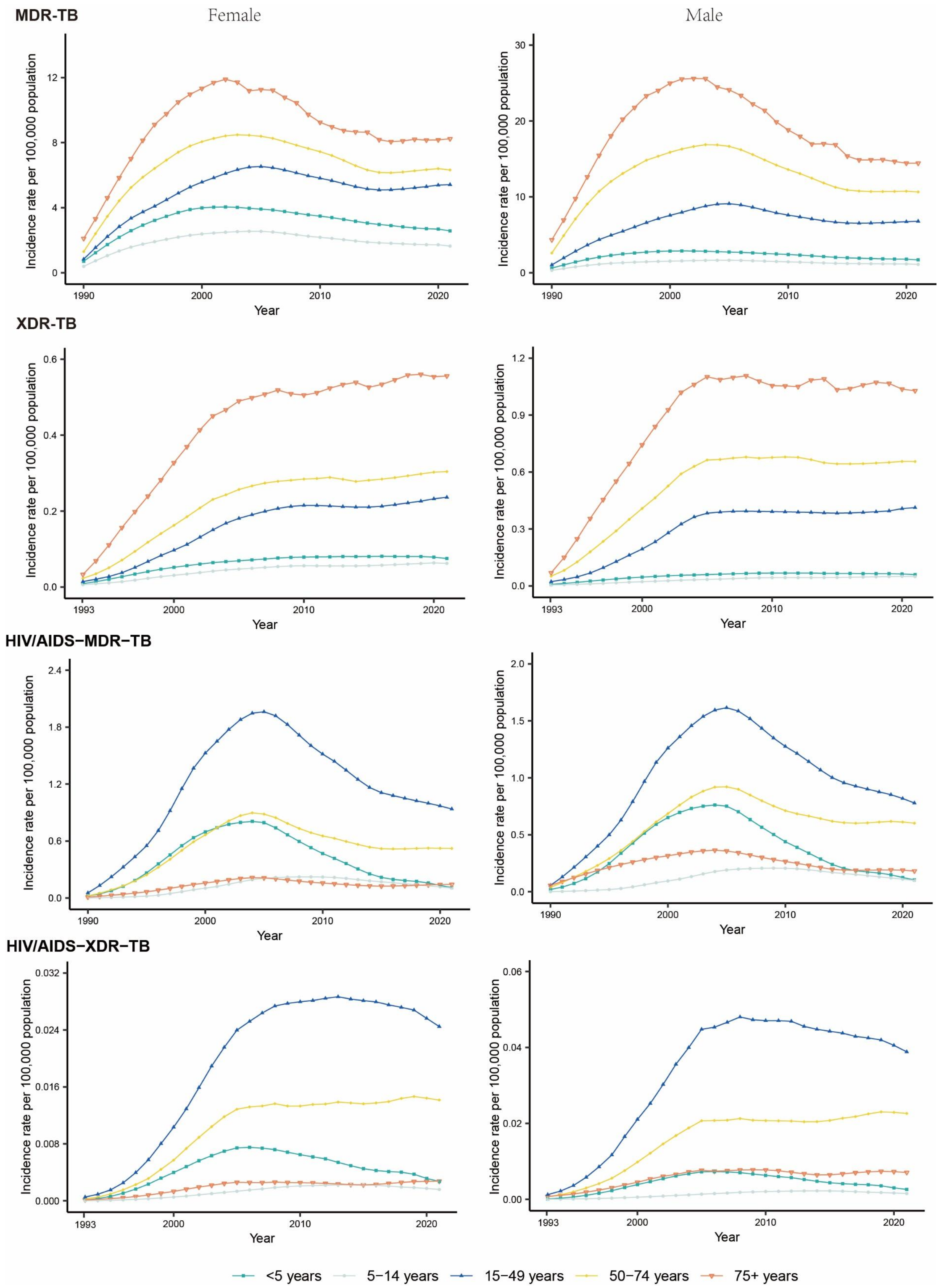

Figure S9 Global ASIR per 100,000 population for MDR-TB, XDR-TB, HIV/AIDS-MDR-TB, and HIV/AIDS-XDR-TB across 5 age groups from baseline to 2021, stratified by sex

MDR-TB=multidrug-resistant tuberculosis. XDR-TB=extensively drug-resistant tuberculosis. ASIR=age-standardized incidence rate. EAPC=estimated annual percentage change.

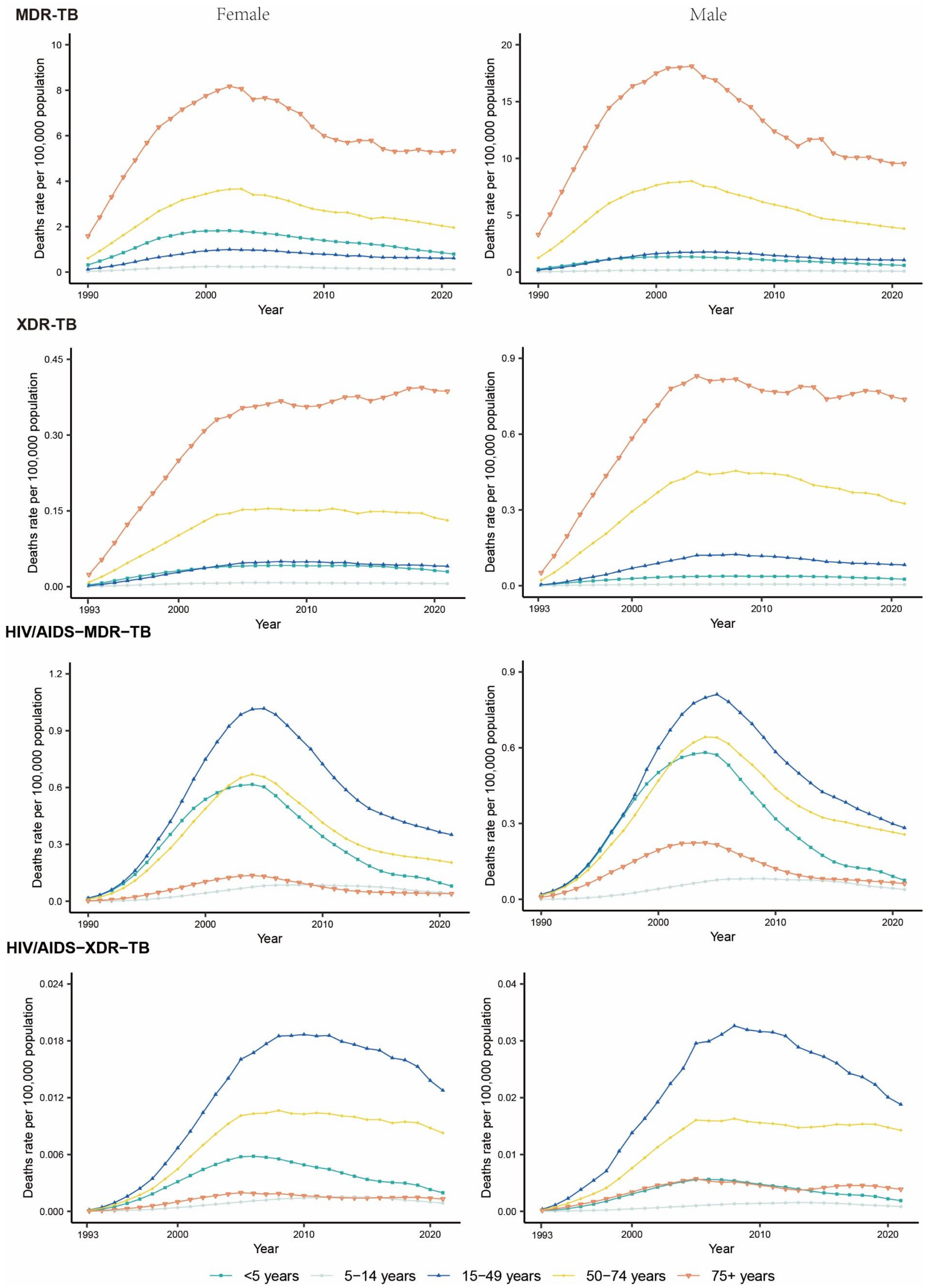

Figure S10 Global ASDR per 100,000 population for MDR-TB, XDR-TB, HIV/AIDS-MDR-TB, and HIV/AIDS-XDR-TB across 5 age groups from baseline to 2021, stratified by sex

MDR-TB=multidrug-resistant tuberculosis. XDR-TB=extensively drug-resistant tuberculosis. ASDR=age-standardized death rate. EAPC=estimated annual percentage change.

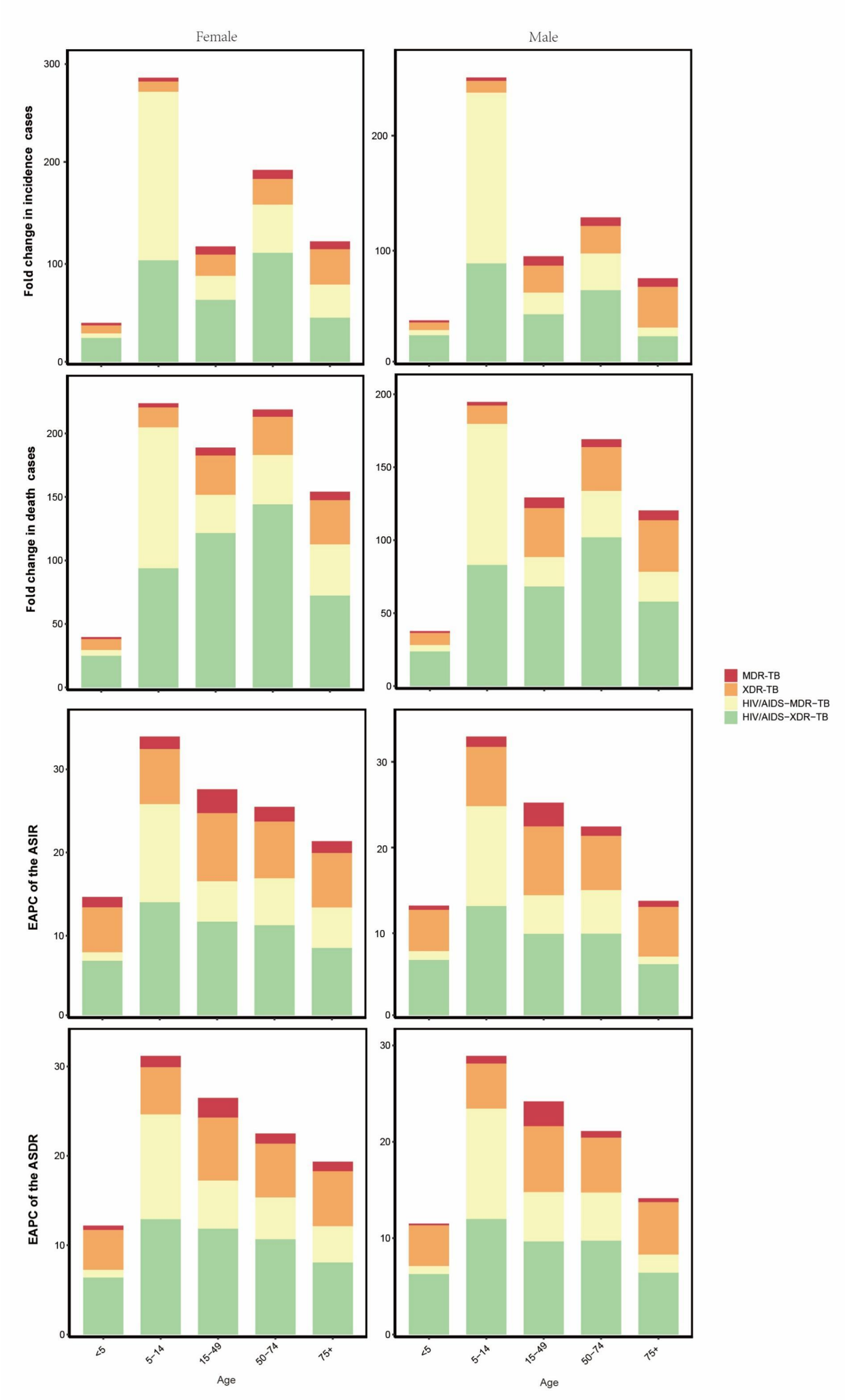
Female
Male
Fold change in incidence cases
Fold change in death cases
EAPC of the ASIR
EAPC of the ASDR
MDR-TB
XDR-TB
HIV/AIDS-MDR-TB
HIV/AIDS-XDR-TB
<5
5-14
15-49
50-74
75+
Age

Figure S11 Fold change in MDR-TB, XDR-TB, HIV/AIDS-MDR-TB, and HIV/AIDS-XDR-TB cases of incidence and death across 5 age groups globally from baseline to 2021, and the EAPC of ASIR and ASDR for 5 age groups globally from baseline to 2021, by sex

MDR-TB=multidrug-resistant tuberculosis. XDR-TB=extensively drug-resistant tuberculosis. ASIR=age-standardized incidence rate. ASDR=age-standardized death rate. EAPC=estimated annual percentage change.

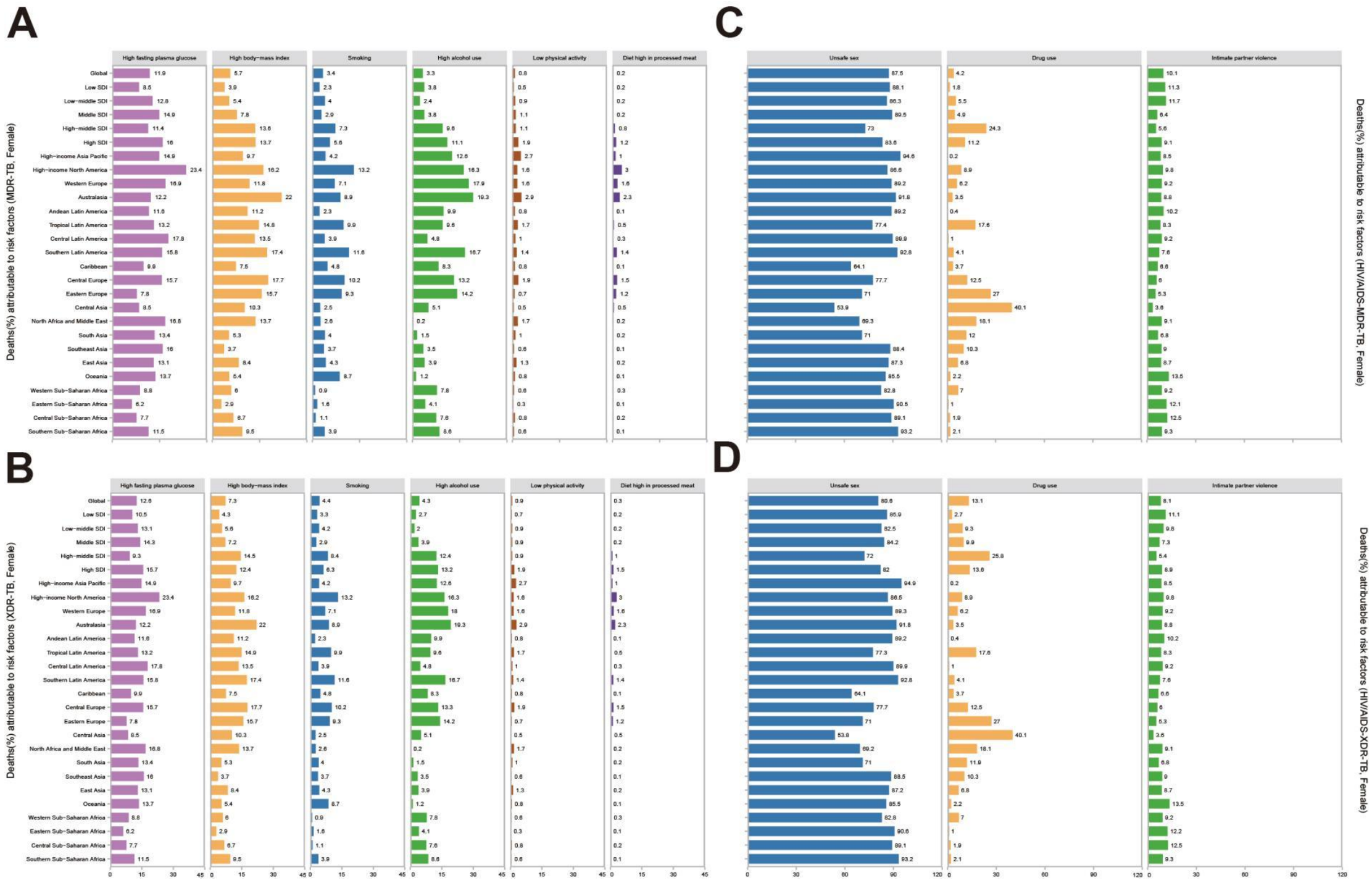

Figure S12 Percentage of death due to MDR-TB (A), XDR-TB (B), HIV/AIDS-MDR-TB(C), and HIV/AIDS-XDR-TB (D) attributable to significant risk factors in females globally, across 5 territories, and in 21 GBD regions in 2021 in females

MDR-TB=multidrug-resistant tuberculosis. XDR-TB=extensively drug-resistant tuberculosis. GBD=Global Burden of Disease.

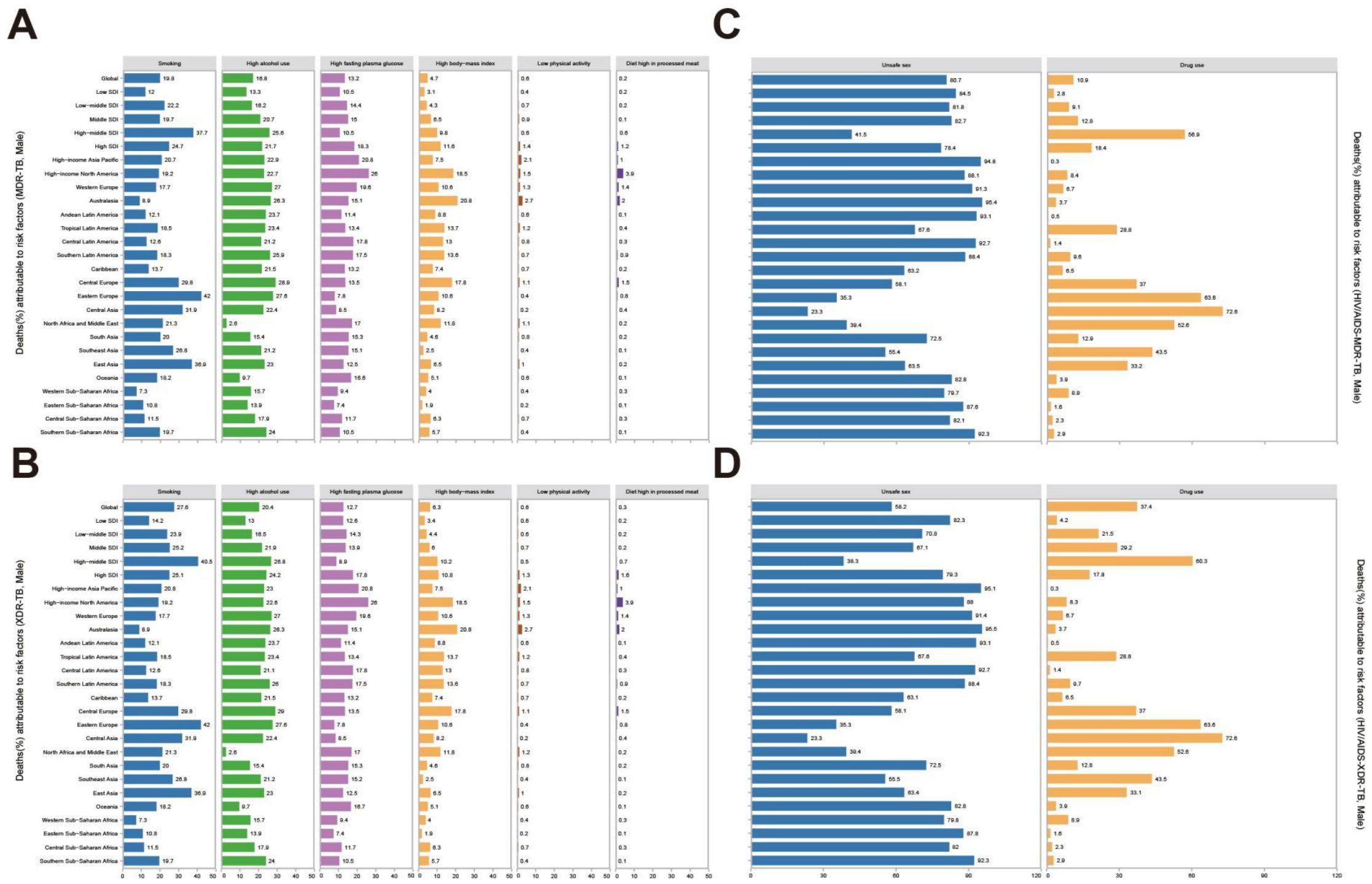

Figure S13 Percentage of death due to MDR-TB (A), XDR-TB (B), HIV/AIDS-MDR-TB(C), and HIV/AIDS-XDR-TB (D) attributable to significant risk factors in males globally, across 5 territories, and in 21 GBD regions in 2021 in females

MDR-TB=multidrug-resistant tuberculosis. XDR-TB=extensively drug-resistant tuberculosis. GBD=Global Burden of Disease.
